# Supplementary material for: Social Determinants of Health Curriculum for the Pediatric Clerkship
Source: MedEdPORTAL. 2024 Oct 29;20:11458. doi: 10.15766/mep_2374-8265.11458 (PMC11518917; doi:10.15766/mep_2374-8265.11458)
Supplement: Supplementary file 1 — SDH Cases Faculty Supplements.docxCurriculum Orientation.pptxSDH Cases Student Handouts.docxPrework - Well Child.pptxPrework - Urgent Care.pptxPrework - Clinical Problem-solving.pptxPrework - Chronic Illness.pptxResource Assignment Orientation.pptxResource Assignment Form and Example.docxFacilitator Reminder Email.docxPresurvey and Case Analysis.docxPostsurvey and Case Analysis.docxCase Analysis Scoring Tool.docx [file mep_2374-8265.11458-s001.zip › A. SDH Cases Faculty Supplements.docx]

**Instructions for use**: You should provide the email and/or print version of the introductory letter to faculty found on pages 1 and 2 prior to the beginning of the social determinants of health (SDH) curriculum. Faculty supplements on pages 3-6 correspond to each small group session. At our institution, the faculty receive a binder with these supplements in print version at the beginning of the student clerkship block. The email and print letters to small group leaders contain faculty instructions for use.

**[Email Version]**

Dear Small Group Leaders,

Hello. We are working to incorporate a curriculum on Social Determinants of Health (SDH) in pediatrics into the Pediatric didactic sessions. The most recent COMSEP standards for the pediatric clerkship include understanding the role of SDH in pediatric health. In addition to the COMSEP standards, we believe integrating education regarding SDH is important in the training of physicians to truly practice whole patient care.

There are five components to the SDH curriculum as described below. *The third, fourth, and fifth component will occur during the small groups and require your facilitation.*

1. Pre-survey:

- Administered via email by Clerkship Director prior to small group sessions.

2. Pre-group modules

- Students will complete interactive power points on SDH for common pediatric diagnoses prior to their Well Child, Urgent Care, Clinical Problem Solving, and Chronic Illness Small Groups.
- I am attaching these modules for your personal reference, but they should be completed by the students prior to the small groups and require no action from you.

**3. Small Group Case Discussions**

- During the Well Child, Urgent Care, Clinical Problem Solving, and Chronic Illness Small Groups we ask that you facilitate discussions on SDH cases.
- There will be one case for each of these small groups.
- You will be provided with a student case handout to give to the students during the small group. *(Attached to this email and included in the small group binder)*
- You will also be provided with a facilitator guide for each case that has potential answers to the prompts the students are given*. (Attached to this email and included in the small group binder)*
- Case discussions are designed to take 5-10 minutes.

**4. Resource Presentation**

- The students have been assigned the task to research a resource which could be offered to a pediatric patient/family to help mitigate a potential SDH.
- The assignment (“SDH Resource Assignment Form”) and example will be provided to the student ahead of time via their online learning platform and in their orientation packets.
- A reminder about the assignment is included on the “Social Determinants of Health Case: Clinical Problem Solving” handout.
- At the Chronic Illness Small Group students should present to the group the:
  - Name of the resource
  - Category/Categories of SDH the resource addresses
  - Brief description of the resource (1-3 sentences)
  - Brief explanation of how this resource could benefit a pediatric patient (1-3 sentences)
- They will also turn in a written copy of their completed assignment. They do not need to write their names on these.

**5. Post-Survey**

- Please allow students 10 minutes at the end of their Chronic Illness Small Group to complete this post-survey.
- The post-survey will be sent ahead of time to the students via email. The survey is voluntary, but we would like dedicated time provided for them to complete it.

Thank you! Please let me know if you have any questions or comments. We welcome your suggestions for improvement.

**[Print Version]**

Dear Small Group Leaders,

We are working to incorporate a curriculum on Social Determinants of Health (SDH) in pediatrics into the pediatric didactic sessions. The most recent COMSEP standards for the pediatric clerkship include understanding the role of SDH in pediatric health. In addition to the COMSEP standards, we believe integrating education regarding SDH is important in the training of physicians to truly practice whole patient care.

There are five components to the SDH curriculum as described below. *The third, fourth, and fifth component will occur during the small groups and require your facilitation.*

1. Pre-survey:

- Administered via email by Clerkship Director prior to small group sessions.

2. Pre-group modules

- Students will complete interactive power points on SDH for common pediatric diagnoses prior to their Well Child, Urgent Care, Clinical Problem Solving, and Chronic Illness Small Groups.
- These should be completed by the students prior to the small groups and require no action from you.

**3. Small Group Case Discussions**

- During the Well Child, Urgent Care, Clinical Problem Solving, and Chronic Illness Small Groups we ask that you facilitate discussions on SDH cases.
- There will be one case for each of these small groups.
- Titled “Social determinants of Health Case: [Small Group Title]”. Case to be given to the students during the small group. Format for discussion of case is flexible. Cases can be discussed in small groups of two to three students followed by whole group discussion or whole group discussion alone if time is limited. ***(Included in this binder)***
- You will also be provided with a facilitator guide for each case that has potential answers to the prompts the students are given***. (Included in this binder)***
- Case discussions are designed to take 5-10 minutes.

**4. Resource Presentation**

- The students have been assigned the task to research a resource which could be offered to a pediatric patient/family to help mitigate a potential SDH.
- The assignment (“SDH Resource Assignment Form”) and example will be provided to the student ahead of time via their online learning management platform and in their orientation packets.
- A reminder about the assignment is included on the “Social Determinants of Health Case: Clinical Problem Solving” handout.
- At the Chronic Illness Small Group students should present to the group the:
  - Name of the resource
  - Category/Categories of SDH the resource addresses
  - Brief description of the resource (1-3 sentences)
  - Brief explanation of how this resource could benefit a pediatric patient (1-3 sentences)
- They will also turn in a written copy of their completed assignment. They do not need to write their names on these.

**5. Post-Survey**

- Please allow students ~10 minutes at the end of their Chronic Illness Small Group to complete an online post-survey.
- The post-survey will be sent ahead of time to the students via email. The survey is voluntary, but we would like dedicated time provided for them to complete it.

Thank you! Please let me know if you have any questions or comments.

**Instructions for use:** You can provide this summary to small group facilitators for reference so they understand the key learning points on SDH that students should have attained via the pre-work PowerPoints.

Summary of Learning Points for Student Pre-Work

Pre-work PowerPoint Well Child (Appendix D)

- SDH is defined as “Social determinants of health…are the conditions in the environments where people are born, live, learn, work, play, worship, and age that affect a wide range of health, functioning, and quality-of-life outcomes and risks.”
  - Per Healthy People 2030, U.S. Department of Health and Human Services, Office of Disease Prevention and Health Promotion. “Social Determinants of Health.” Retrieved 10/30/20, from <https://health.gov/healthypeople/objectives-and-data/social-determinants-health>
- Examples of SDH categories
  - Availability of resources to meet daily needs
  - Access to health care services
  - Transportation options
  - Public safety
  - Social support
  - Exposure to crime, violence, and social disorder
  - Socioeconomic conditions
  - Language/Literacy
  - Access to mass media and emerging technologies
- SDH related to socioeconomic conditions, access to food, and access to physical activity facilities have been shown to impact obesity and related factors in children.

Pre-work PowerPoint Urgent Care (Appendix E)

- SDH related to food insecurity, social support, safe housing, Adverse Childhood Experiences, and racism have been shown to impact asthma in children.

Pre-work PowerPoint Clinical Problem Solving (Appendix F)

- SDH related to language/literacy, access to health services, and socioeconomic conditions have been shown to impact dental care and dental caries in children.

Pre-work PowerPoint Chronic Illness (Appendix G)

- SDH related to socioeconomic conditions, access to health care services, and access to emerging technologies and language/literacy have been shown to impact factors related to management and control of diabetes mellitus in children.

**Social Determinants of Health Case: Well Child (Faculty Supplement)**

A 3-month-old male infant comes to see his pediatrician for a well child visit. He has not been seen since his initial newborn visit. He has fallen from the 40^th^ to 25^th^ percentile for weight. His mother stopped breastfeeding a month ago when she went back to work. She reports mixing the formula 1 scoop to 2 oz but sometimes she adds extra water to make the formula go farther.

**What social determinants of health might be a factor in this scenario?**

Potential SDHs:

A. Socioeconomic conditions

a. Inability to pay for formula

b. Cost of pump

B. Social support

a. Mother had to go back to work

C. Availability of resources to meet daily needs

a. Access to area at work to pump

D. Transportation

a. Potential difficulty getting to PCP for follow up visits or to WIC office

**What kinds of questions could you ask the patient’s family to discover what social determinants of health might be affecting him?**

Potential SDH questions:

A. Many of our patients’ mothers face challenges in caring for their babies when they must go back to work, did you have any problems?

a. Were you interested in continuing to breastfeed?

b. Did you have difficulty obtaining a pump?

c. Does your work provide an area to pump?

i. While it is likely too late to go back to breastfeeding these questions could be helpful to provide guidance if mother has future children.

B. Can you tell me a little about why you’re adding extra water to the formula?

a. Has anyone set you up with WIC access? Do you ever have difficulty paying for formula?

C. Do you have friends or family locally who are helping you out with the baby?

D. Did you face any challenges in making an appointment for your visit today?

a. Is finding transportation to doctors’ appointments difficult?

**Social Determinants of Health Case: Urgent Care (Faculty Supplement)**

A 16 y/o girl with a history of exposure to domestic violence presents to the ED with altered mental status. On further questioning she reports that she took a handful of pills she found in her grandmother’s medicine cabinet. She lives with her grandmother and 3 siblings. Per her grandmother the patient last saw her PCP for her “required middle school shots” a few years ago. She was seen in the ED four months ago for suicidal ideation. At that time, she was discharged with a safety plan and numbers for local psychologists.

**What social determinants of health might be a factor in this scenario?**

Potential SDHs:

1. Access to health care services
2. Time since PCP visit may be due to challenges with access and thus lack of routine depression/anxiety screenings
3. May have limited access to outpatient psychology due to waitlist times, insurance coverage, or difficulty scheduling appointments around parent/guardian work schedules

B. Transportation options

1. May have limited transportation options to access psychiatric services

C. Social support

1. If grandmother is primary guardian for patient and siblings, there may be limited social support for care of siblings at times of potential psychology appointments

D. Exposure to crime, violence, and social disorder

a. Patient has history of exposure to domestic violence

E. Socioeconomic conditions

1. Family may be able unable to afford pill box safe

**What kinds of questions could you ask the patient’s family to discover what social determinants of health might be affecting her?**

Potential SDH questions:

1. I see you were provided a list of resources from the ED for psychiatric services.

However, I know many of our families face challenges in scheduling or making it to these appointments. Did you encounter any of these or other challenges?

1. Have you faced challenges in the past in seeing your PCP?
2. Who in the area are you able to call when you need extra help or someone to talk to?
3. Do you feel safe at home?

**Social Determinants of Health Case: Clinical Problem Solving (Faculty Supplement)**

A 7 y/o boy with a history of epilepsy presents to the ED in status epilepticus. The status resolves after lorazepam and a levetiracetam load. On obtaining a history, you learn that the patient has not seen a Neurologist in a year and half. The family moved to the area 9 months ago for his mother’s job and have no family locally. He has a PCP appointment next month at which time she was hoping to get a referral to a local Neurologist. He ran out of his levetiracetam a week ago, and his mother has not been able to make it to the pharmacy to get a refill.

**What is your leading diagnosis?**

Breakthrough seizure secondary to missed medication

**What are some factors you think could be contributing to his health status?**

Potential SDH factors:

1. Availability of resources to meet daily needs
   1. Pharmacy may not be local
2. Access to health care services
   1. Patient may have faced barriers to PCP access as his appointment is not for another month
   2. Access to a new Neurologist may be limited by requirement for a PCP referral or by who accepts the patient’s insurance
3. Transportation options
   1. Family may rely upon public transportation which may serve as a barrier to timely pharmacy access
4. Social support
   1. Family has no extended family in the area

What additional questions would you like to ask the patient and his family?

Potential questions:

1. Where is your closest pharmacy?
   1. How do you get to that pharmacy?
2. Some of our families have difficulty getting appointments with PCPs or subspecialists like Neurology. Did you have any problems with this?
3. When you need extra help at home is there anyone in the local area you can call?

**Social Determinants of Health Case: Chronic Illness (Faculty Supplement)**

A 2 y/o boy with HbSS recently immigrated from the Democratic Republic of Congo (DRC) presents to the emergency department with fussiness. On physical exam he is awake but crying and has scleral icterus and splenomegaly. The remainder of the physical exam is unremarkable. He is afebrile. He was recently seen by a pediatrician and started on penicillin prophylaxis and referred to Hematology with whom he has an upcoming appointment. He is accompanied by his mother and aunt who offers to translate for the mother.

**What is your leading diagnosis?**

Splenic sequestration

**What are some factors you think could be contributing to his health status?**

Potential SDH:

1. Availability of resources to meet daily needs
   1. Medications
2. Access to health care services
   1. In the DRC and with transition to new country
3. Transportation options
   1. To the PCP, Hematologist, pharmacy
4. Social support
   1. Given recent immigration, may have limited social network
5. Exposure to crime, violence, and social disorder
   1. Unrest in the DRC may have driven immigration
6. Socioeconomic conditions
   1. Ability to afford medications, insurance coverage
7. Language/Literacy
   1. Language barrier
   2. Health literacy especially given complex regimen for sickle cell management

**What additional questions would you like to ask the patient’s family?**

Potential questions:

1. Were there any local clinics or other health care facilities where you lived before you moved here?
2. How do you get to your pediatrician’s office?
3. Have you had any difficulty in getting to see the Hematologist?
4. Have you found a local pharmacy?
5. What do you understand about your child’s sickle cell diagnosis?
6. Do you have anyone nearby you can call when problems arise or for extra help?
