# Supplementary material for: Social Determinants of Health Curriculum for the Pediatric Clerkship
Source: MedEdPORTAL. 2024 Oct 29;20:11458. doi: 10.15766/mep_2374-8265.11458 (PMC11518917; doi:10.15766/mep_2374-8265.11458)
Supplement: Supplementary file 1 — SDH Cases Faculty Supplements.docxCurriculum Orientation.pptxSDH Cases Student Handouts.docxPrework - Well Child.pptxPrework - Urgent Care.pptxPrework - Clinical Problem-solving.pptxPrework - Chronic Illness.pptxResource Assignment Orientation.pptxResource Assignment Form and Example.docxFacilitator Reminder Email.docxPresurvey and Case Analysis.docxPostsurvey and Case Analysis.docxCase Analysis Scoring Tool.docx [file mep_2374-8265.11458-s001.zip › B. Curriculum Orientation.pptx]

## Slide 1
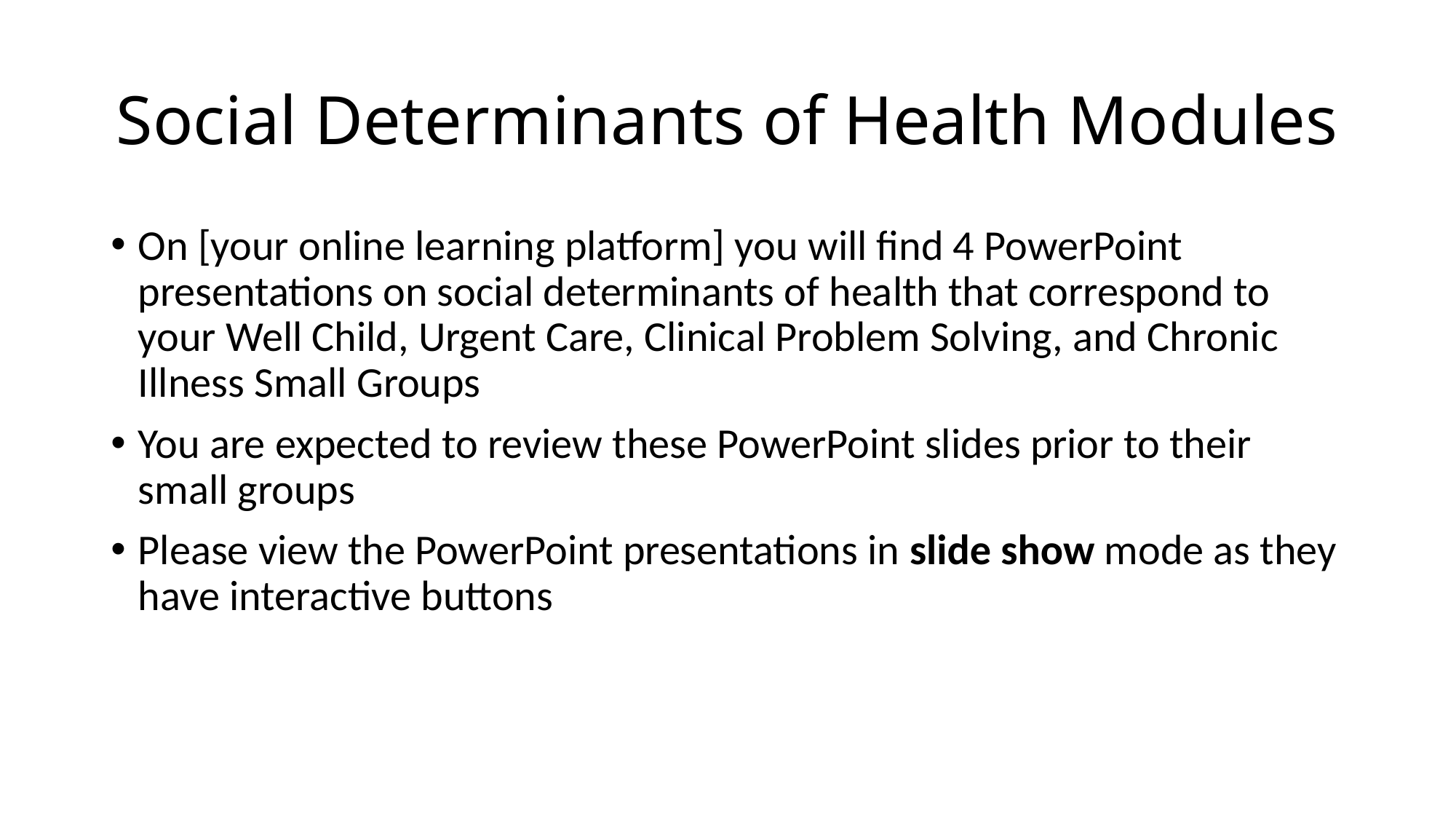

# Social Determinants of Health Modules
On [your online learning platform] you will find 4 PowerPoint presentations on social determinants of health that correspond to your Well Child, Urgent Care, Clinical Problem Solving, and Chronic Illness Small Groups
You are expected to review these PowerPoint slides prior to their small groups
Please view the PowerPoint presentations in slide show mode as they have interactive buttons
