# Supplementary material for: Social Determinants of Health Curriculum for the Pediatric Clerkship
Source: MedEdPORTAL. 2024 Oct 29;20:11458. doi: 10.15766/mep_2374-8265.11458 (PMC11518917; doi:10.15766/mep_2374-8265.11458)
Supplement: Supplementary file 1 — SDH Cases Faculty Supplements.docxCurriculum Orientation.pptxSDH Cases Student Handouts.docxPrework - Well Child.pptxPrework - Urgent Care.pptxPrework - Clinical Problem-solving.pptxPrework - Chronic Illness.pptxResource Assignment Orientation.pptxResource Assignment Form and Example.docxFacilitator Reminder Email.docxPresurvey and Case Analysis.docxPostsurvey and Case Analysis.docxCase Analysis Scoring Tool.docx [file mep_2374-8265.11458-s001.zip › C. SDH Cases Student Handouts.docx]

**Instructions for Use**: During small group sessions, each student should be provided a print copy of the case below that corresponds to that small group’s theme (e.g., well child). Students should participate in a 5–10 minute discussion of each case with facilitator guidance per Appendix A.

**Social Determinants of Health Case: Well Child**

A 3-month-old male infant comes to see his pediatrician for a well child visit. He has not been seen since his initial newborn visit. He has fallen from the 40^th^ to 25^th^ percentile for weight. His mother stopped breastfeeding a month ago when she went back to work. She reports mixing the formula 1 scoop to 2 oz but sometimes she adds extra water to make the formula go farther.

**What social determinants of health might be a factor in this scenario?**

**What kinds of questions could you ask the patient’s family to discover what social determinants of health might be affecting him?**

**Social Determinants of Health Case: Urgent Care**

A 16 y/o girl with a history of exposure to domestic violence presents to the ED with altered mental status. On further questioning she reports that she took a handful of pills she found in her grandmother’s medicine cabinet. She lives with her grandmother and 3 siblings. Per her grandmother the patient last saw her PCP for her “required middle school shots” a few years ago. She was seen in the ED four months ago for suicidal ideation. At that time, she was discharged with a safety plan and numbers for local psychologists.

**What social determinants of health might be a factor in this scenario?**

**What kinds of questions could you ask the patient’s family to discover what social determinants of health might be affecting her?**

**Social Determinants of Health Case: Clinical Problem Solving**

A 7 y/o boy with a history of epilepsy presents to the ED in status epilepticus. The status resolves after lorazepam and a levetiracetam load. On obtaining a history, you learn that the patient has not seen a Neurologist in a year and half. The family moved to the area 9 months ago for his mother’s job and have no family locally. He has a PCP appointment next month at which time she was hoping to get a referral to a local Neurologist. He ran out of his levetiracetam a week ago, and his mother has not been able to make it to the pharmacy to get a refill.

**What is your leading diagnosis?**

**What are some factors you think could be contributing to his health status?**

**What additional questions would you like to ask the patient and his family?**

**Social Determinants of Health Case: Chronic Illness**

A 2 y/o boy with HbSS recently immigrated from the Democratic Republic of Congo presents to the emergency department with fussiness. On physical exam he is awake but crying and has scleral icterus and splenomegaly. The remainder of the physical exam is unremarkable. He is afebrile. He was recently seen by a pediatrician and started on penicillin prophylaxis and referred to Hematology with whom he has an upcoming appointment. He is accompanied by his mother and aunt who offers to translate for the mother.

**What is your leading diagnosis?**

**What are some factors you think could be contributing to his health status?**

**What additional questions would you like to ask the patient’s family?**
