# Supplementary material for: Social Determinants of Health Curriculum for the Pediatric Clerkship
Source: MedEdPORTAL. 2024 Oct 29;20:11458. doi: 10.15766/mep_2374-8265.11458 (PMC11518917; doi:10.15766/mep_2374-8265.11458)
Supplement: Supplementary file 1 — SDH Cases Faculty Supplements.docxCurriculum Orientation.pptxSDH Cases Student Handouts.docxPrework - Well Child.pptxPrework - Urgent Care.pptxPrework - Clinical Problem-solving.pptxPrework - Chronic Illness.pptxResource Assignment Orientation.pptxResource Assignment Form and Example.docxFacilitator Reminder Email.docxPresurvey and Case Analysis.docxPostsurvey and Case Analysis.docxCase Analysis Scoring Tool.docx [file mep_2374-8265.11458-s001.zip › D. Prework - Well Child.pptx]

## Slide 1
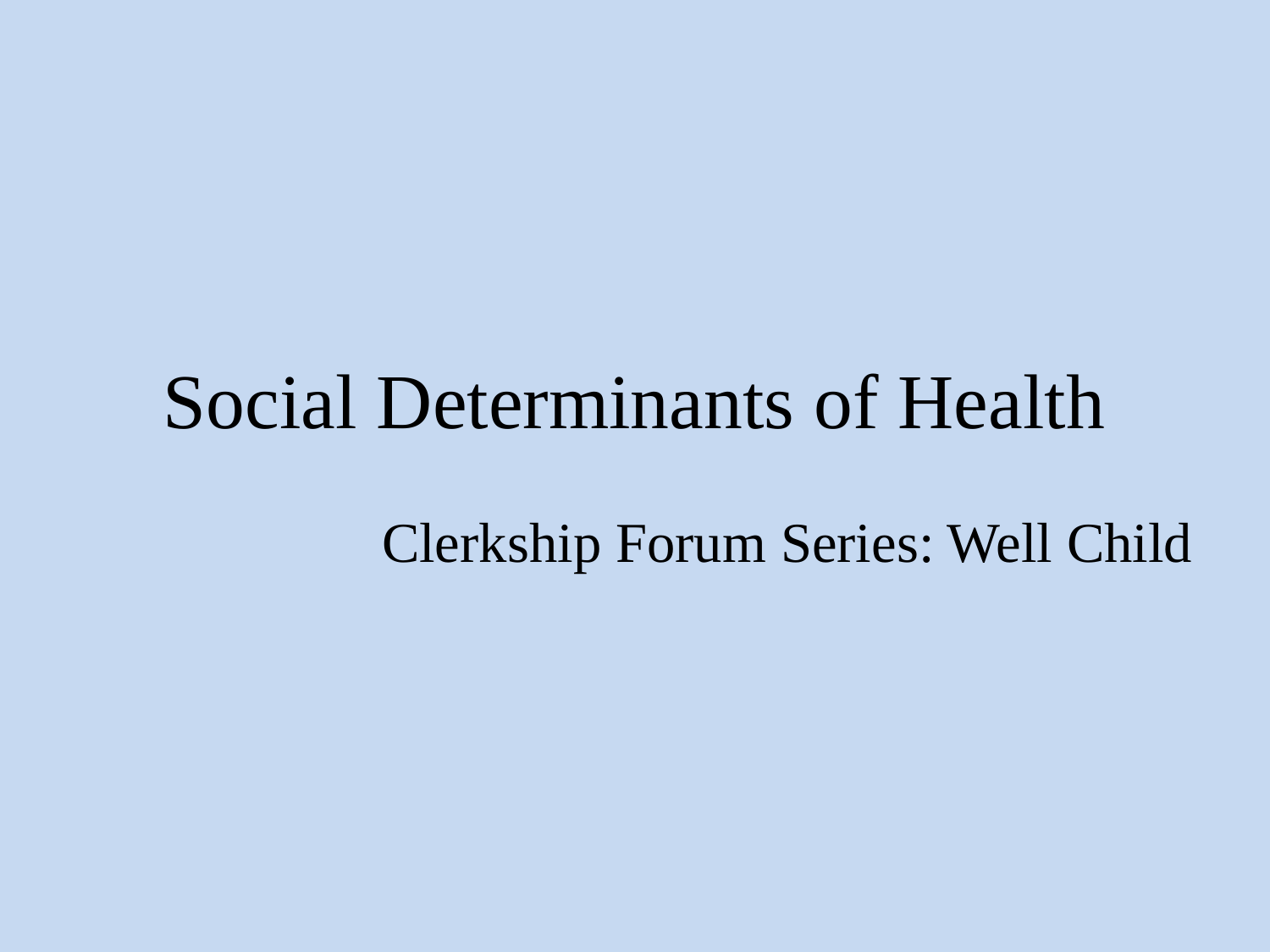

# Social Determinants of Health
Clerkship Forum Series: Well Child

## Slide 2
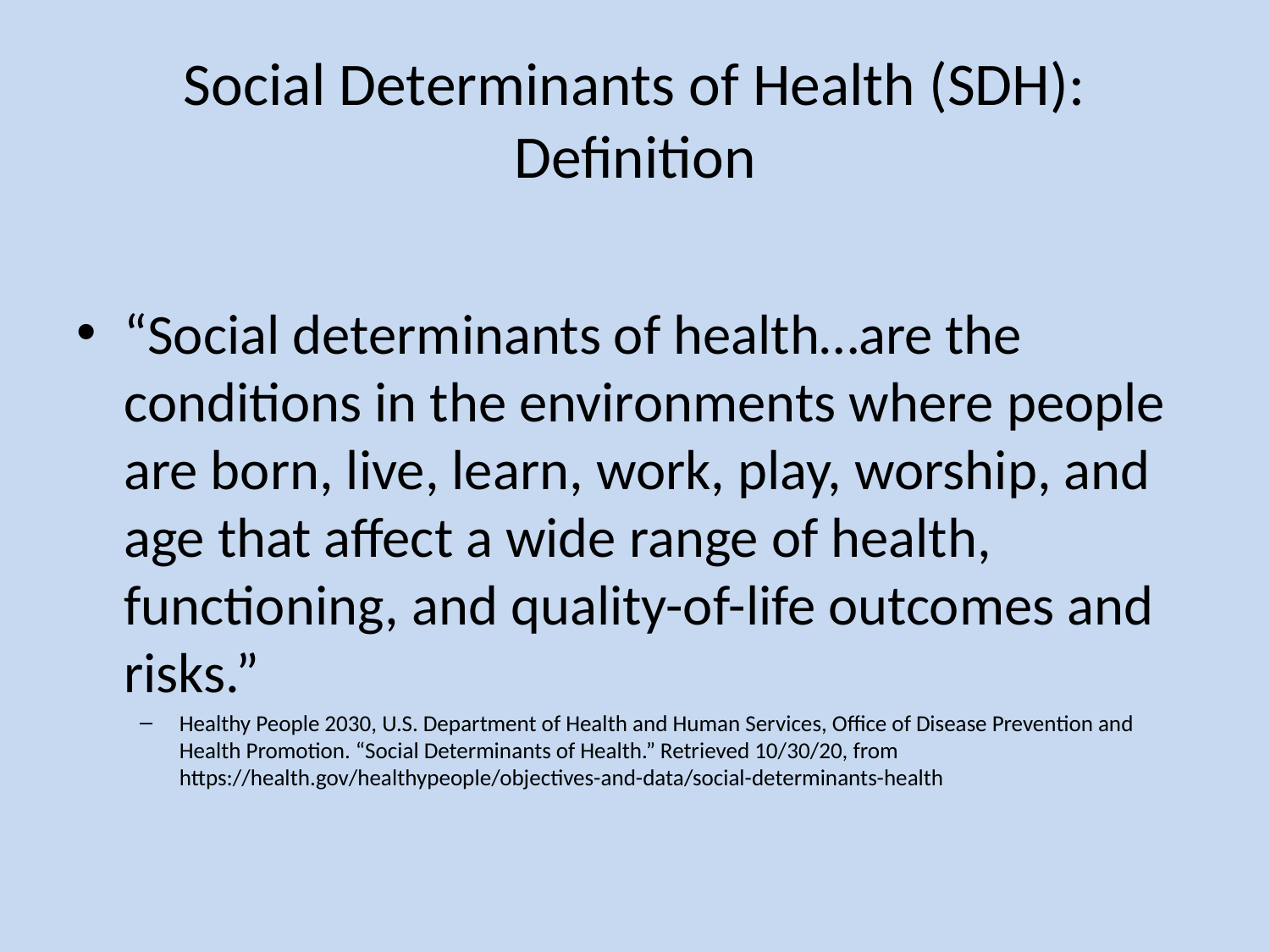

# Social Determinants of Health (SDH): Definition
“Social determinants of health…are the conditions in the environments where people are born, live, learn, work, play, worship, and age that affect a wide range of health, functioning, and quality-of-life outcomes and risks.”
Healthy People 2030, U.S. Department of Health and Human Services, Office of Disease Prevention and Health Promotion. “Social Determinants of Health.” Retrieved 10/30/20, from https://health.gov/healthypeople/objectives-and-data/social-determinants-health

## Slide 3
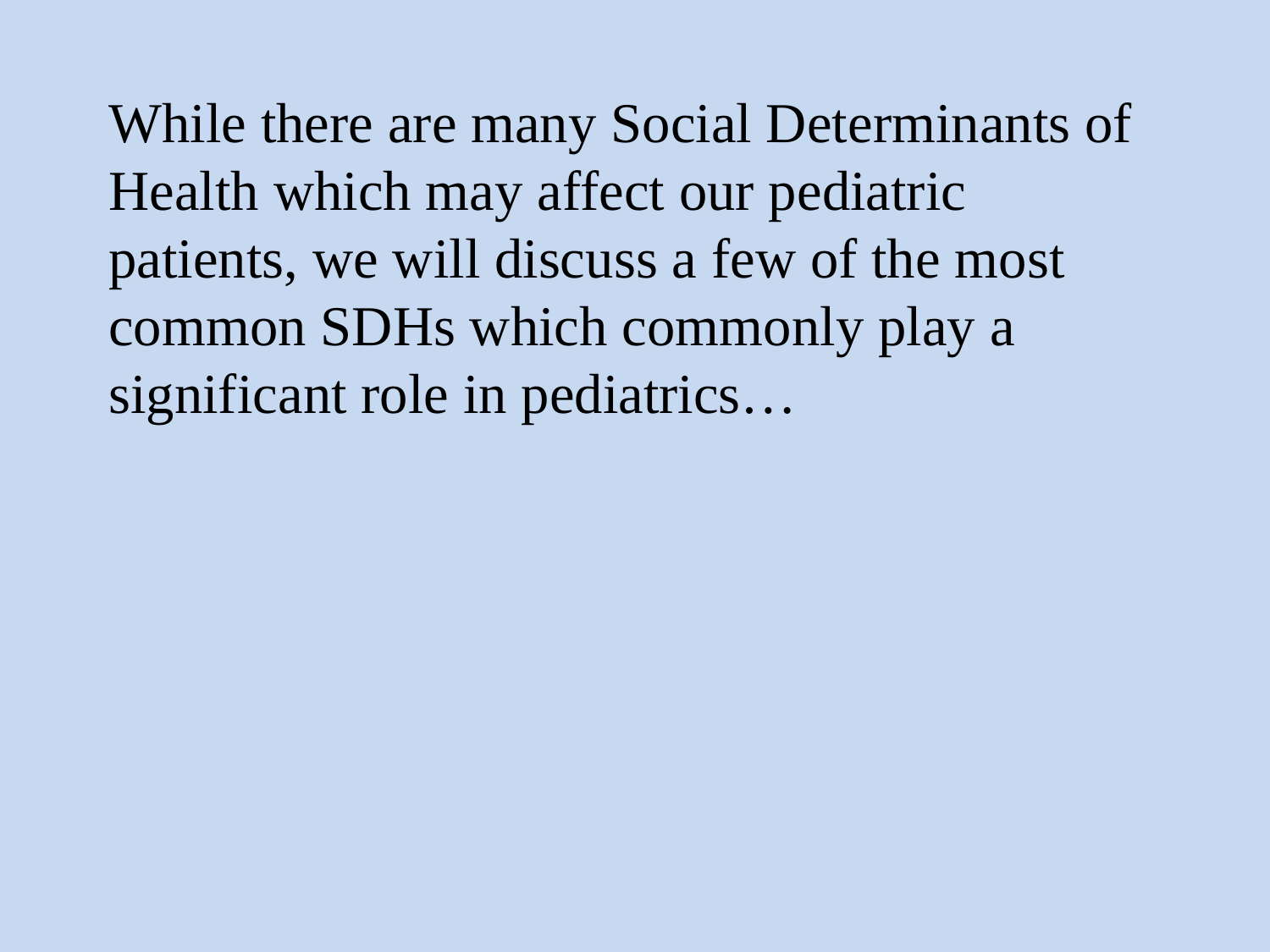

While there are many Social Determinants of Health which may affect our pediatric patients, we will discuss a few of the most common SDHs which commonly play a significant role in pediatrics…

## Slide 4
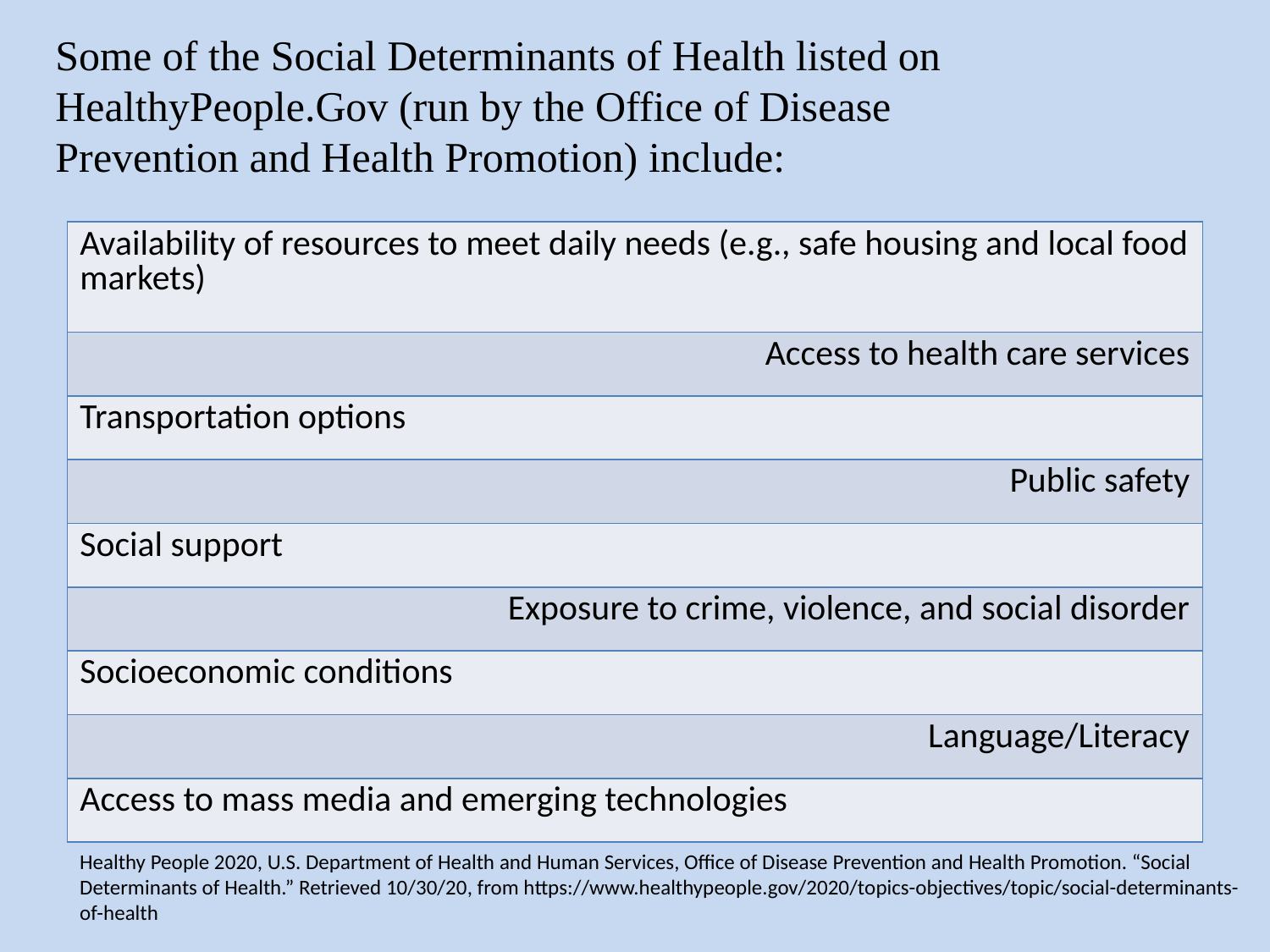

Some of the Social Determinants of Health listed on HealthyPeople.Gov (run by the Office of Disease Prevention and Health Promotion) include:
| Availability of resources to meet daily needs (e.g., safe housing and local food markets) |
| --- |
| Access to health care services |
| Transportation options |
| Public safety |
| Social support |
| Exposure to crime, violence, and social disorder |
| Socioeconomic conditions |
| Language/Literacy |
| Access to mass media and emerging technologies |
Healthy People 2020, U.S. Department of Health and Human Services, Office of Disease Prevention and Health Promotion. “Social Determinants of Health.” Retrieved 10/30/20, from https://www.healthypeople.gov/2020/topics-objectives/topic/social-determinants-of-health

## Slide 5
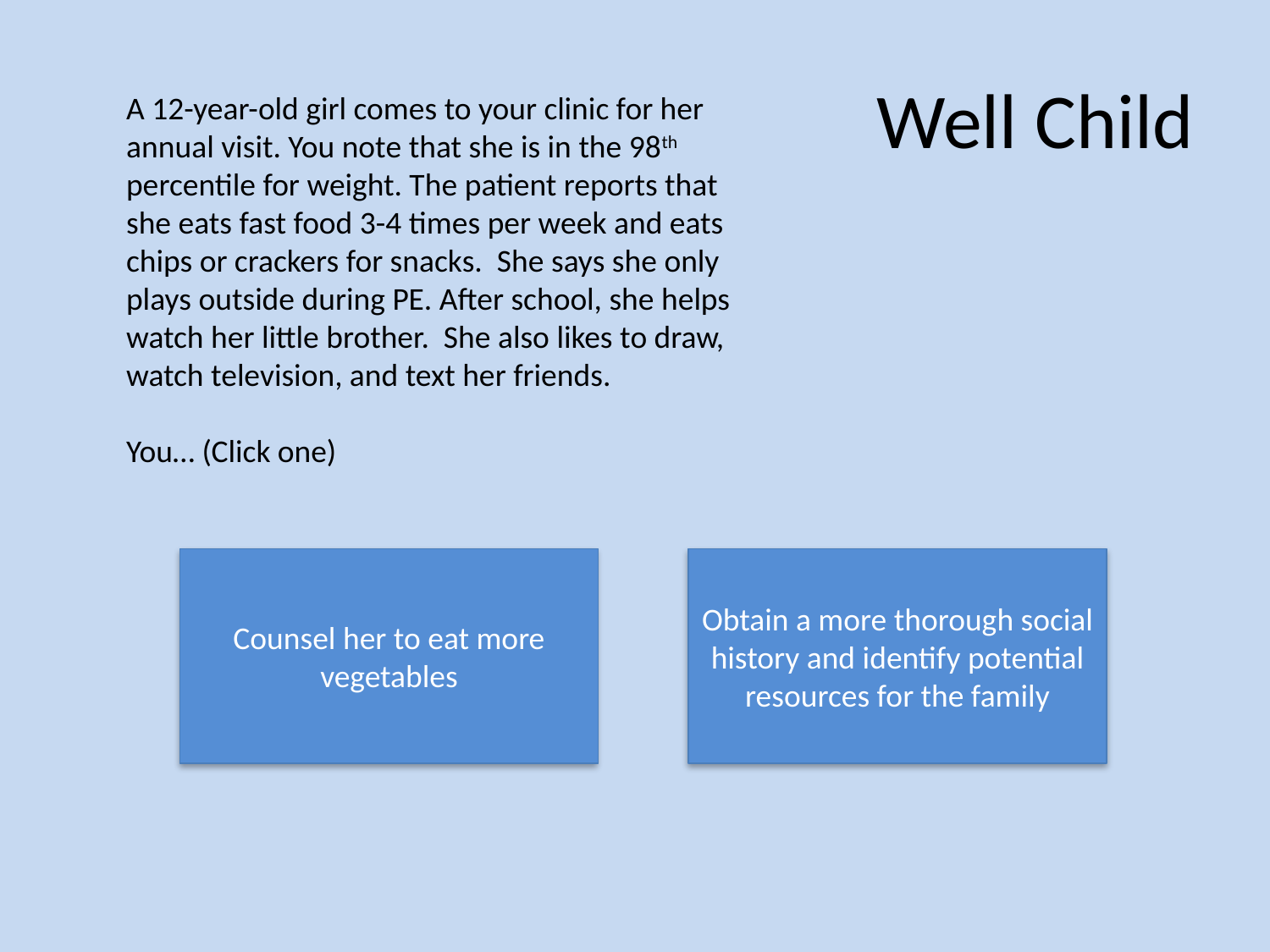

# Well Child
A 12-year-old girl comes to your clinic for her annual visit. You note that she is in the 98th percentile for weight. The patient reports that she eats fast food 3-4 times per week and eats chips or crackers for snacks. She says she only plays outside during PE. After school, she helps watch her little brother. She also likes to draw, watch television, and text her friends.
You… (Click one)
Counsel her to eat more vegetables
Obtain a more thorough social history and identify potential resources for the family

## Slide 6
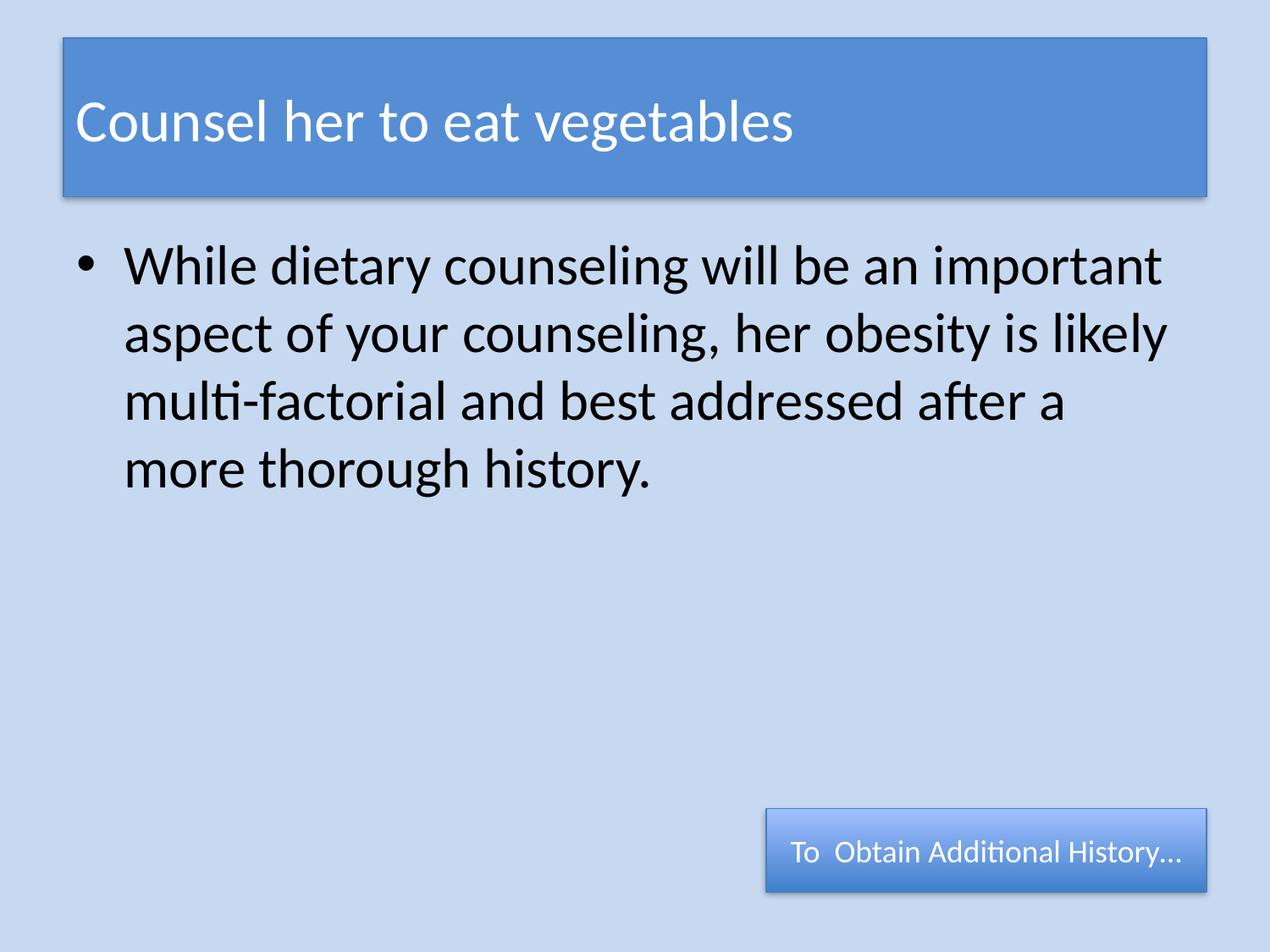

Counsel her to eat vegetables
While dietary counseling will be an important aspect of your counseling, her obesity is likely multi-factorial and best addressed after a more thorough history.
To Obtain Additional History…

## Slide 7
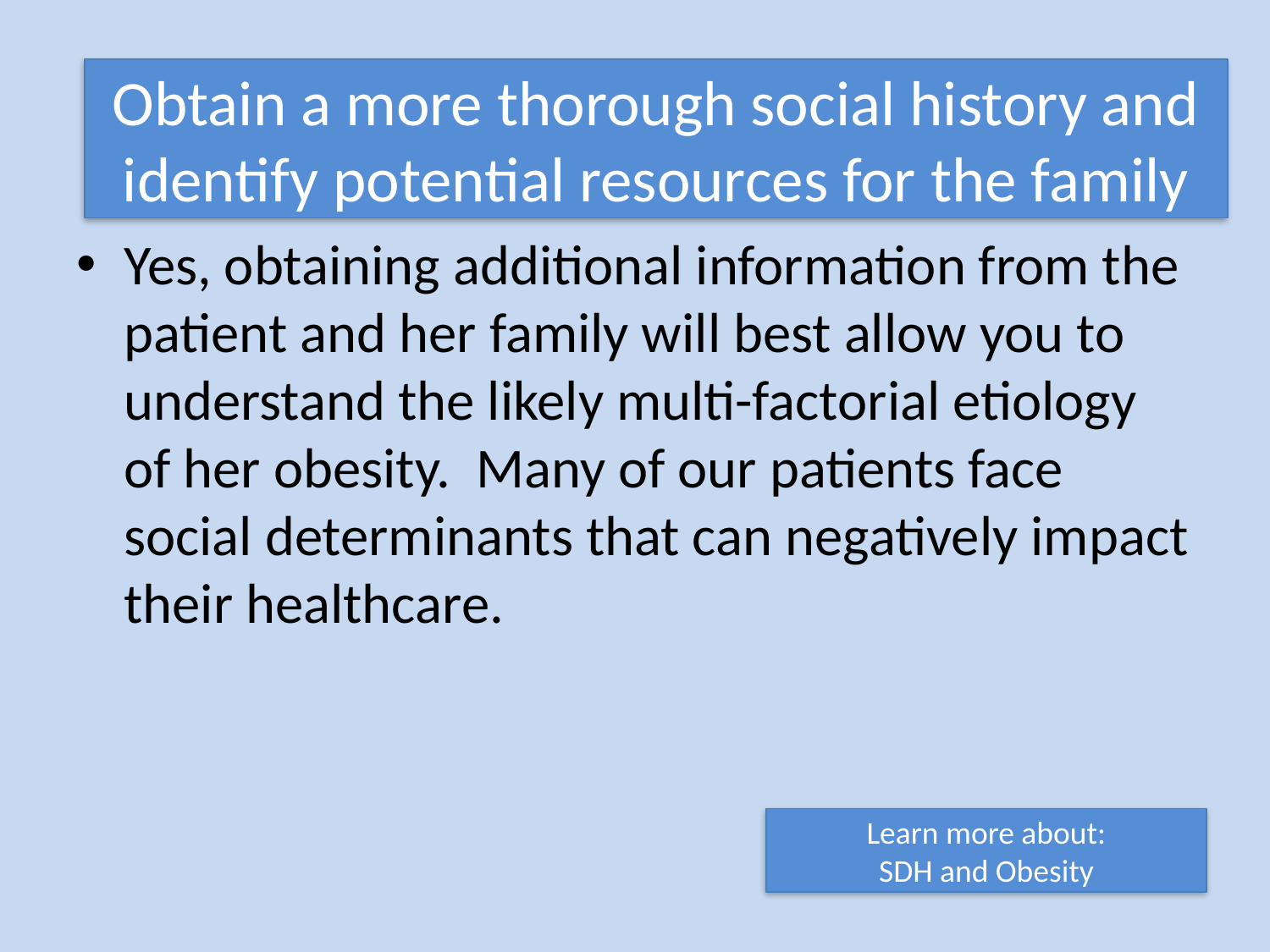

Obtain a more thorough social history and identify potential resources for the family
Yes, obtaining additional information from the patient and her family will best allow you to understand the likely multi-factorial etiology of her obesity. Many of our patients face social determinants that can negatively impact their healthcare.
Learn more about:
SDH and Obesity

## Slide 8
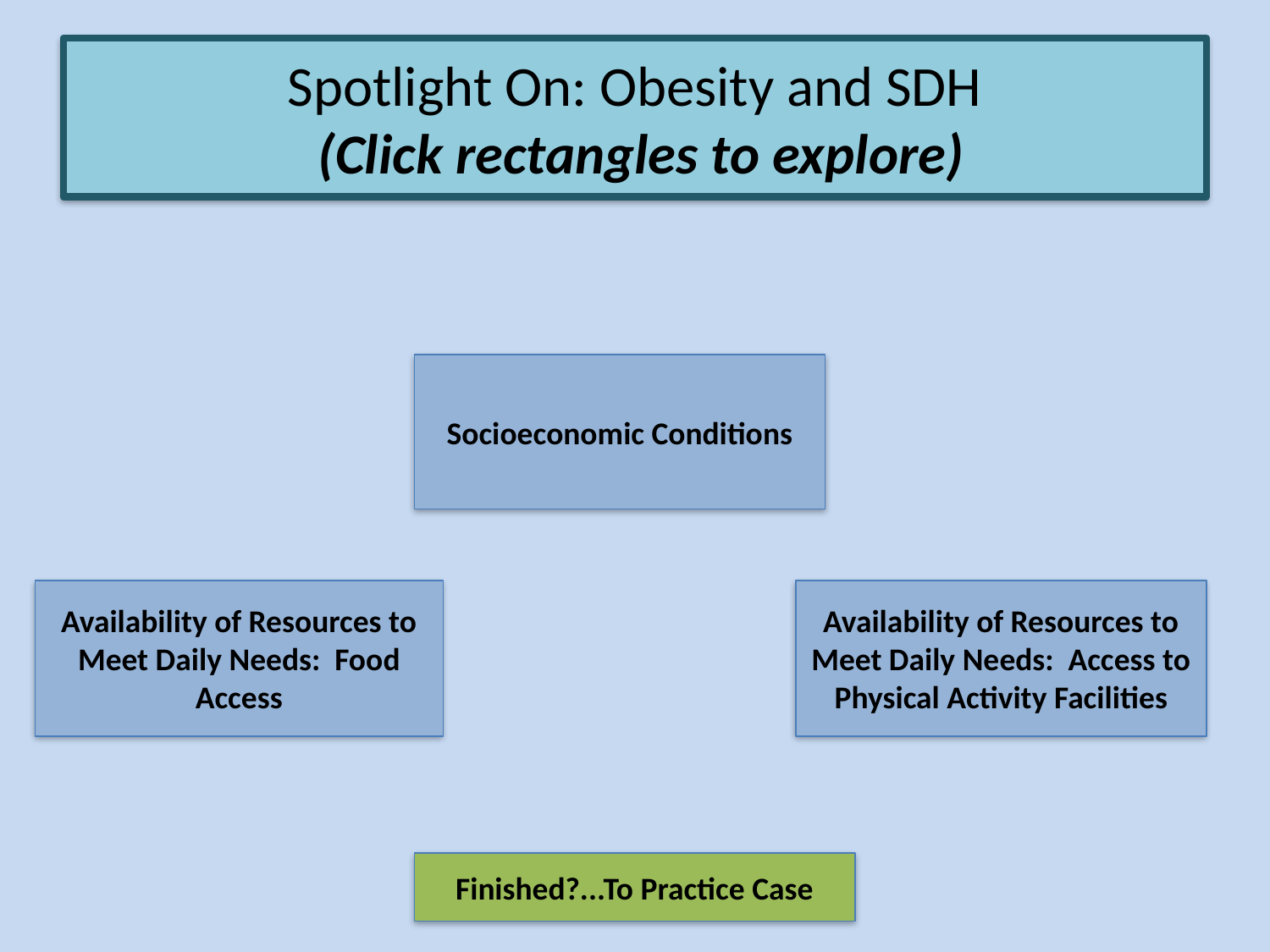

#
Spotlight On: Obesity and SDH
 (Click rectangles to explore)
Socioeconomic Conditions
Availability of Resources to Meet Daily Needs: Food Access
Availability of Resources to Meet Daily Needs: Access to Physical Activity Facilities
Finished?...To Practice Case

## Slide 9
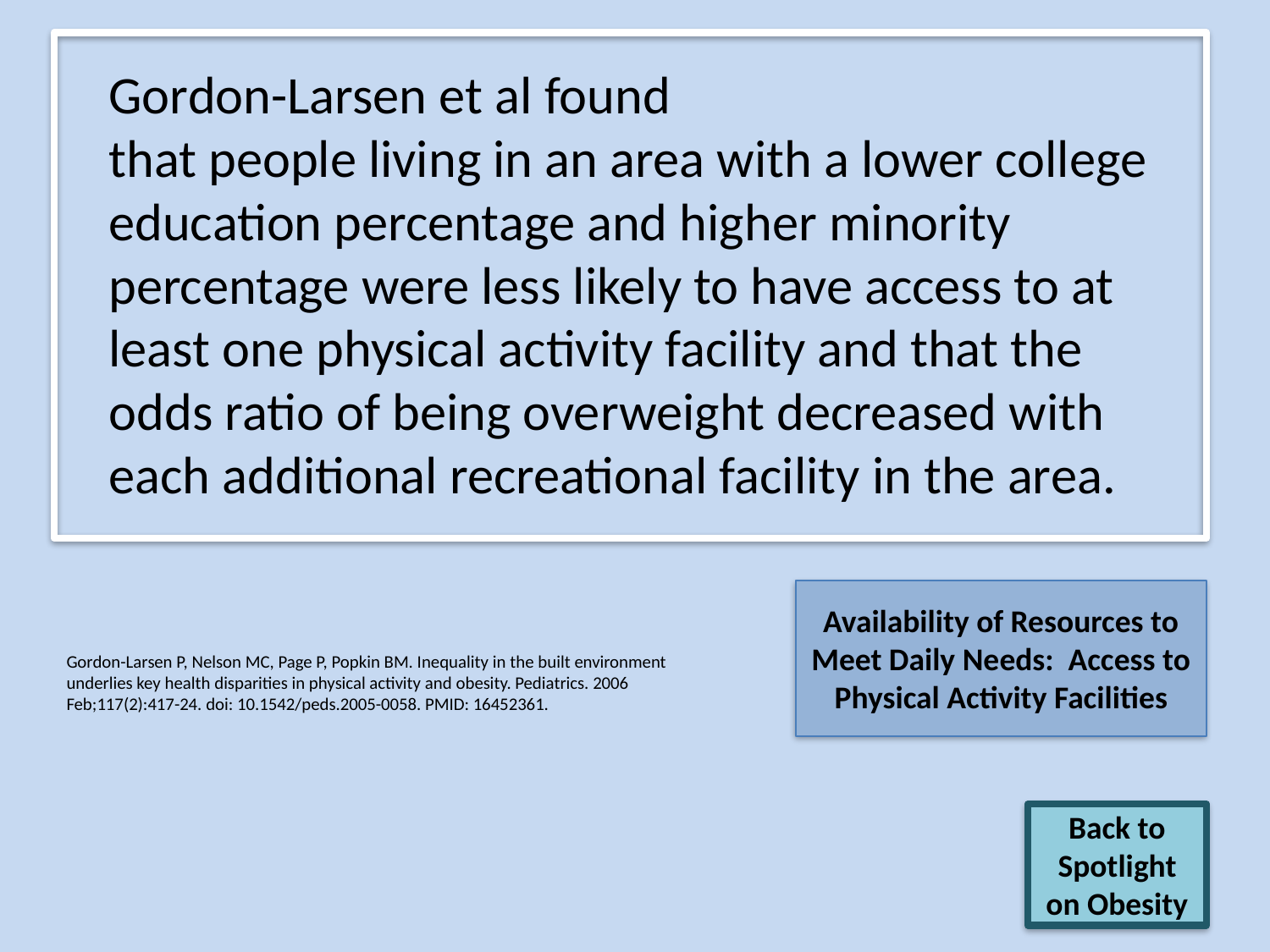

Gordon-Larsen et al found
that people living in an area with a lower college education percentage and higher minority percentage were less likely to have access to at least one physical activity facility and that the odds ratio of being overweight decreased with each additional recreational facility in the area.
Availability of Resources to Meet Daily Needs: Access to Physical Activity Facilities
Gordon-Larsen P, Nelson MC, Page P, Popkin BM. Inequality in the built environment underlies key health disparities in physical activity and obesity. Pediatrics. 2006 Feb;117(2):417-24. doi: 10.1542/peds.2005-0058. PMID: 16452361.
Back to Spotlight on Obesity

## Slide 10
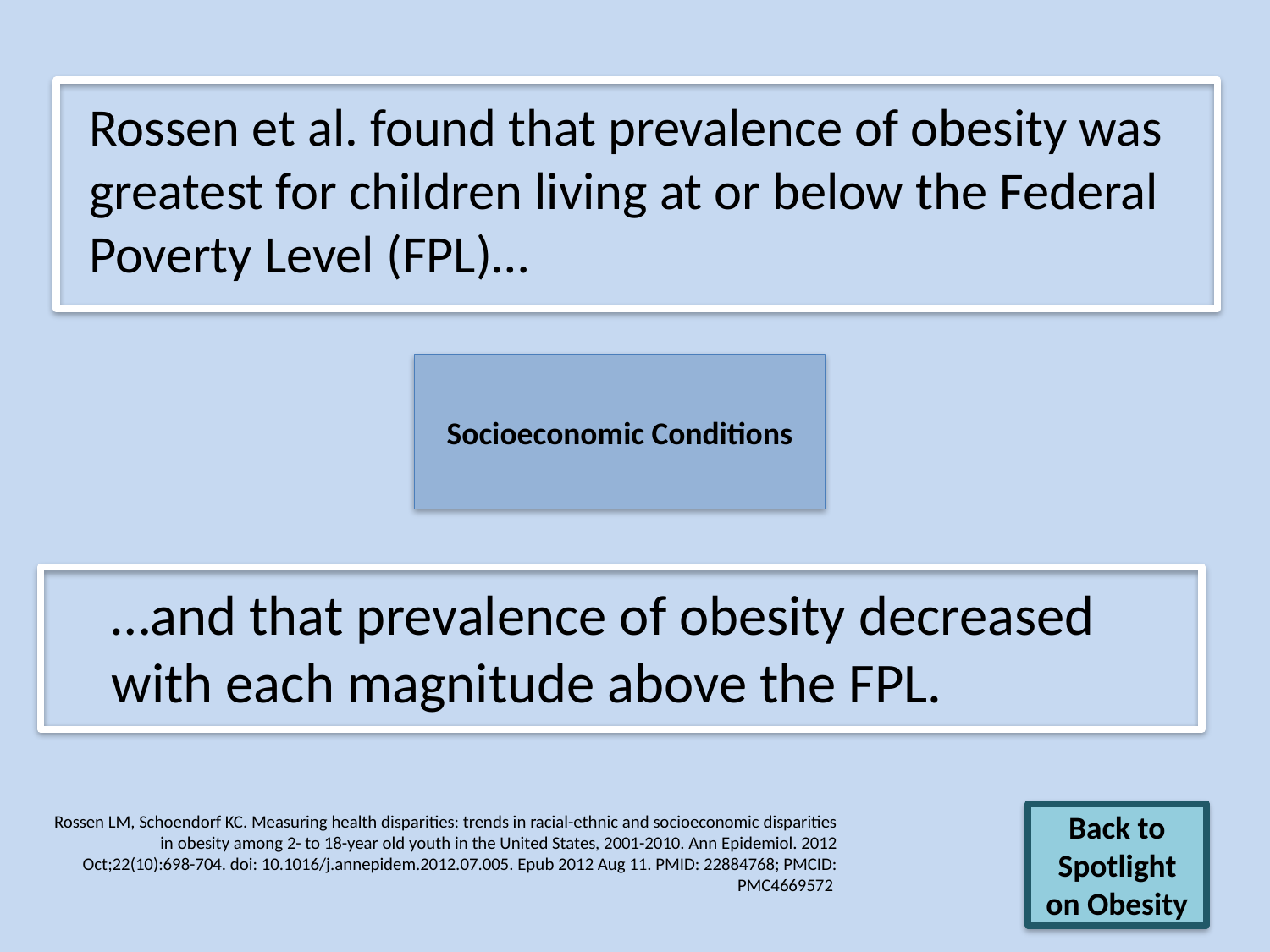

Rossen et al. found that prevalence of obesity was greatest for children living at or below the Federal Poverty Level (FPL)…
Socioeconomic Conditions
…and that prevalence of obesity decreased with each magnitude above the FPL.
Rossen LM, Schoendorf KC. Measuring health disparities: trends in racial-ethnic and socioeconomic disparities in obesity among 2- to 18-year old youth in the United States, 2001-2010. Ann Epidemiol. 2012 Oct;22(10):698-704. doi: 10.1016/j.annepidem.2012.07.005. Epub 2012 Aug 11. PMID: 22884768; PMCID: PMC4669572
Back to Spotlight on Obesity

## Slide 11
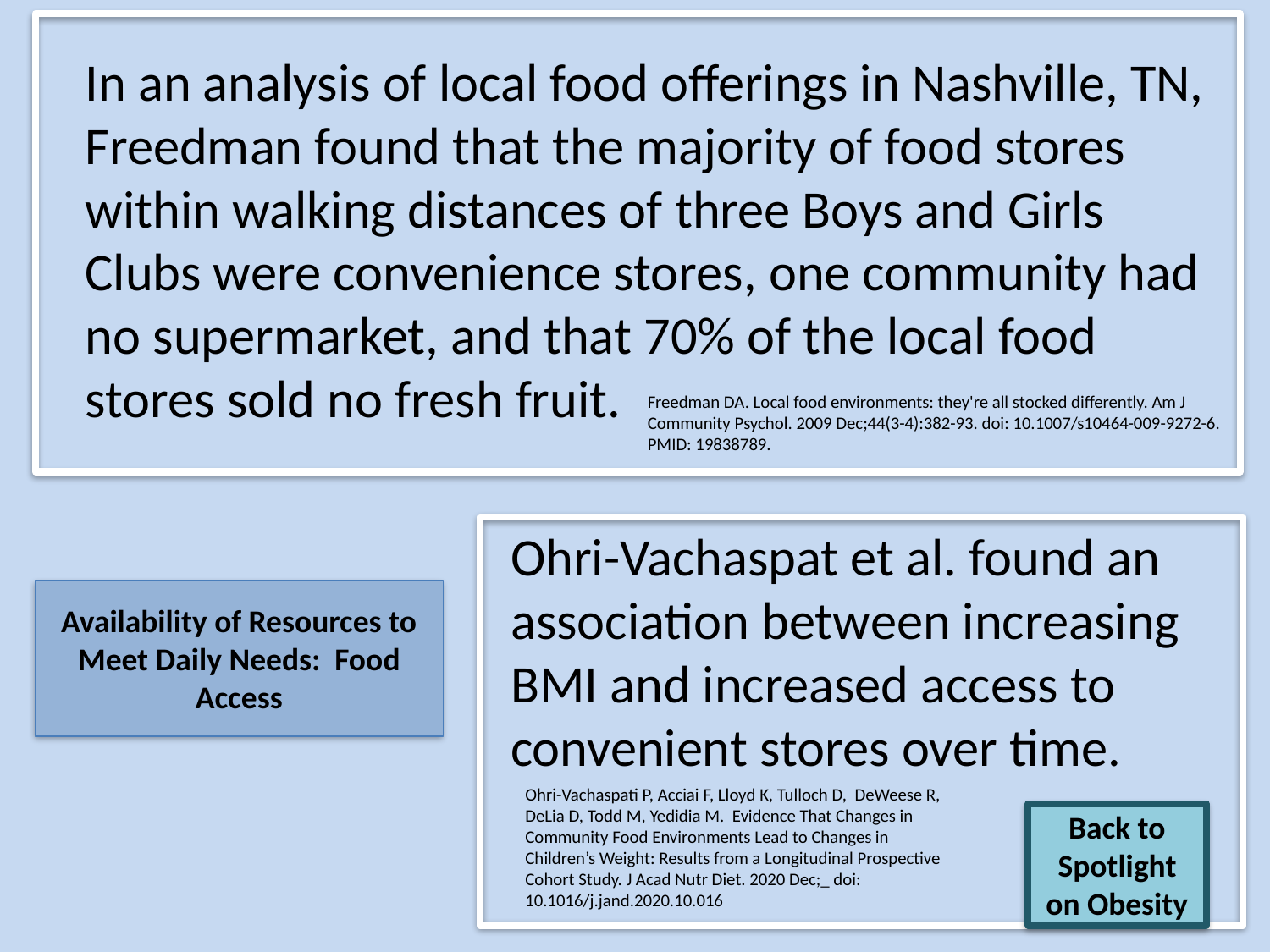

In an analysis of local food offerings in Nashville, TN, Freedman found that the majority of food stores within walking distances of three Boys and Girls Clubs were convenience stores, one community had no supermarket, and that 70% of the local food stores sold no fresh fruit.
Freedman DA. Local food environments: they're all stocked differently. Am J Community Psychol. 2009 Dec;44(3-4):382-93. doi: 10.1007/s10464-009-9272-6. PMID: 19838789.
Ohri-Vachaspat et al. found an association between increasing BMI and increased access to convenient stores over time.
Availability of Resources to Meet Daily Needs: Food Access
Ohri-Vachaspati P, Acciai F, Lloyd K, Tulloch D, DeWeese R, DeLia D, Todd M, Yedidia M. Evidence That Changes in Community Food Environments Lead to Changes in Children’s Weight: Results from a Longitudinal Prospective Cohort Study. J Acad Nutr Diet. 2020 Dec;_ doi: 10.1016/j.jand.2020.10.016
Back to Spotlight on Obesity

## Slide 12
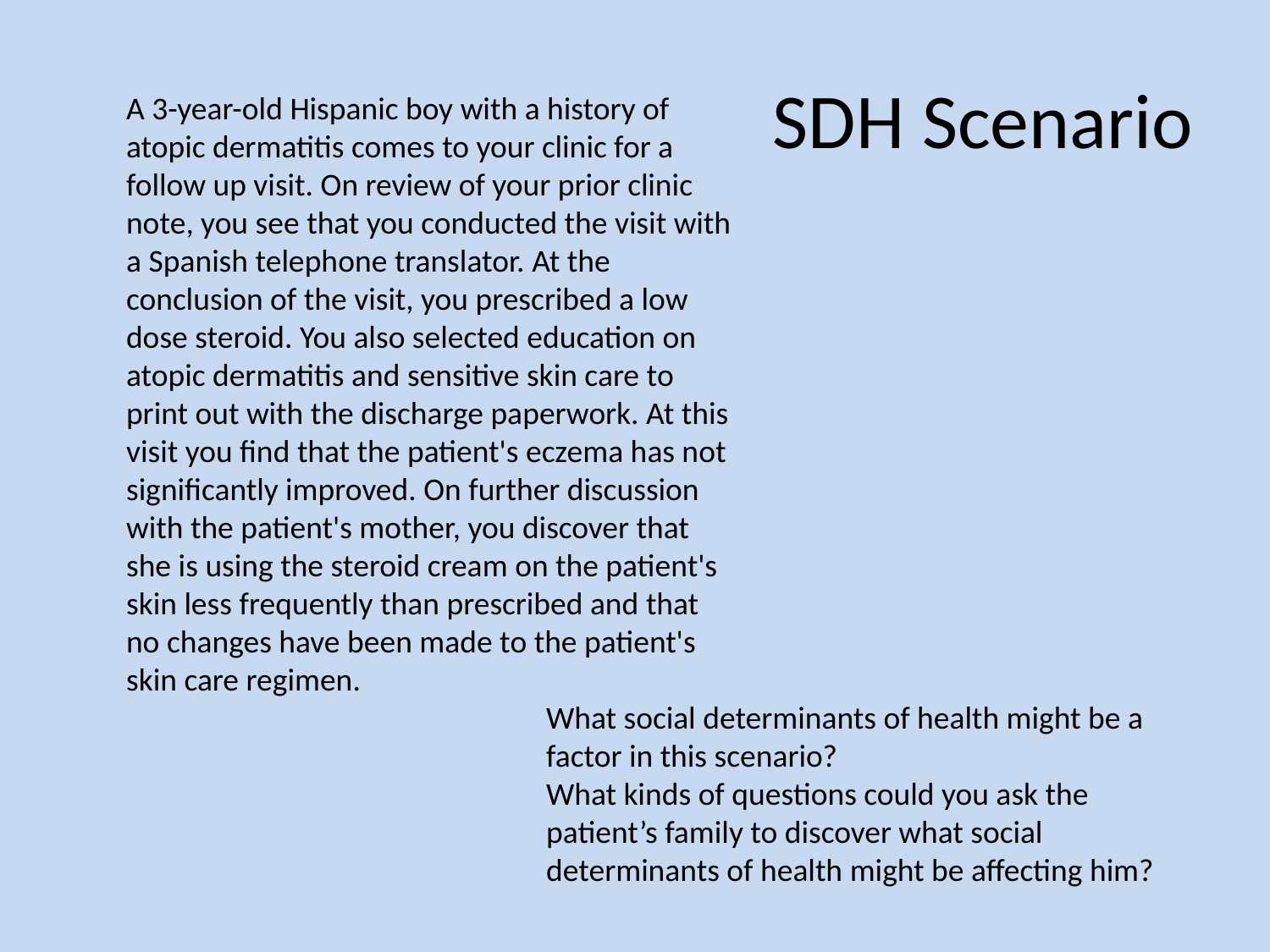

# SDH Scenario
A 3-year-old Hispanic boy with a history of atopic dermatitis comes to your clinic for a follow up visit. On review of your prior clinic note, you see that you conducted the visit with a Spanish telephone translator. At the conclusion of the visit, you prescribed a low dose steroid. You also selected education on atopic dermatitis and sensitive skin care to print out with the discharge paperwork. At this visit you find that the patient's eczema has not significantly improved. On further discussion with the patient's mother, you discover that she is using the steroid cream on the patient's skin less frequently than prescribed and that no changes have been made to the patient's skin care regimen.
What social determinants of health might be a factor in this scenario?
What kinds of questions could you ask the patient’s family to discover what social determinants of health might be affecting him?

## Slide 13
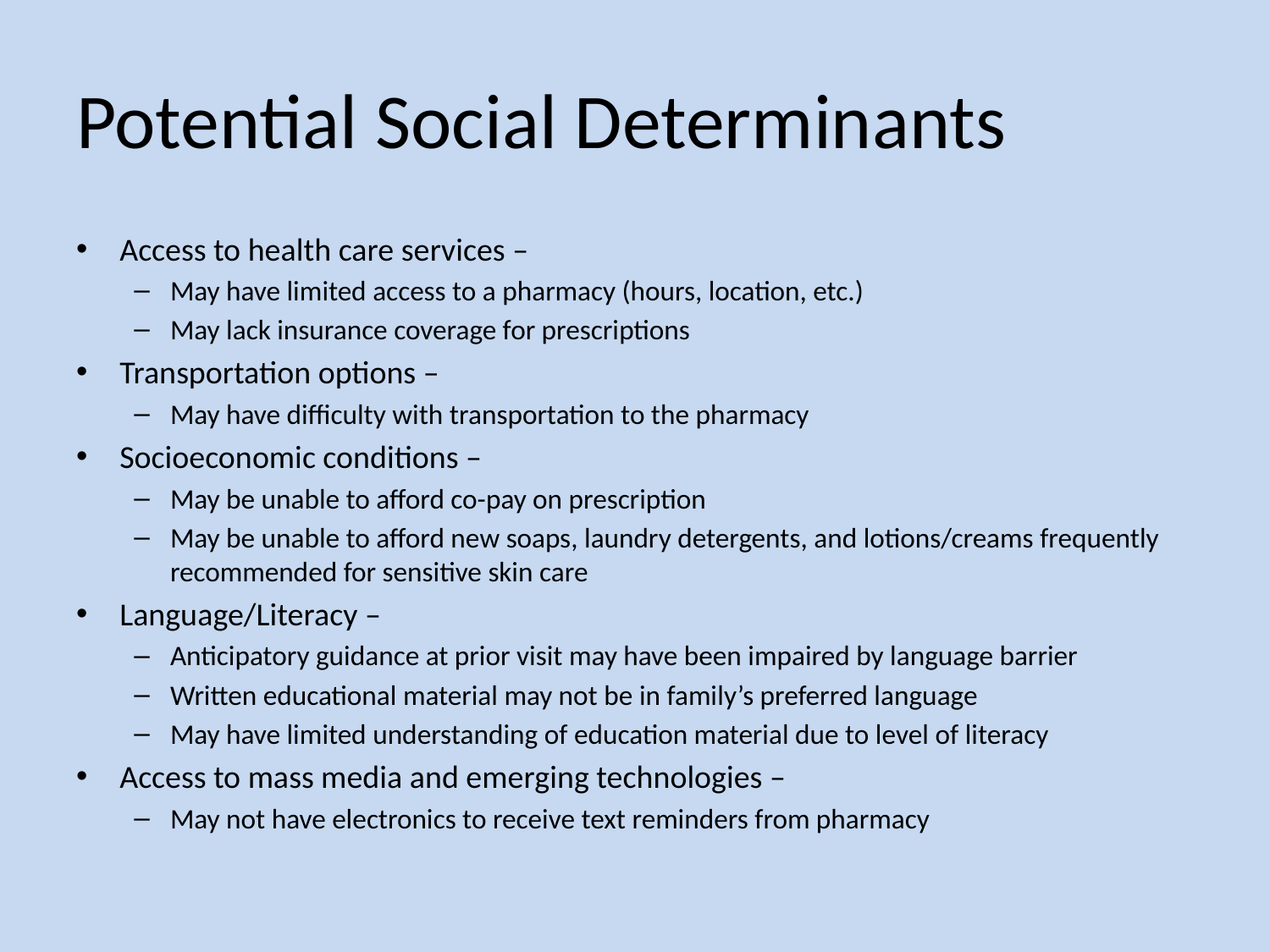

# Potential Social Determinants
Access to health care services –
May have limited access to a pharmacy (hours, location, etc.)
May lack insurance coverage for prescriptions
Transportation options –
May have difficulty with transportation to the pharmacy
Socioeconomic conditions –
May be unable to afford co-pay on prescription
May be unable to afford new soaps, laundry detergents, and lotions/creams frequently recommended for sensitive skin care
Language/Literacy –
Anticipatory guidance at prior visit may have been impaired by language barrier
Written educational material may not be in family’s preferred language
May have limited understanding of education material due to level of literacy
Access to mass media and emerging technologies –
May not have electronics to receive text reminders from pharmacy

## Slide 14
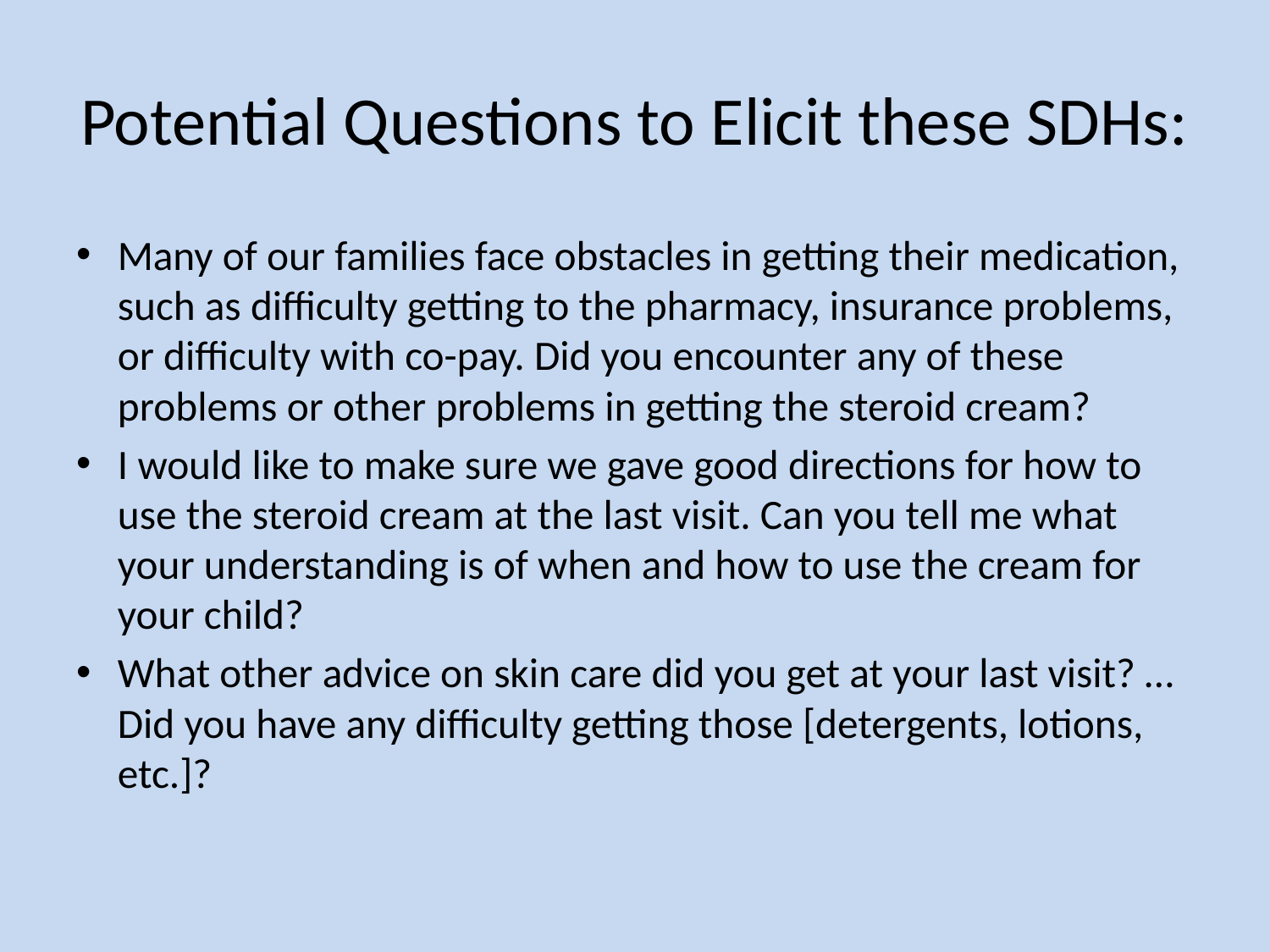

# Potential Questions to Elicit these SDHs:
Many of our families face obstacles in getting their medication, such as difficulty getting to the pharmacy, insurance problems, or difficulty with co-pay. Did you encounter any of these problems or other problems in getting the steroid cream?
I would like to make sure we gave good directions for how to use the steroid cream at the last visit. Can you tell me what your understanding is of when and how to use the cream for your child?
What other advice on skin care did you get at your last visit? … Did you have any difficulty getting those [detergents, lotions, etc.]?

## Slide 15
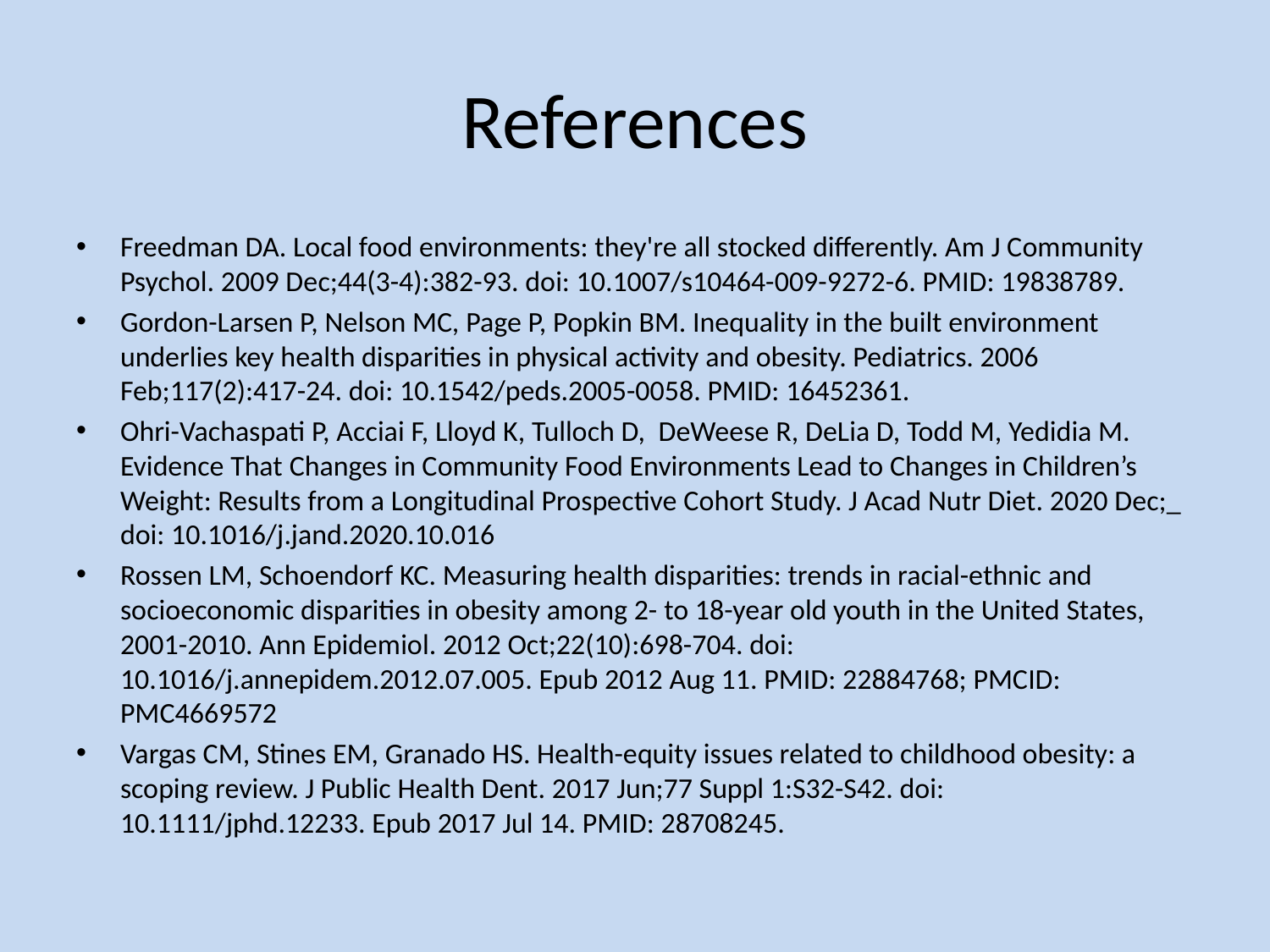

# References
Freedman DA. Local food environments: they're all stocked differently. Am J Community Psychol. 2009 Dec;44(3-4):382-93. doi: 10.1007/s10464-009-9272-6. PMID: 19838789.
Gordon-Larsen P, Nelson MC, Page P, Popkin BM. Inequality in the built environment underlies key health disparities in physical activity and obesity. Pediatrics. 2006 Feb;117(2):417-24. doi: 10.1542/peds.2005-0058. PMID: 16452361.
Ohri-Vachaspati P, Acciai F, Lloyd K, Tulloch D, DeWeese R, DeLia D, Todd M, Yedidia M. Evidence That Changes in Community Food Environments Lead to Changes in Children’s Weight: Results from a Longitudinal Prospective Cohort Study. J Acad Nutr Diet. 2020 Dec;_ doi: 10.1016/j.jand.2020.10.016
Rossen LM, Schoendorf KC. Measuring health disparities: trends in racial-ethnic and socioeconomic disparities in obesity among 2- to 18-year old youth in the United States, 2001-2010. Ann Epidemiol. 2012 Oct;22(10):698-704. doi: 10.1016/j.annepidem.2012.07.005. Epub 2012 Aug 11. PMID: 22884768; PMCID: PMC4669572
Vargas CM, Stines EM, Granado HS. Health-equity issues related to childhood obesity: a scoping review. J Public Health Dent. 2017 Jun;77 Suppl 1:S32-S42. doi: 10.1111/jphd.12233. Epub 2017 Jul 14. PMID: 28708245.
