# Supplementary material for: Social Determinants of Health Curriculum for the Pediatric Clerkship
Source: MedEdPORTAL. 2024 Oct 29;20:11458. doi: 10.15766/mep_2374-8265.11458 (PMC11518917; doi:10.15766/mep_2374-8265.11458)
Supplement: Supplementary file 1 — SDH Cases Faculty Supplements.docxCurriculum Orientation.pptxSDH Cases Student Handouts.docxPrework - Well Child.pptxPrework - Urgent Care.pptxPrework - Clinical Problem-solving.pptxPrework - Chronic Illness.pptxResource Assignment Orientation.pptxResource Assignment Form and Example.docxFacilitator Reminder Email.docxPresurvey and Case Analysis.docxPostsurvey and Case Analysis.docxCase Analysis Scoring Tool.docx [file mep_2374-8265.11458-s001.zip › E. Prework - Urgent Care.pptx]

## Slide 1
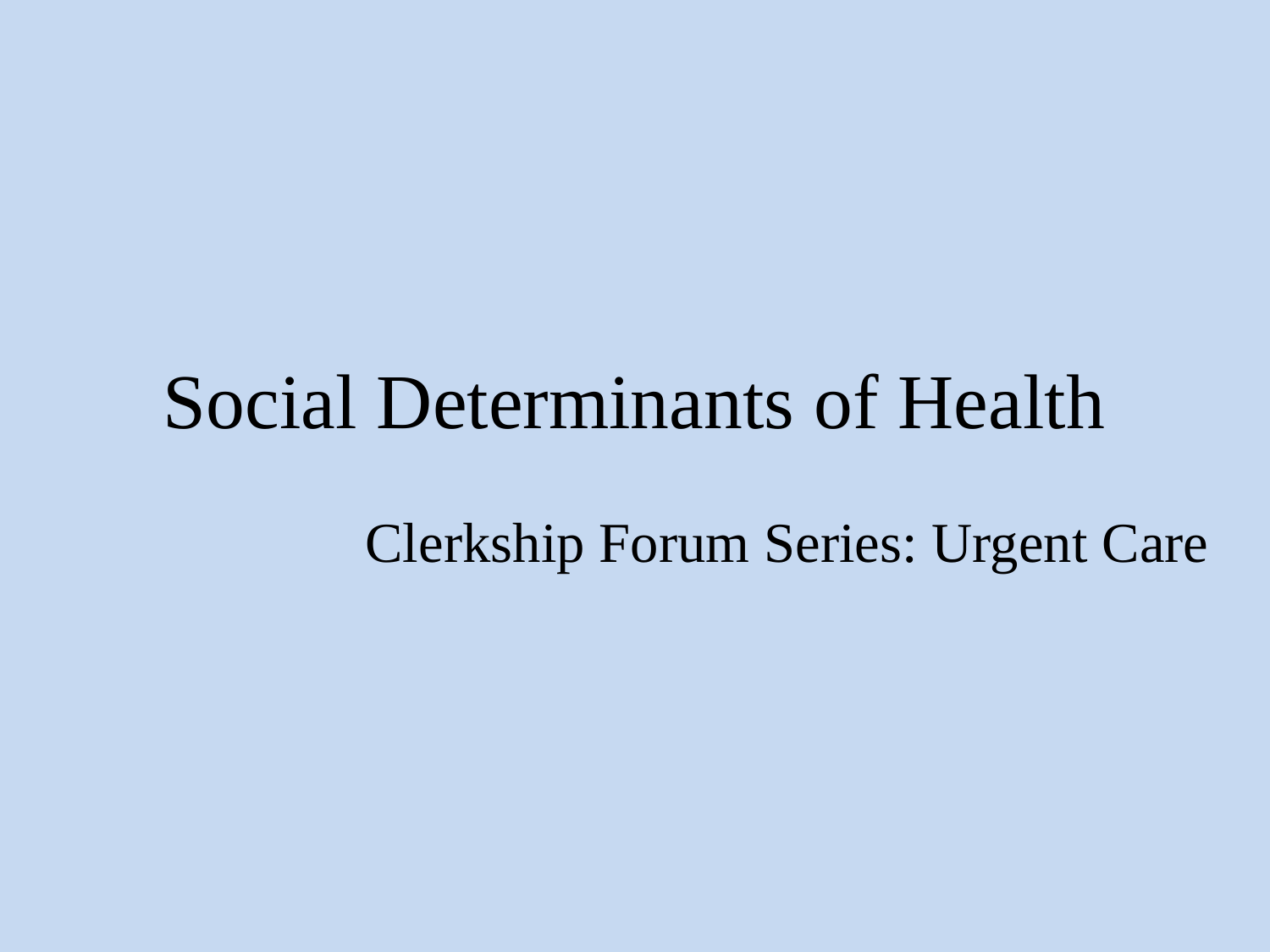

# Social Determinants of Health
Clerkship Forum Series: Urgent Care

## Slide 2
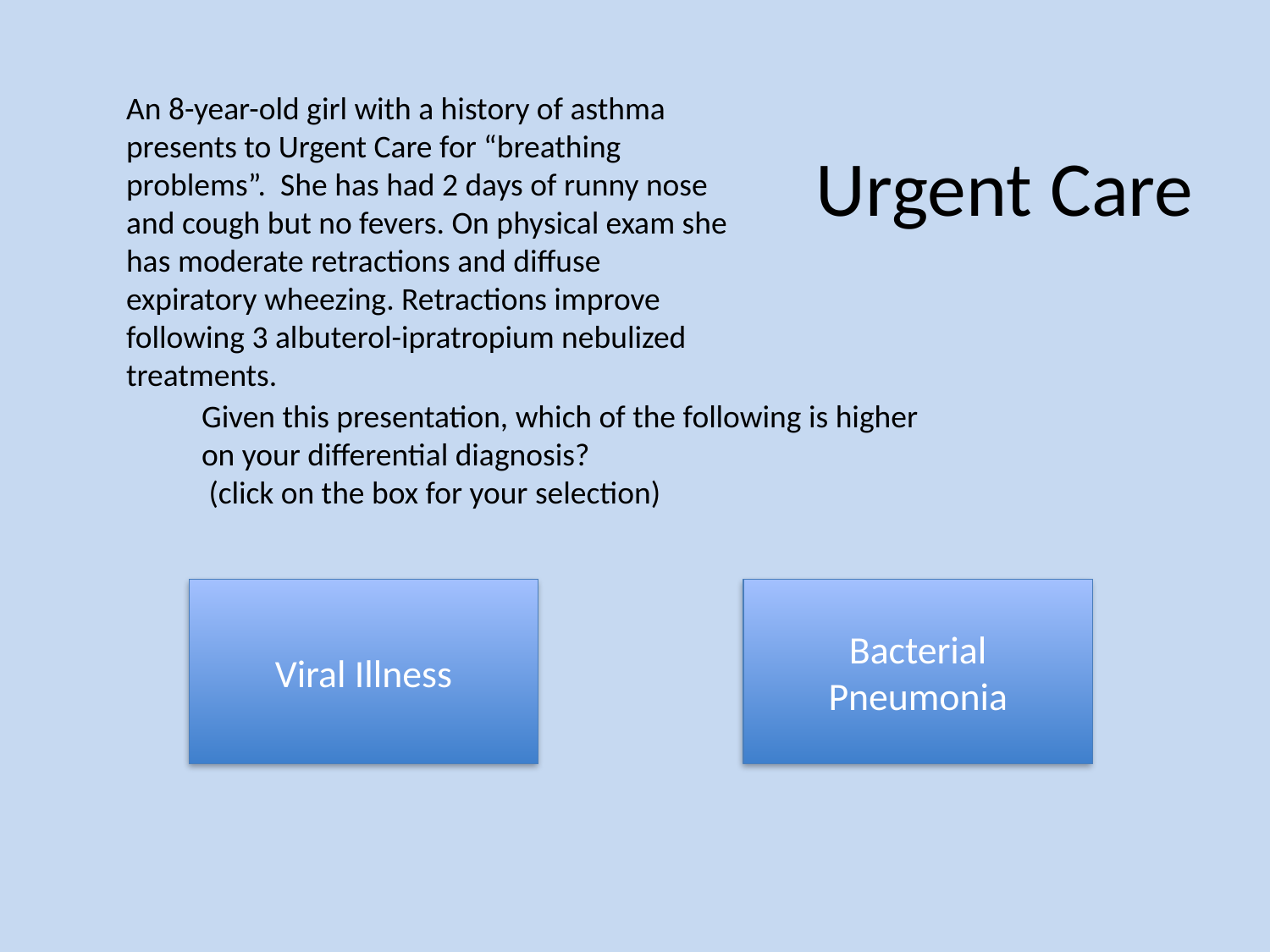

# Urgent Care
An 8-year-old girl with a history of asthma presents to Urgent Care for “breathing problems”. She has had 2 days of runny nose and cough but no fevers. On physical exam she has moderate retractions and diffuse expiratory wheezing. Retractions improve following 3 albuterol-ipratropium nebulized treatments.
Given this presentation, which of the following is higher on your differential diagnosis?
 (click on the box for your selection)
Viral Illness
Bacterial Pneumonia

## Slide 3
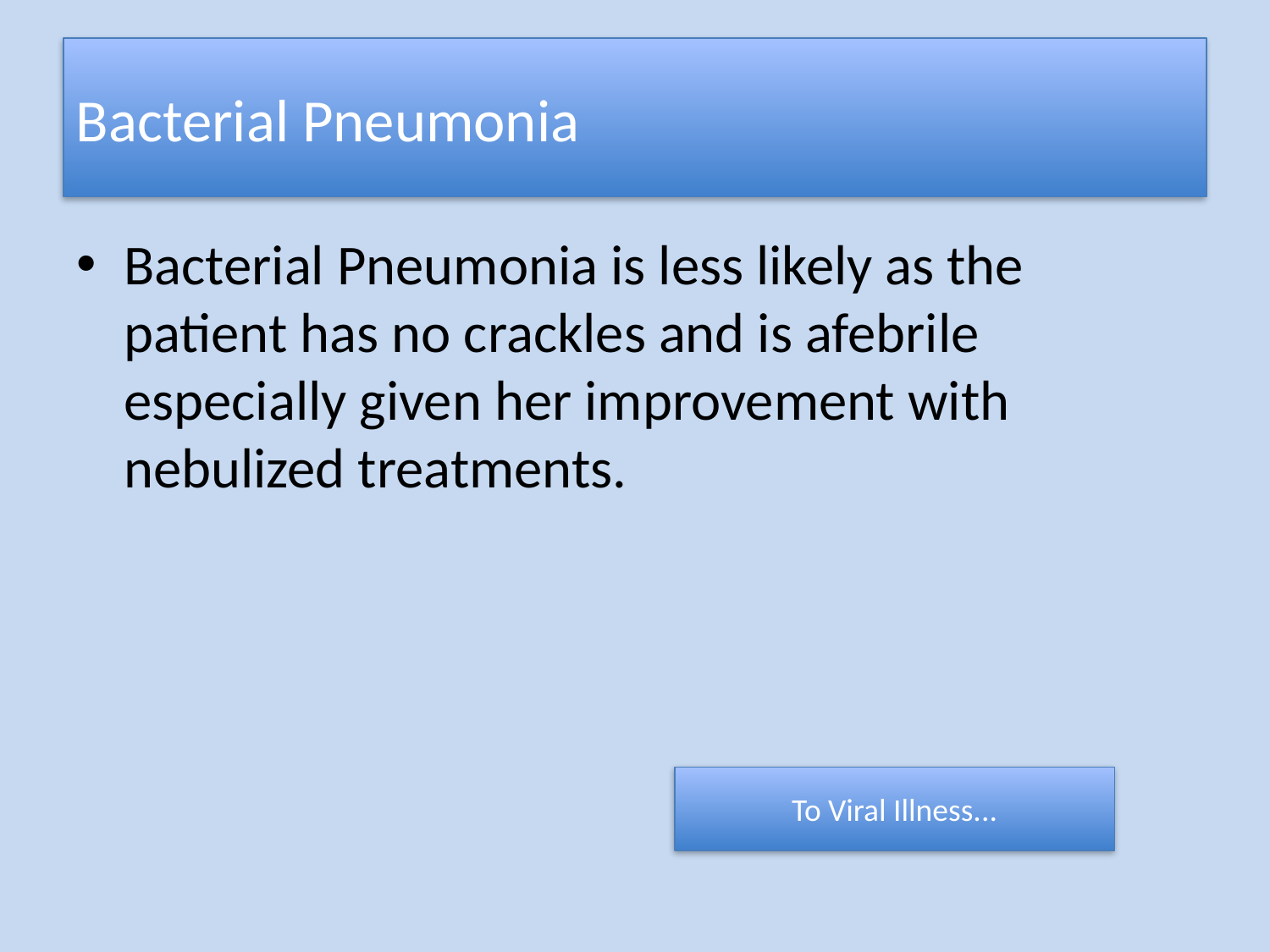

Bacterial Pneumonia
Bacterial Pneumonia is less likely as the patient has no crackles and is afebrile especially given her improvement with nebulized treatments.
To Viral Illness...

## Slide 4
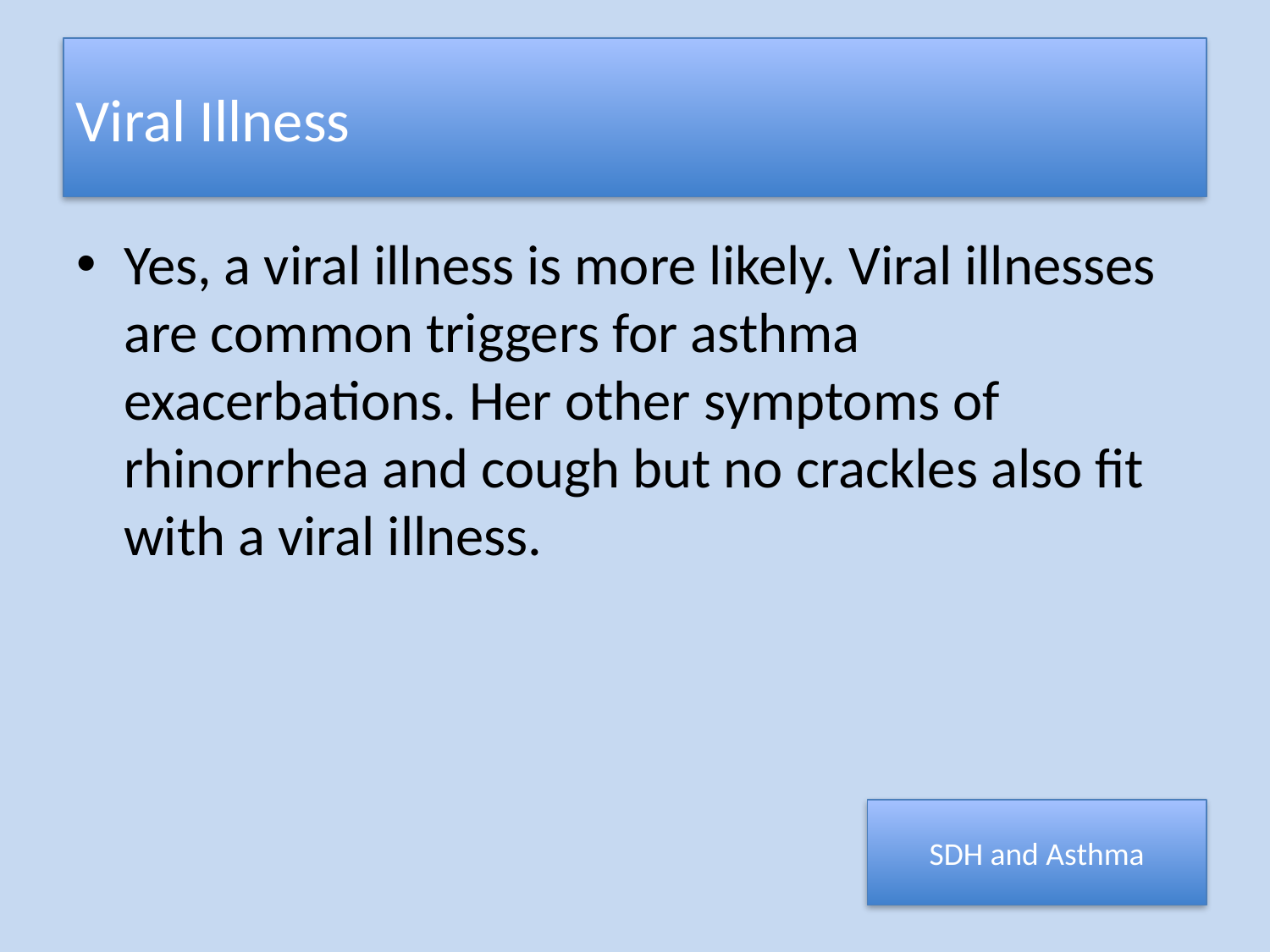

Viral Illness
Yes, a viral illness is more likely. Viral illnesses are common triggers for asthma exacerbations. Her other symptoms of rhinorrhea and cough but no crackles also fit with a viral illness.
SDH and Asthma

## Slide 5
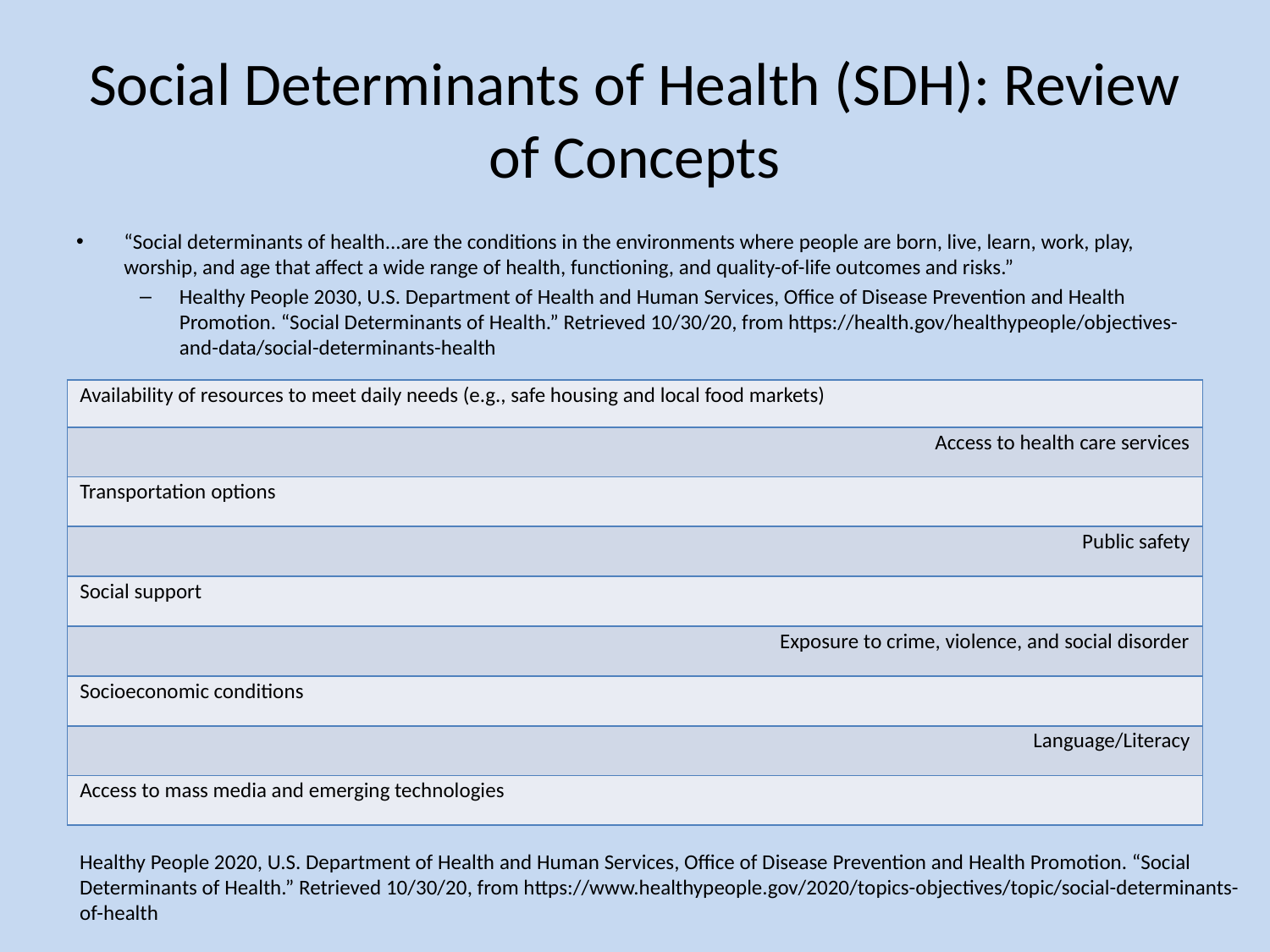

# Social Determinants of Health (SDH): Review of Concepts
“Social determinants of health...are the conditions in the environments where people are born, live, learn, work, play, worship, and age that affect a wide range of health, functioning, and quality-of-life outcomes and risks.”
Healthy People 2030, U.S. Department of Health and Human Services, Office of Disease Prevention and Health Promotion. “Social Determinants of Health.” Retrieved 10/30/20, from https://health.gov/healthypeople/objectives-and-data/social-determinants-health
| Availability of resources to meet daily needs (e.g., safe housing and local food markets) |
| --- |
| Access to health care services |
| Transportation options |
| Public safety |
| Social support |
| Exposure to crime, violence, and social disorder |
| Socioeconomic conditions |
| Language/Literacy |
| Access to mass media and emerging technologies |
Healthy People 2020, U.S. Department of Health and Human Services, Office of Disease Prevention and Health Promotion. “Social Determinants of Health.” Retrieved 10/30/20, from https://www.healthypeople.gov/2020/topics-objectives/topic/social-determinants-of-health

## Slide 6
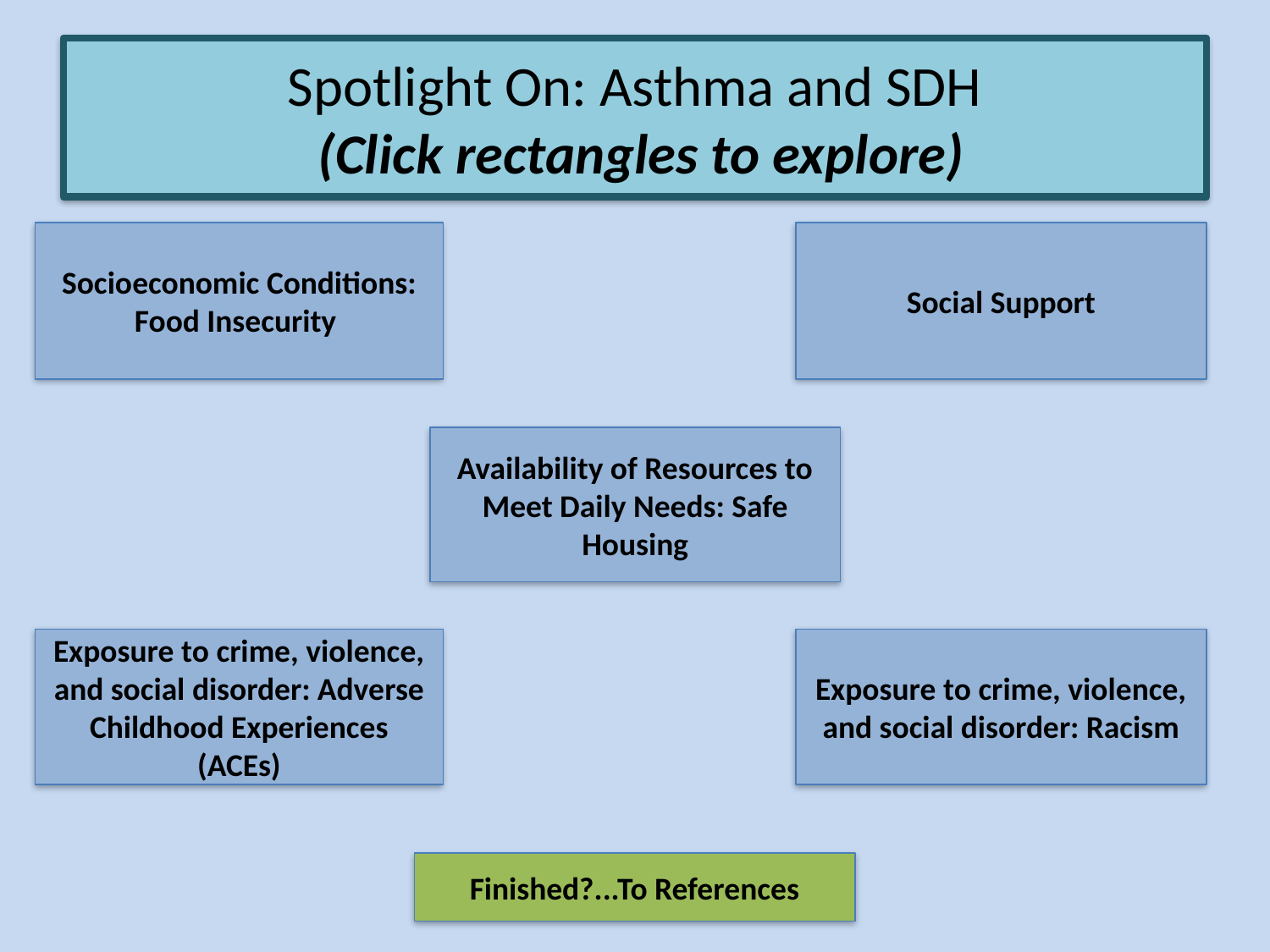

#
Spotlight On: Asthma and SDH
 (Click rectangles to explore)
Socioeconomic Conditions: Food Insecurity
Social Support
Availability of Resources to Meet Daily Needs: Safe Housing
Exposure to crime, violence, and social disorder: Adverse Childhood Experiences (ACEs)
Exposure to crime, violence, and social disorder: Racism
Finished?...To References

## Slide 7
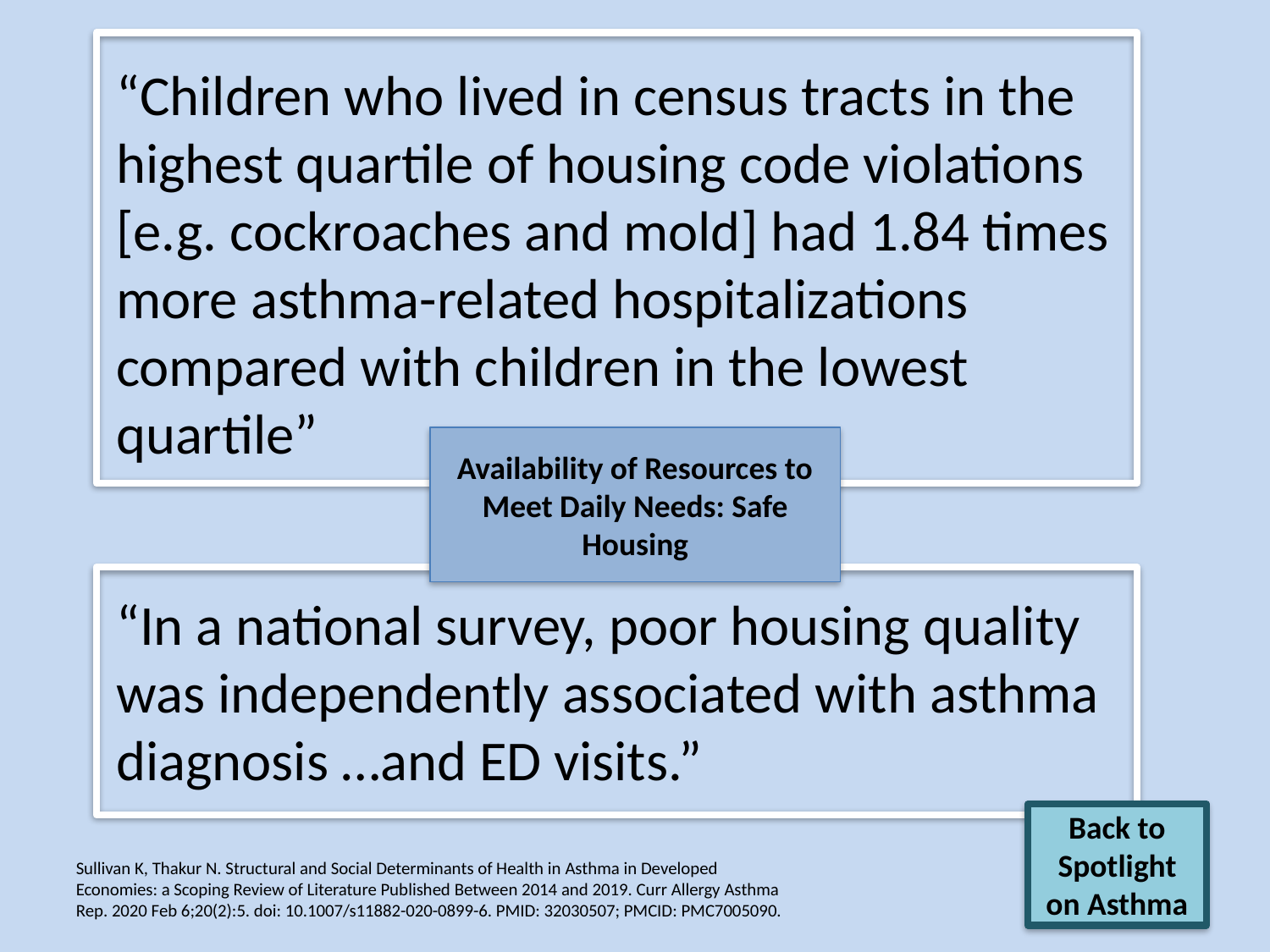

“Children who lived in census tracts in the highest quartile of housing code violations [e.g. cockroaches and mold] had 1.84 times more asthma-related hospitalizations compared with children in the lowest quartile”
Availability of Resources to Meet Daily Needs: Safe Housing
“In a national survey, poor housing quality was independently associated with asthma diagnosis …and ED visits.”
Back to Spotlight on Asthma
Sullivan K, Thakur N. Structural and Social Determinants of Health in Asthma in Developed Economies: a Scoping Review of Literature Published Between 2014 and 2019. Curr Allergy Asthma Rep. 2020 Feb 6;20(2):5. doi: 10.1007/s11882-020-0899-6. PMID: 32030507; PMCID: PMC7005090.

## Slide 8
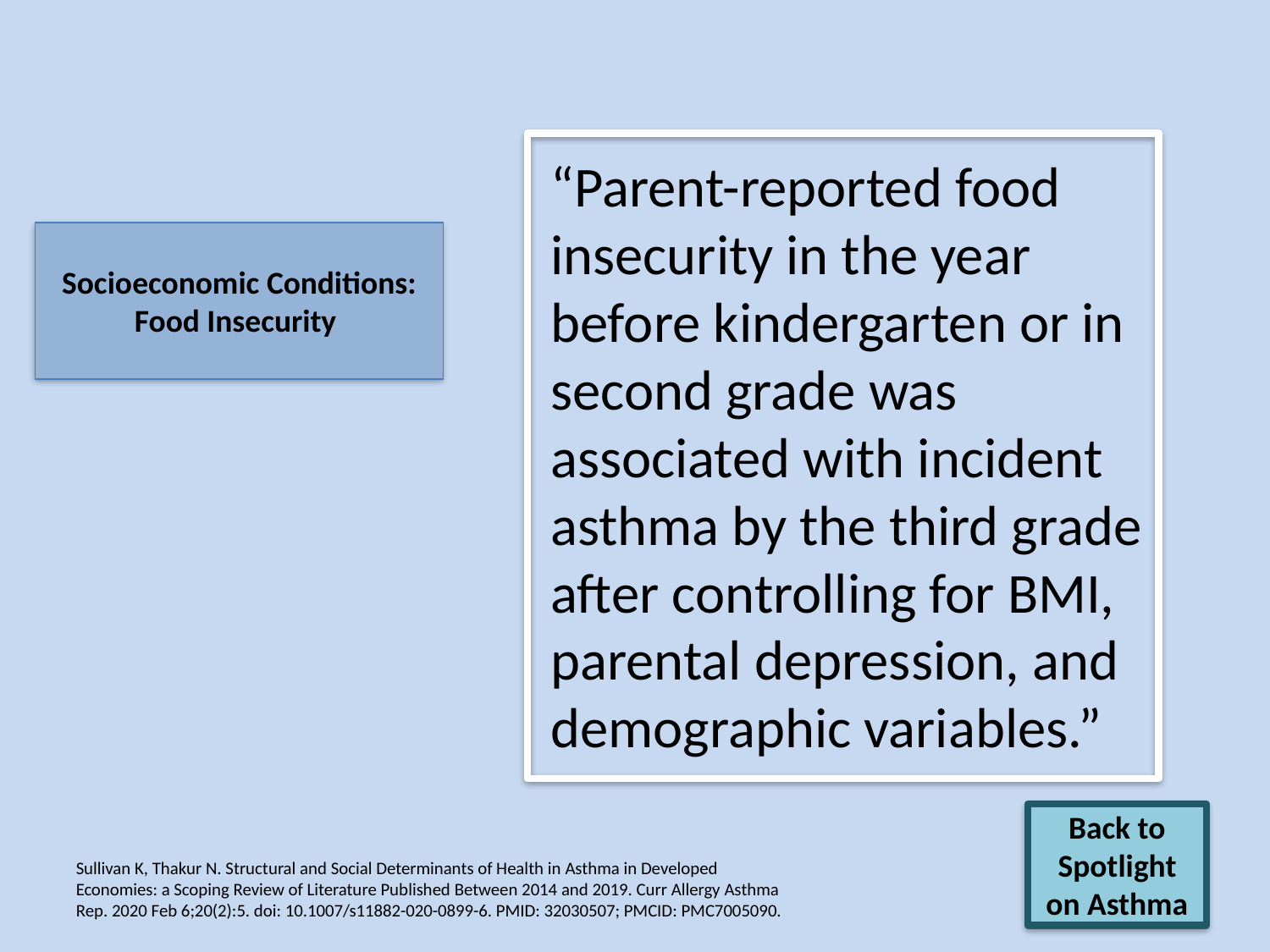

“Parent-reported food insecurity in the year before kindergarten or in second grade was associated with incident asthma by the third grade after controlling for BMI, parental depression, and demographic variables.”
Socioeconomic Conditions: Food Insecurity
Back to Spotlight on Asthma
Sullivan K, Thakur N. Structural and Social Determinants of Health in Asthma in Developed Economies: a Scoping Review of Literature Published Between 2014 and 2019. Curr Allergy Asthma Rep. 2020 Feb 6;20(2):5. doi: 10.1007/s11882-020-0899-6. PMID: 32030507; PMCID: PMC7005090.

## Slide 9
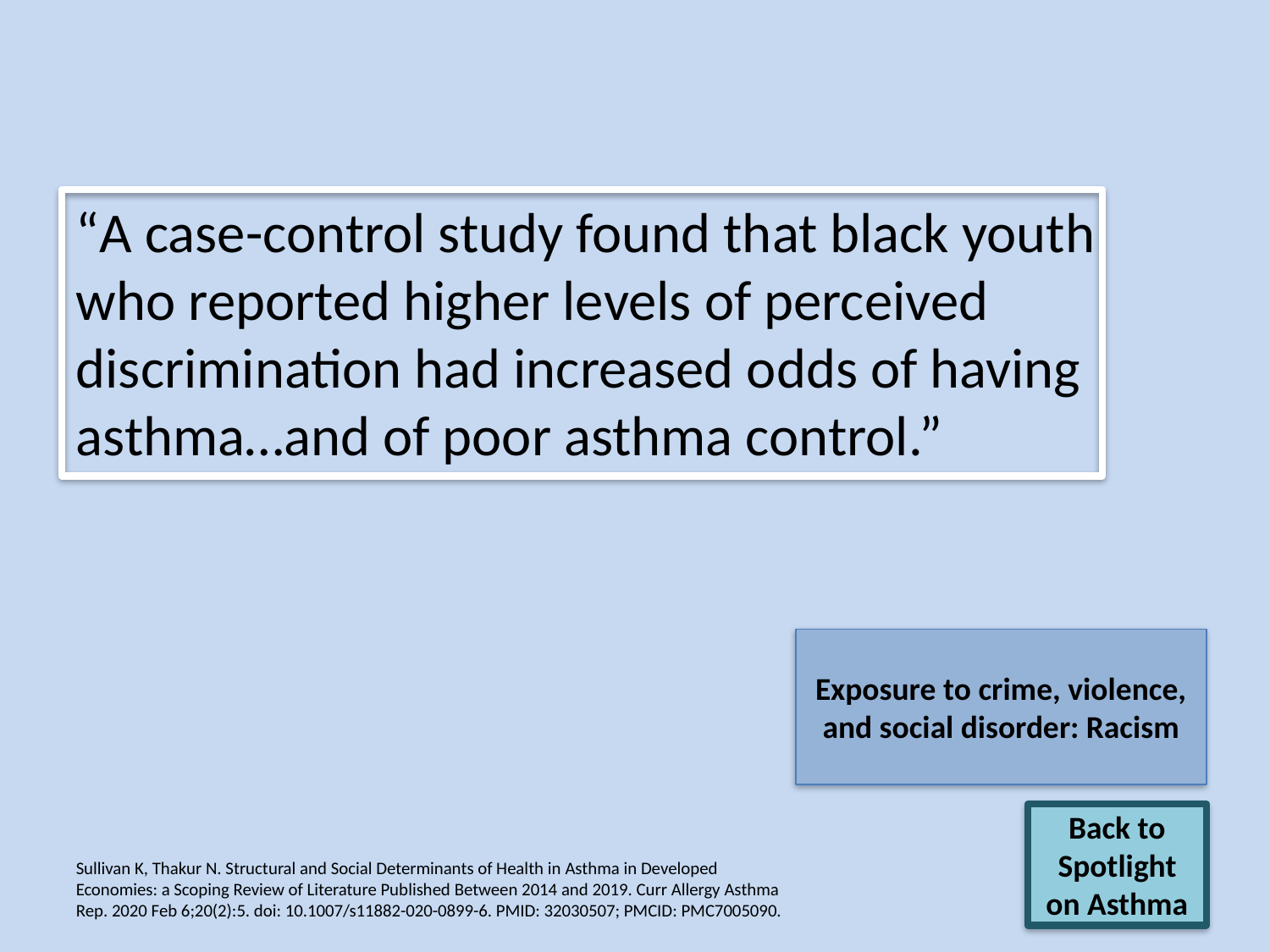

“A case-control study found that black youth who reported higher levels of perceived discrimination had increased odds of having asthma…and of poor asthma control.”
Exposure to crime, violence, and social disorder: Racism
Back to Spotlight on Asthma
Sullivan K, Thakur N. Structural and Social Determinants of Health in Asthma in Developed Economies: a Scoping Review of Literature Published Between 2014 and 2019. Curr Allergy Asthma Rep. 2020 Feb 6;20(2):5. doi: 10.1007/s11882-020-0899-6. PMID: 32030507; PMCID: PMC7005090.

## Slide 10
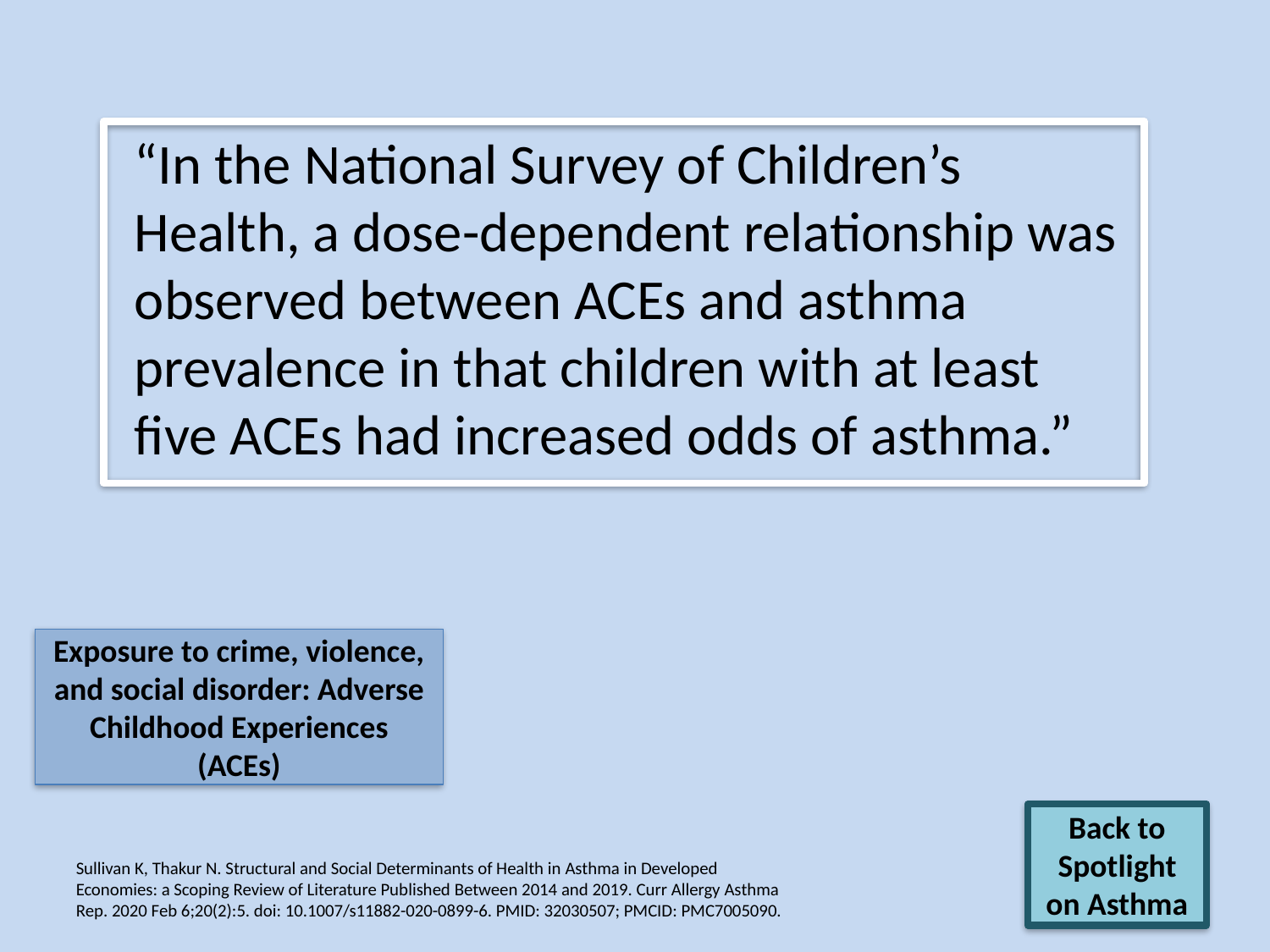

“In the National Survey of Children’s Health, a dose-dependent relationship was observed between ACEs and asthma prevalence in that children with at least five ACEs had increased odds of asthma.”
Exposure to crime, violence, and social disorder: Adverse Childhood Experiences (ACEs)
Back to Spotlight on Asthma
Sullivan K, Thakur N. Structural and Social Determinants of Health in Asthma in Developed Economies: a Scoping Review of Literature Published Between 2014 and 2019. Curr Allergy Asthma Rep. 2020 Feb 6;20(2):5. doi: 10.1007/s11882-020-0899-6. PMID: 32030507; PMCID: PMC7005090.

## Slide 11
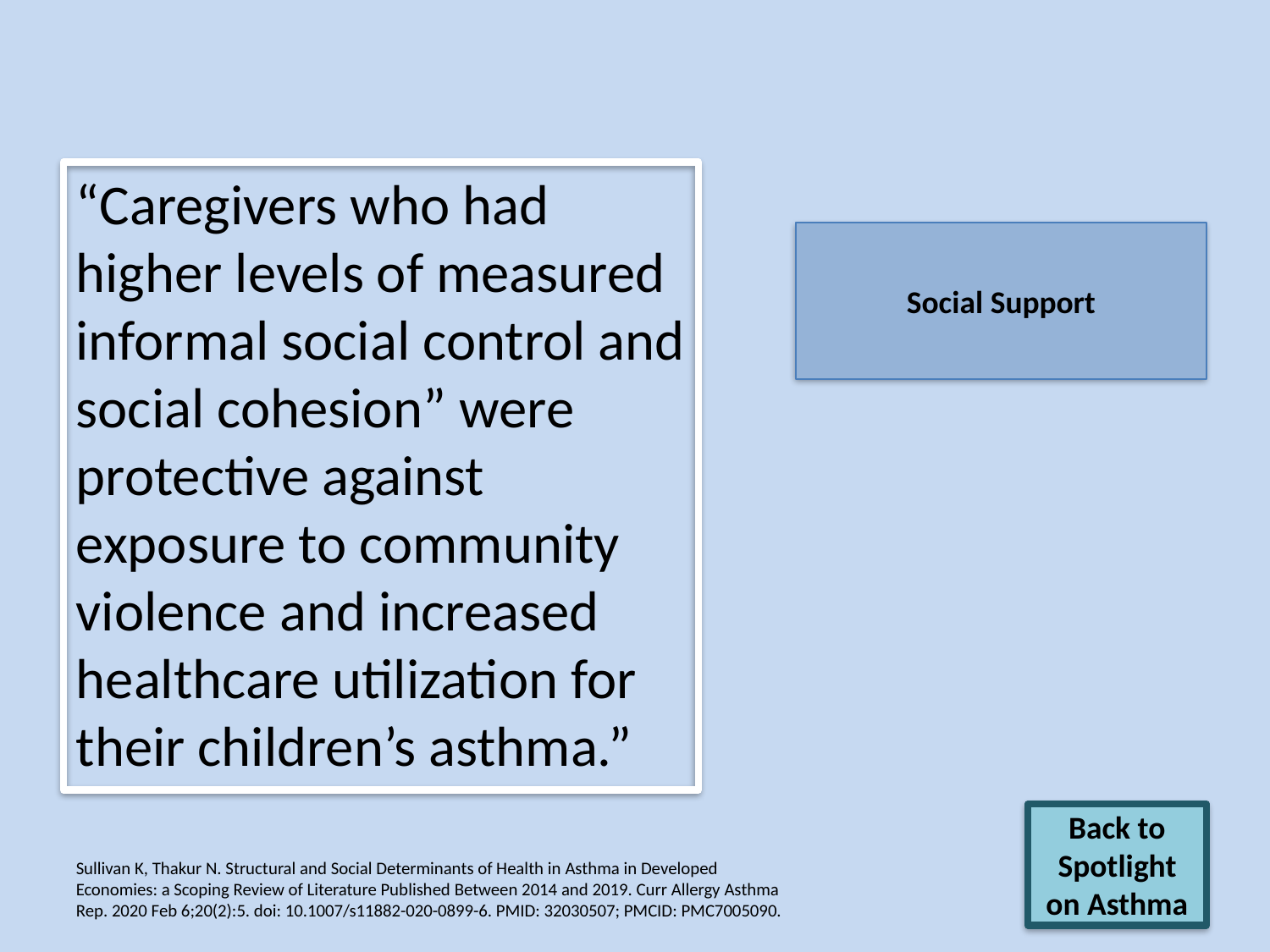

“Caregivers who had higher levels of measured informal social control and social cohesion” were protective against exposure to community violence and increased healthcare utilization for their children’s asthma.”
Social Support
Back to Spotlight on Asthma
Sullivan K, Thakur N. Structural and Social Determinants of Health in Asthma in Developed Economies: a Scoping Review of Literature Published Between 2014 and 2019. Curr Allergy Asthma Rep. 2020 Feb 6;20(2):5. doi: 10.1007/s11882-020-0899-6. PMID: 32030507; PMCID: PMC7005090.

## Slide 12
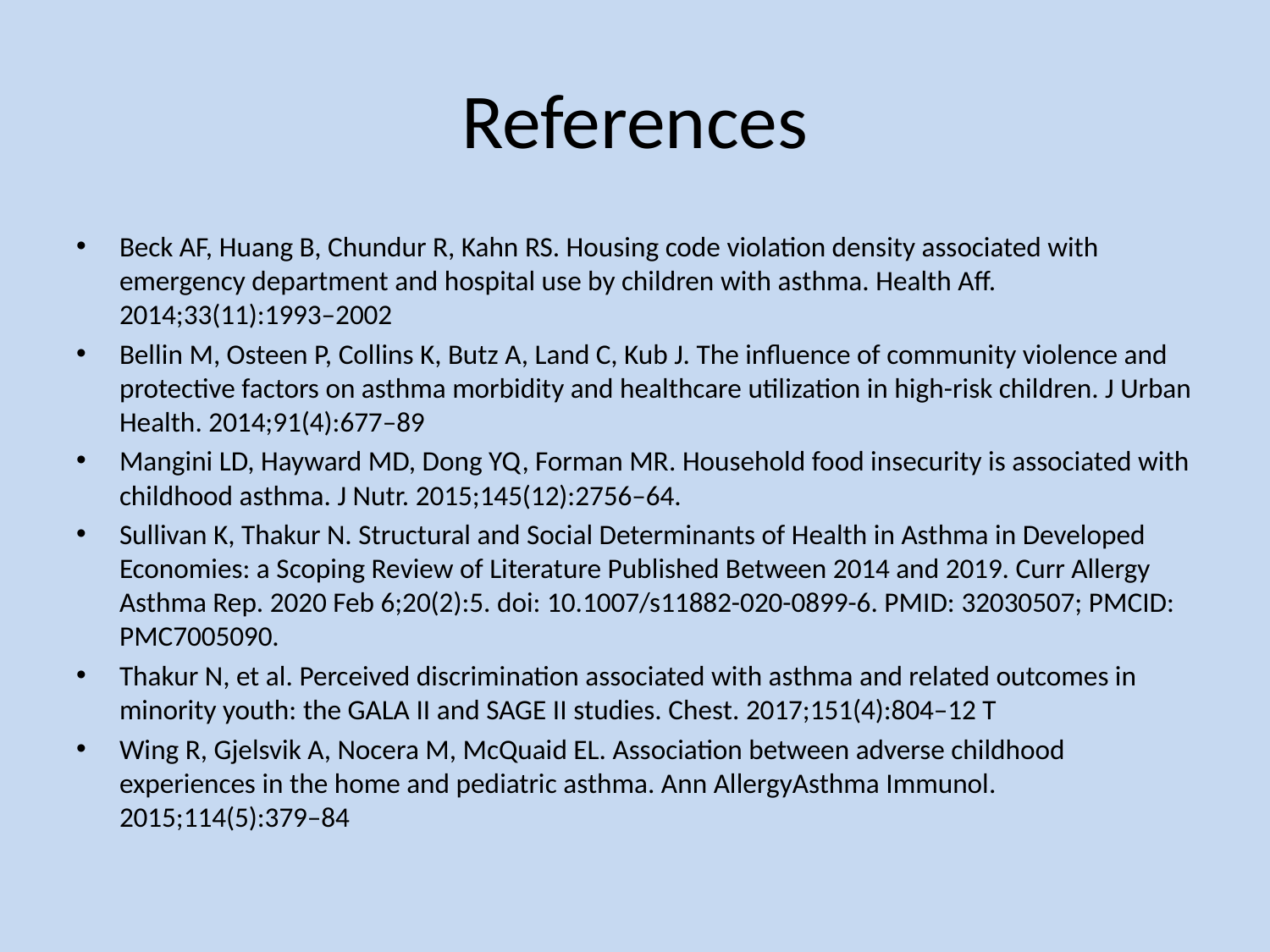

# References
Beck AF, Huang B, Chundur R, Kahn RS. Housing code violation density associated with emergency department and hospital use by children with asthma. Health Aff. 2014;33(11):1993–2002
Bellin M, Osteen P, Collins K, Butz A, Land C, Kub J. The influence of community violence and protective factors on asthma morbidity and healthcare utilization in high-risk children. J Urban Health. 2014;91(4):677–89
Mangini LD, Hayward MD, Dong YQ, Forman MR. Household food insecurity is associated with childhood asthma. J Nutr. 2015;145(12):2756–64.
Sullivan K, Thakur N. Structural and Social Determinants of Health in Asthma in Developed Economies: a Scoping Review of Literature Published Between 2014 and 2019. Curr Allergy Asthma Rep. 2020 Feb 6;20(2):5. doi: 10.1007/s11882-020-0899-6. PMID: 32030507; PMCID: PMC7005090.
Thakur N, et al. Perceived discrimination associated with asthma and related outcomes in minority youth: the GALA II and SAGE II studies. Chest. 2017;151(4):804–12 T
Wing R, Gjelsvik A, Nocera M, McQuaid EL. Association between adverse childhood experiences in the home and pediatric asthma. Ann AllergyAsthma Immunol. 2015;114(5):379–84
