# Supplementary material for: Social Determinants of Health Curriculum for the Pediatric Clerkship
Source: MedEdPORTAL. 2024 Oct 29;20:11458. doi: 10.15766/mep_2374-8265.11458 (PMC11518917; doi:10.15766/mep_2374-8265.11458)
Supplement: Supplementary file 1 — SDH Cases Faculty Supplements.docxCurriculum Orientation.pptxSDH Cases Student Handouts.docxPrework - Well Child.pptxPrework - Urgent Care.pptxPrework - Clinical Problem-solving.pptxPrework - Chronic Illness.pptxResource Assignment Orientation.pptxResource Assignment Form and Example.docxFacilitator Reminder Email.docxPresurvey and Case Analysis.docxPostsurvey and Case Analysis.docxCase Analysis Scoring Tool.docx [file mep_2374-8265.11458-s001.zip › G. Prework - Chronic Illness.pptx]

## Slide 1
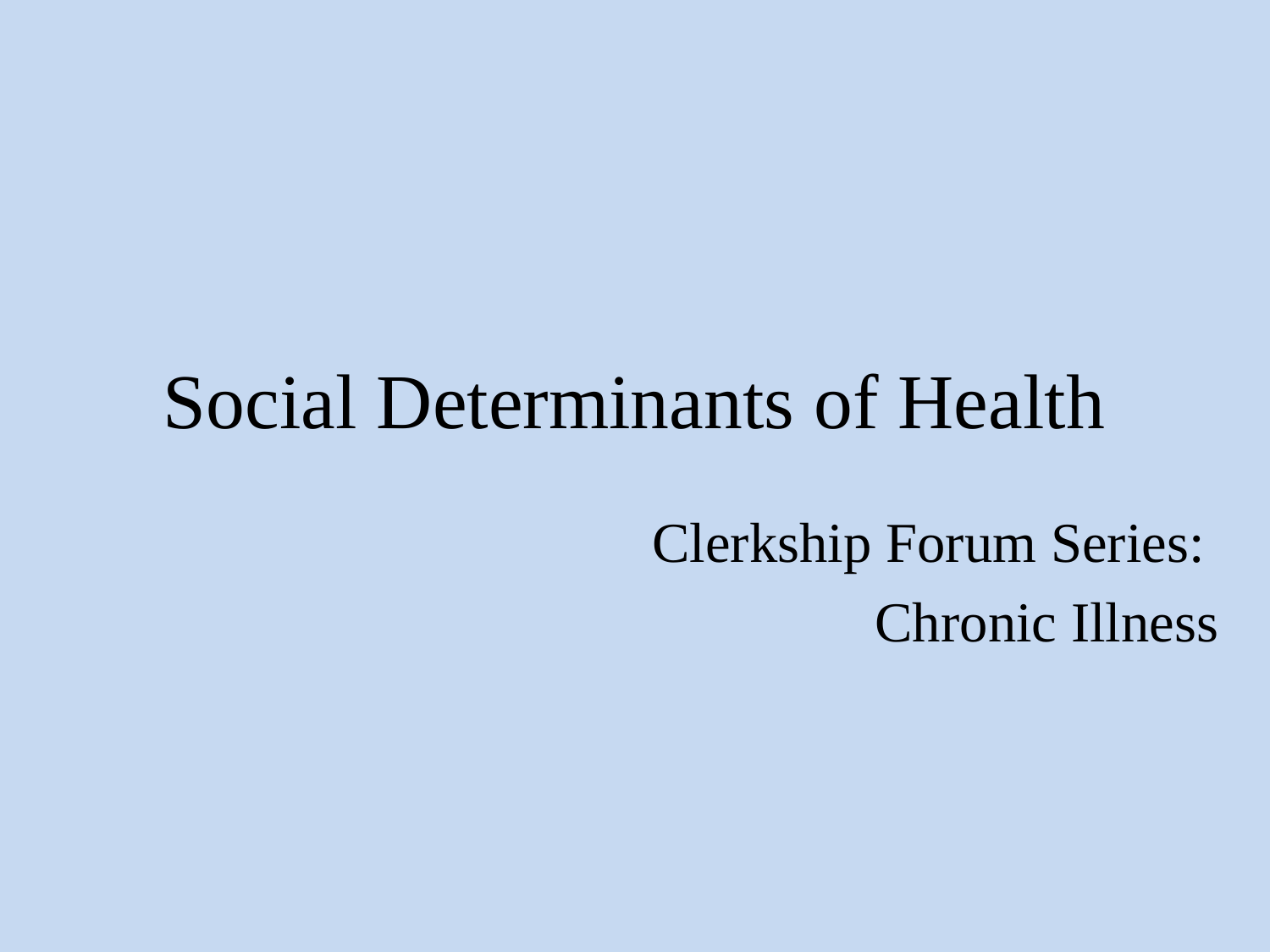

# Social Determinants of Health
Clerkship Forum Series:
Chronic Illness

## Slide 2
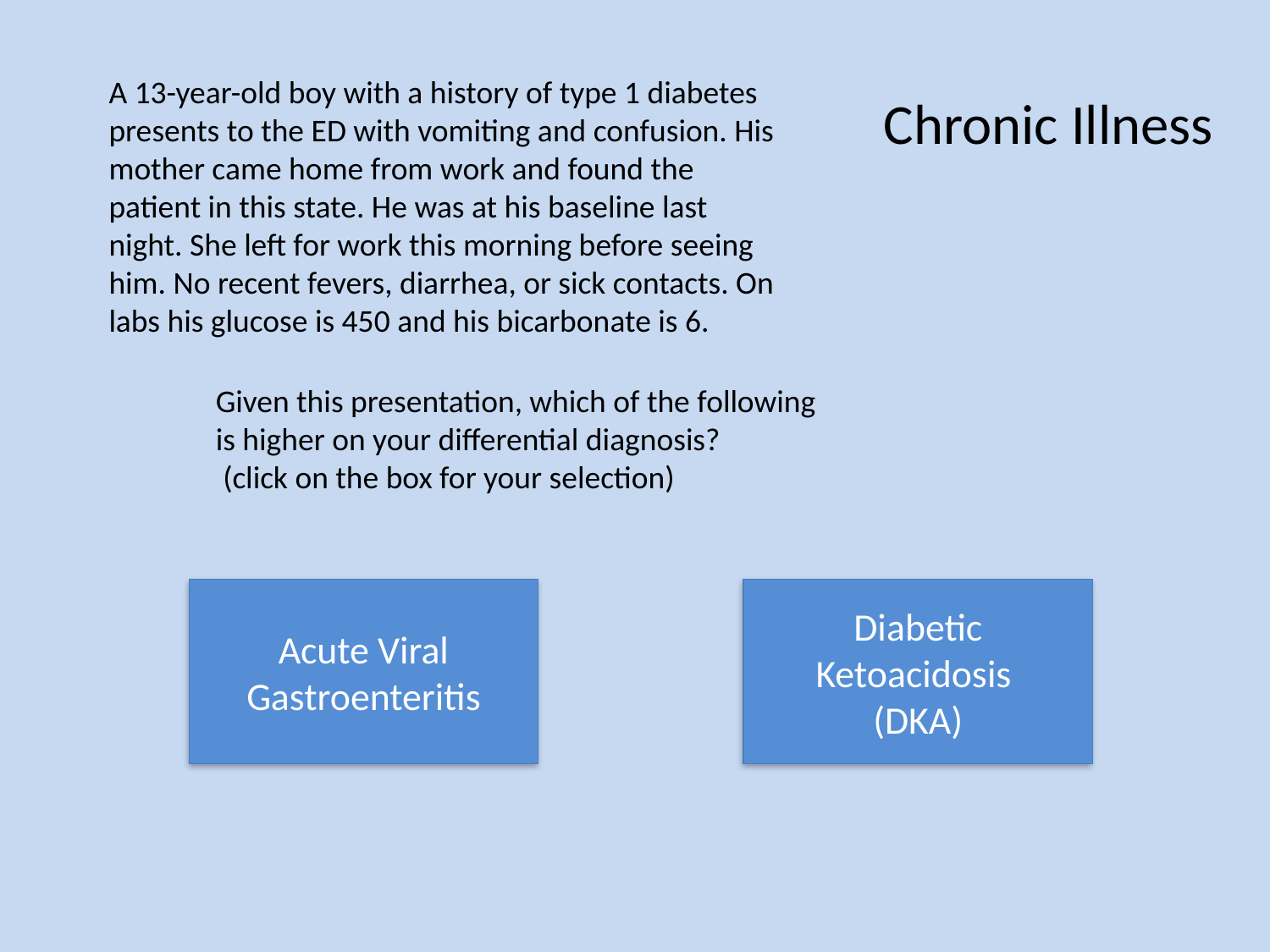

A 13-year-old boy with a history of type 1 diabetes presents to the ED with vomiting and confusion. His mother came home from work and found the patient in this state. He was at his baseline last night. She left for work this morning before seeing him. No recent fevers, diarrhea, or sick contacts. On labs his glucose is 450 and his bicarbonate is 6.
Chronic Illness
Given this presentation, which of the following is higher on your differential diagnosis?
 (click on the box for your selection)
Acute Viral Gastroenteritis
Diabetic Ketoacidosis
(DKA)

## Slide 3
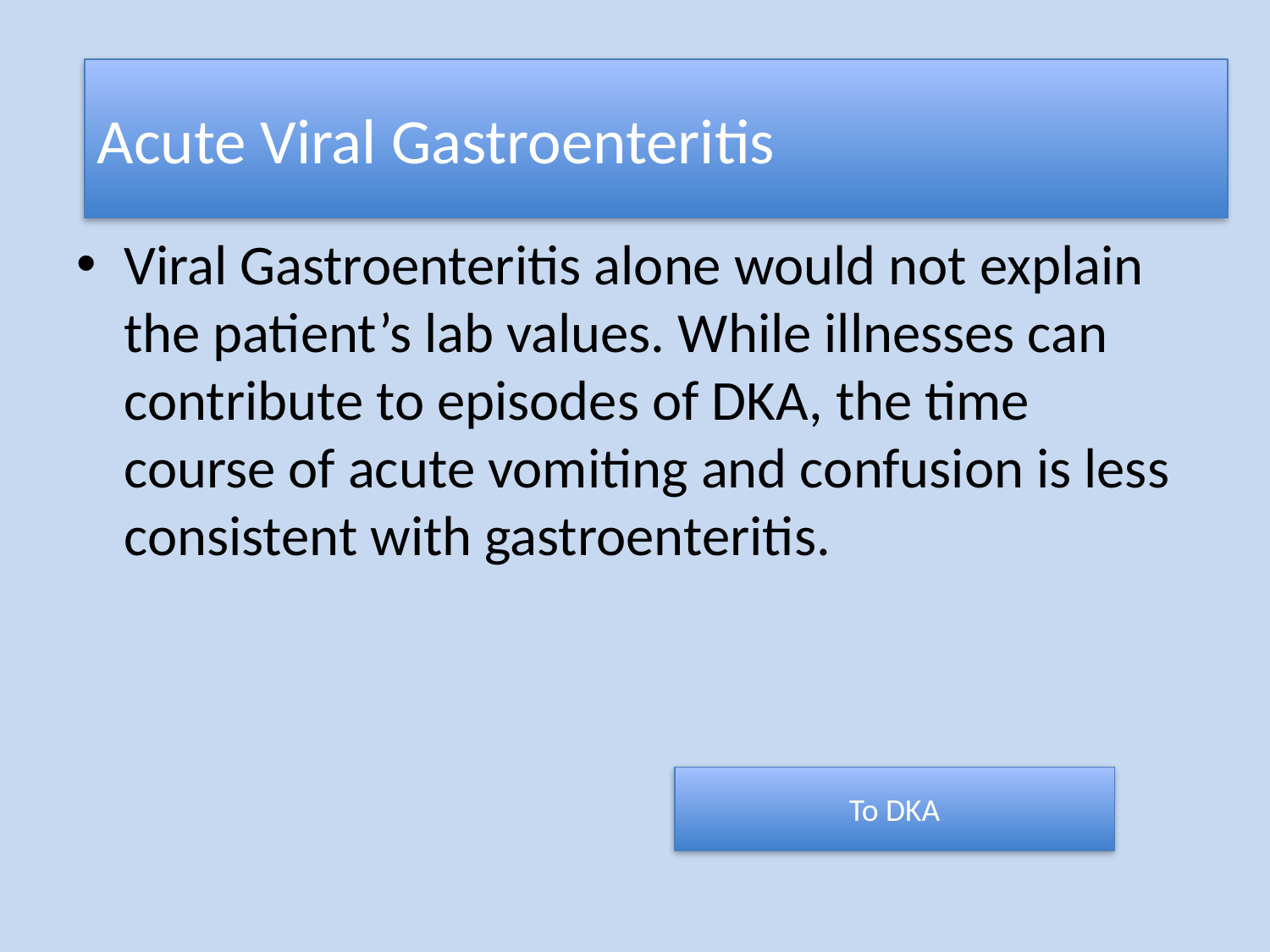

Acute Viral Gastroenteritis
Viral Gastroenteritis alone would not explain the patient’s lab values. While illnesses can contribute to episodes of DKA, the time course of acute vomiting and confusion is less consistent with gastroenteritis.
To DKA

## Slide 4
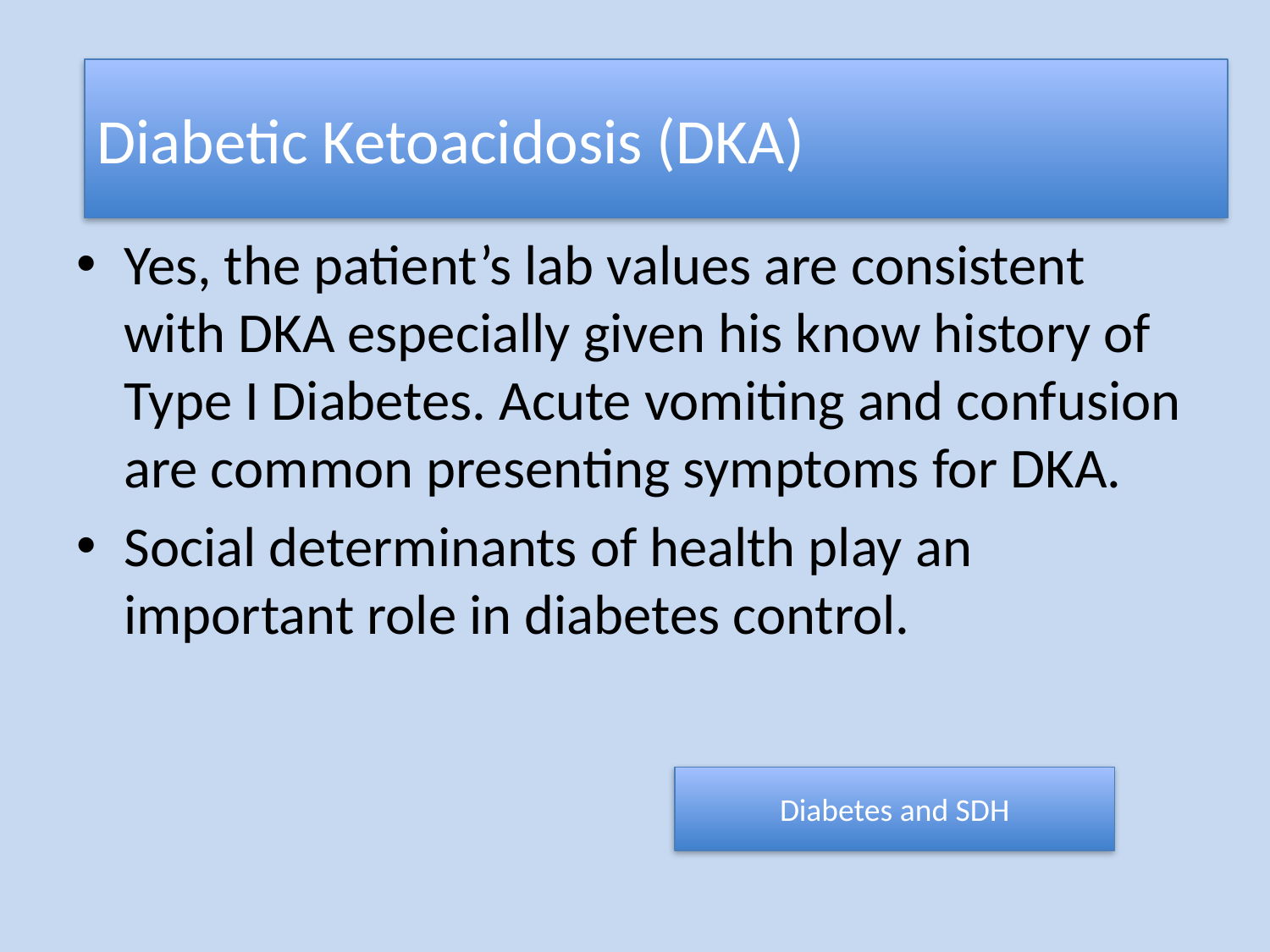

Diabetic Ketoacidosis (DKA)
Yes, the patient’s lab values are consistent with DKA especially given his know history of Type I Diabetes. Acute vomiting and confusion are common presenting symptoms for DKA.
Social determinants of health play an important role in diabetes control.
Diabetes and SDH

## Slide 5
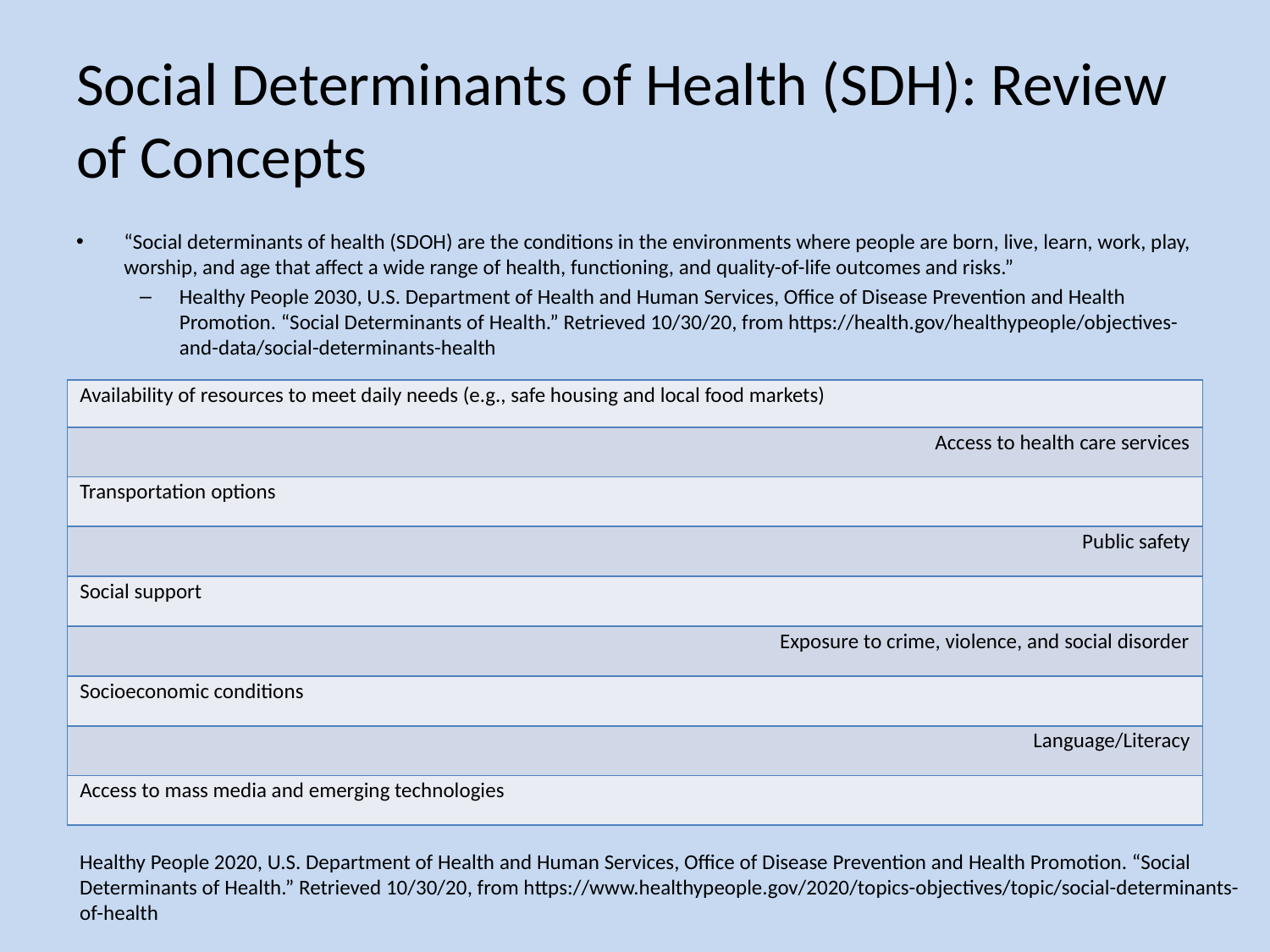

# Social Determinants of Health (SDH): Review of Concepts
“Social determinants of health (SDOH) are the conditions in the environments where people are born, live, learn, work, play, worship, and age that affect a wide range of health, functioning, and quality-of-life outcomes and risks.”
Healthy People 2030, U.S. Department of Health and Human Services, Office of Disease Prevention and Health Promotion. “Social Determinants of Health.” Retrieved 10/30/20, from https://health.gov/healthypeople/objectives-and-data/social-determinants-health
| Availability of resources to meet daily needs (e.g., safe housing and local food markets) |
| --- |
| Access to health care services |
| Transportation options |
| Public safety |
| Social support |
| Exposure to crime, violence, and social disorder |
| Socioeconomic conditions |
| Language/Literacy |
| Access to mass media and emerging technologies |
Healthy People 2020, U.S. Department of Health and Human Services, Office of Disease Prevention and Health Promotion. “Social Determinants of Health.” Retrieved 10/30/20, from https://www.healthypeople.gov/2020/topics-objectives/topic/social-determinants-of-health

## Slide 6
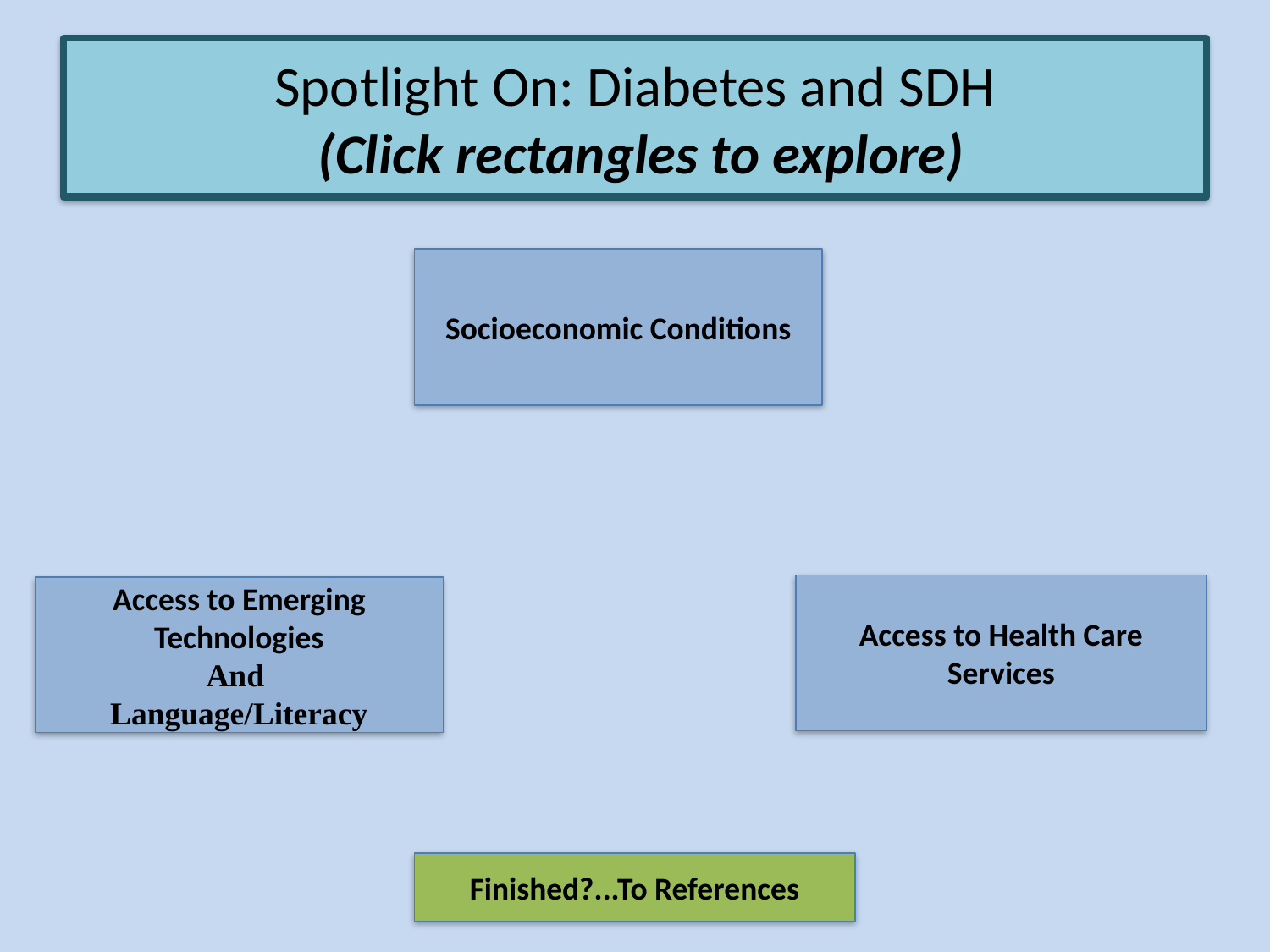

#
Spotlight On: Diabetes and SDH
 (Click rectangles to explore)
Socioeconomic Conditions
Access to Health Care Services
Access to Emerging Technologies
And
Language/Literacy
Finished?...To References

## Slide 7
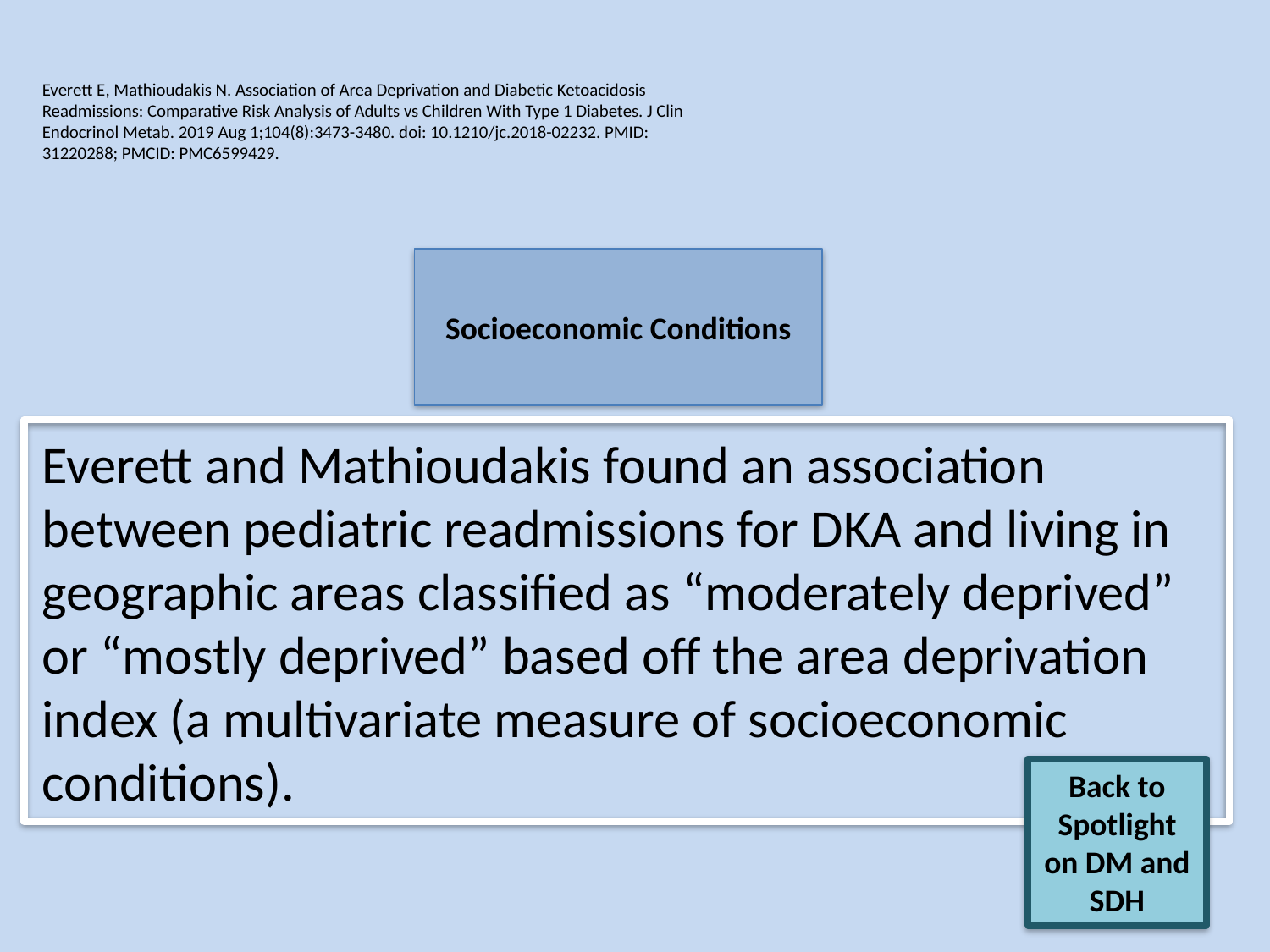

Everett E, Mathioudakis N. Association of Area Deprivation and Diabetic Ketoacidosis Readmissions: Comparative Risk Analysis of Adults vs Children With Type 1 Diabetes. J Clin Endocrinol Metab. 2019 Aug 1;104(8):3473-3480. doi: 10.1210/jc.2018-02232. PMID: 31220288; PMCID: PMC6599429.
Socioeconomic Conditions
Everett and Mathioudakis found an association between pediatric readmissions for DKA and living in geographic areas classified as “moderately deprived” or “mostly deprived” based off the area deprivation index (a multivariate measure of socioeconomic conditions).
Back to Spotlight on DM and SDH

## Slide 8
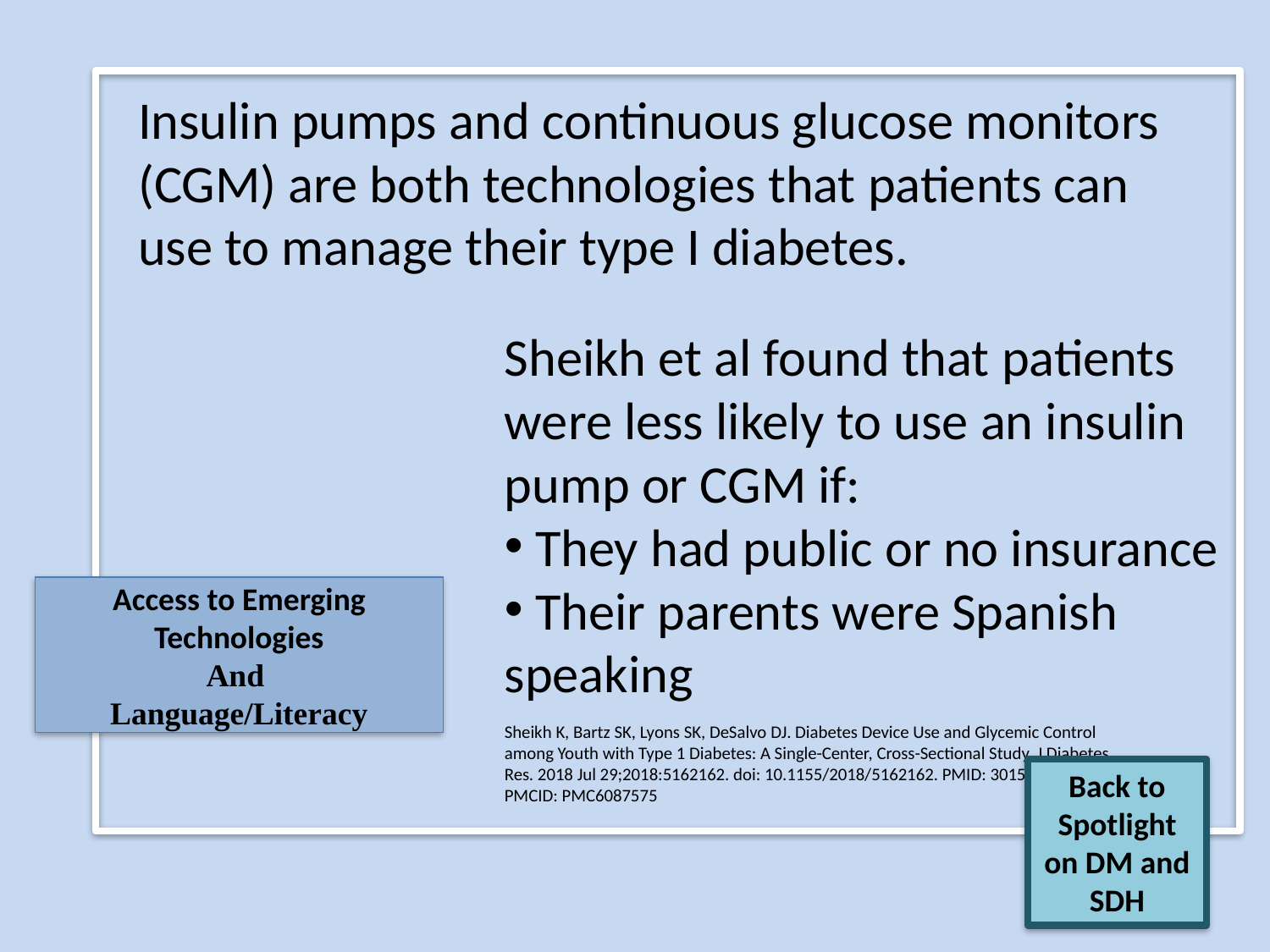

Insulin pumps and continuous glucose monitors (CGM) are both technologies that patients can use to manage their type I diabetes.
Sheikh et al found that patients were less likely to use an insulin pump or CGM if:
 They had public or no insurance
 Their parents were Spanish speaking
Access to Emerging Technologies
And
Language/Literacy
Sheikh K, Bartz SK, Lyons SK, DeSalvo DJ. Diabetes Device Use and Glycemic Control among Youth with Type 1 Diabetes: A Single-Center, Cross-Sectional Study. J Diabetes Res. 2018 Jul 29;2018:5162162. doi: 10.1155/2018/5162162. PMID: 30151393; PMCID: PMC6087575
Back to Spotlight on DM and SDH

## Slide 9
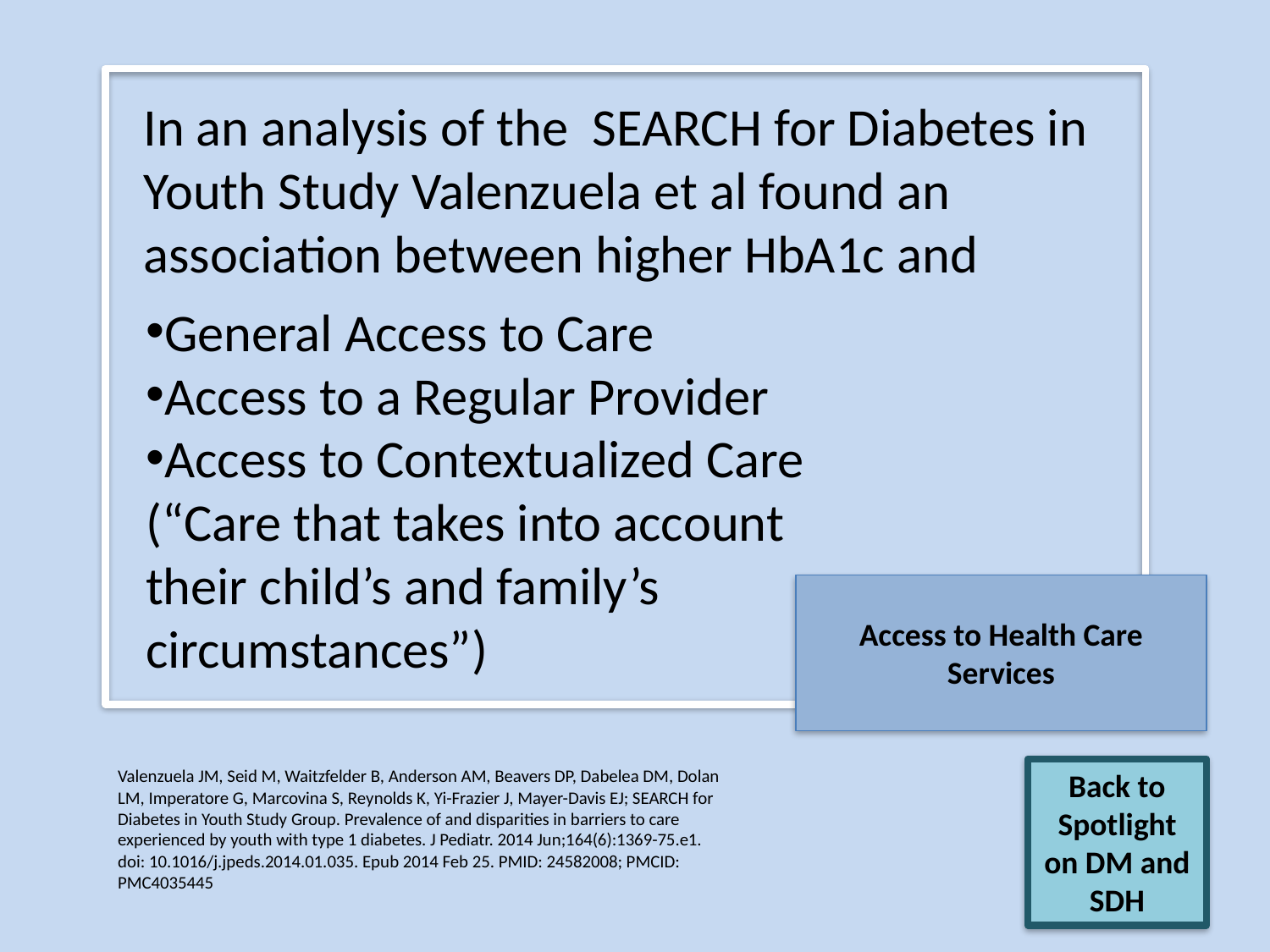

In an analysis of the SEARCH for Diabetes in Youth Study Valenzuela et al found an association between higher HbA1c and
General Access to Care
Access to a Regular Provider
Access to Contextualized Care
(“Care that takes into account their child’s and family’s circumstances”)
Access to Health Care Services
Valenzuela JM, Seid M, Waitzfelder B, Anderson AM, Beavers DP, Dabelea DM, Dolan LM, Imperatore G, Marcovina S, Reynolds K, Yi-Frazier J, Mayer-Davis EJ; SEARCH for Diabetes in Youth Study Group. Prevalence of and disparities in barriers to care experienced by youth with type 1 diabetes. J Pediatr. 2014 Jun;164(6):1369-75.e1. doi: 10.1016/j.jpeds.2014.01.035. Epub 2014 Feb 25. PMID: 24582008; PMCID: PMC4035445
Back to Spotlight on DM and SDH

## Slide 10
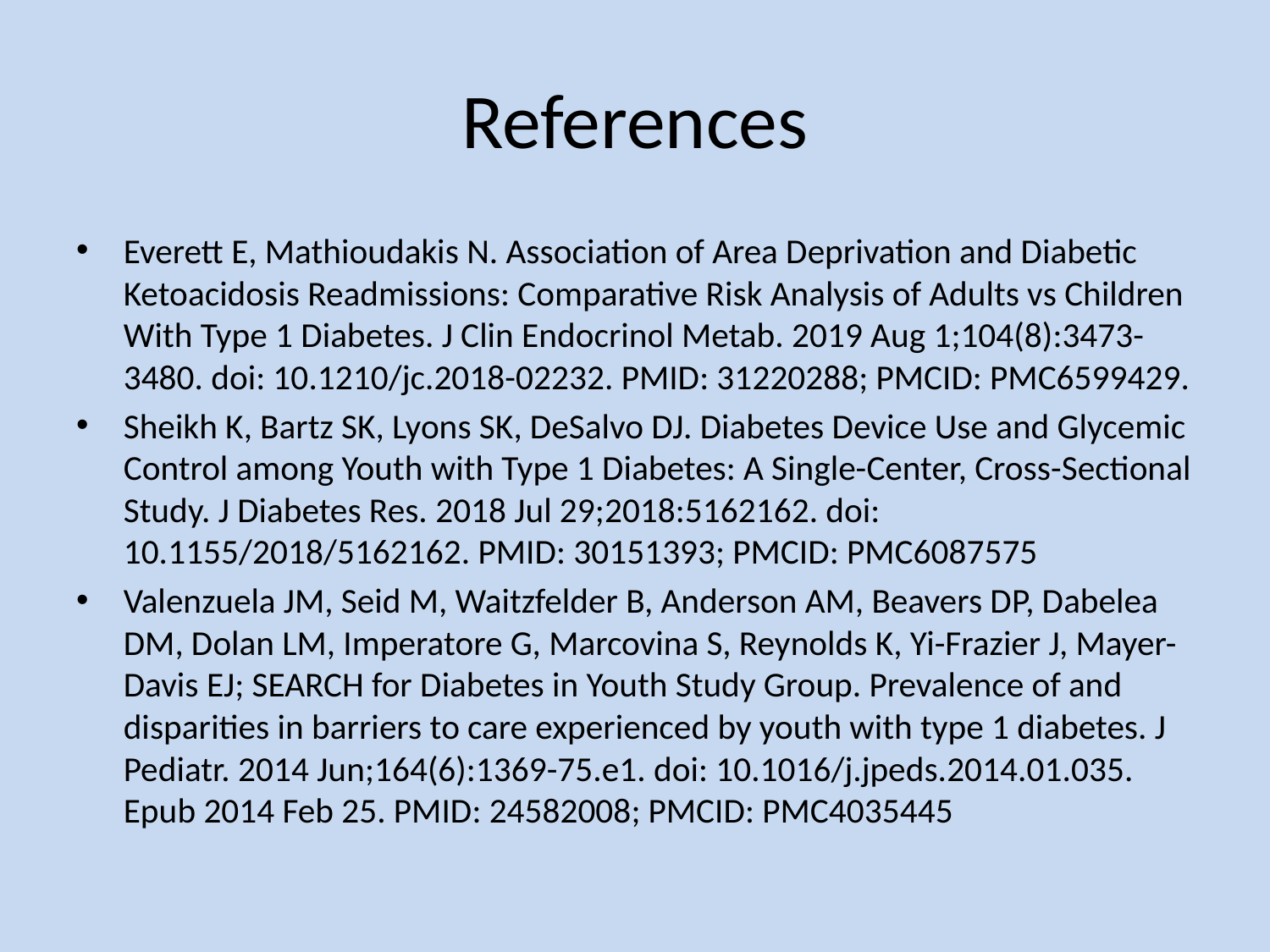

# References
Everett E, Mathioudakis N. Association of Area Deprivation and Diabetic Ketoacidosis Readmissions: Comparative Risk Analysis of Adults vs Children With Type 1 Diabetes. J Clin Endocrinol Metab. 2019 Aug 1;104(8):3473-3480. doi: 10.1210/jc.2018-02232. PMID: 31220288; PMCID: PMC6599429.
Sheikh K, Bartz SK, Lyons SK, DeSalvo DJ. Diabetes Device Use and Glycemic Control among Youth with Type 1 Diabetes: A Single-Center, Cross-Sectional Study. J Diabetes Res. 2018 Jul 29;2018:5162162. doi: 10.1155/2018/5162162. PMID: 30151393; PMCID: PMC6087575
Valenzuela JM, Seid M, Waitzfelder B, Anderson AM, Beavers DP, Dabelea DM, Dolan LM, Imperatore G, Marcovina S, Reynolds K, Yi-Frazier J, Mayer-Davis EJ; SEARCH for Diabetes in Youth Study Group. Prevalence of and disparities in barriers to care experienced by youth with type 1 diabetes. J Pediatr. 2014 Jun;164(6):1369-75.e1. doi: 10.1016/j.jpeds.2014.01.035. Epub 2014 Feb 25. PMID: 24582008; PMCID: PMC4035445
