# Supplementary material for: Social Determinants of Health Curriculum for the Pediatric Clerkship
Source: MedEdPORTAL. 2024 Oct 29;20:11458. doi: 10.15766/mep_2374-8265.11458 (PMC11518917; doi:10.15766/mep_2374-8265.11458)
Supplement: Supplementary file 1 — SDH Cases Faculty Supplements.docxCurriculum Orientation.pptxSDH Cases Student Handouts.docxPrework - Well Child.pptxPrework - Urgent Care.pptxPrework - Clinical Problem-solving.pptxPrework - Chronic Illness.pptxResource Assignment Orientation.pptxResource Assignment Form and Example.docxFacilitator Reminder Email.docxPresurvey and Case Analysis.docxPostsurvey and Case Analysis.docxCase Analysis Scoring Tool.docx [file mep_2374-8265.11458-s001.zip › H. Resource Assignment Orientation.pptx]

## Slide 1
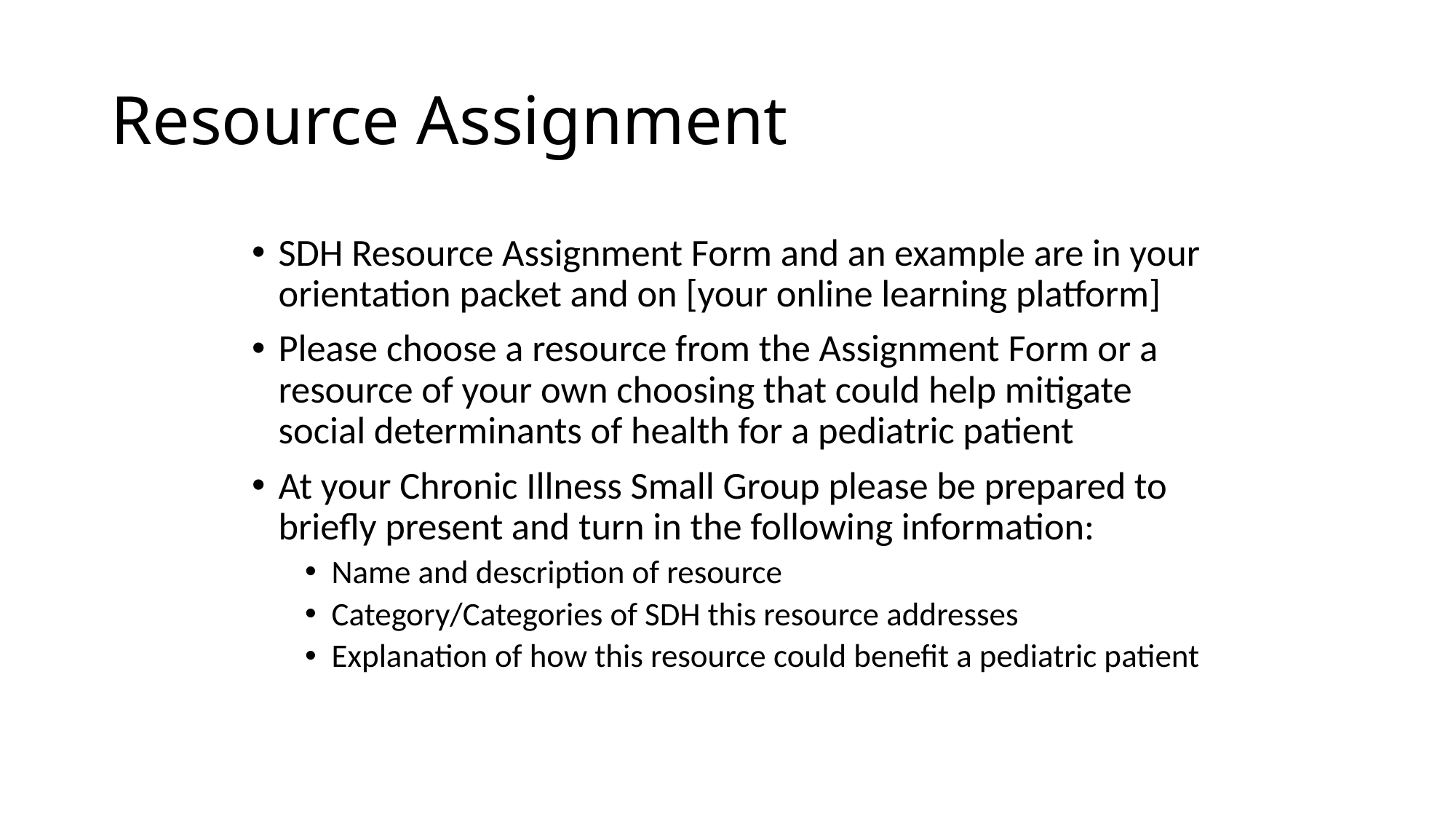

# Resource Assignment
SDH Resource Assignment Form and an example are in your orientation packet and on [your online learning platform]
Please choose a resource from the Assignment Form or a resource of your own choosing that could help mitigate social determinants of health for a pediatric patient
At your Chronic Illness Small Group please be prepared to briefly present and turn in the following information:
Name and description of resource
Category/Categories of SDH this resource addresses
Explanation of how this resource could benefit a pediatric patient
