# Supplementary material for: Social Determinants of Health Curriculum for the Pediatric Clerkship
Source: MedEdPORTAL. 2024 Oct 29;20:11458. doi: 10.15766/mep_2374-8265.11458 (PMC11518917; doi:10.15766/mep_2374-8265.11458)
Supplement: Supplementary file 1 — SDH Cases Faculty Supplements.docxCurriculum Orientation.pptxSDH Cases Student Handouts.docxPrework - Well Child.pptxPrework - Urgent Care.pptxPrework - Clinical Problem-solving.pptxPrework - Chronic Illness.pptxResource Assignment Orientation.pptxResource Assignment Form and Example.docxFacilitator Reminder Email.docxPresurvey and Case Analysis.docxPostsurvey and Case Analysis.docxCase Analysis Scoring Tool.docx [file mep_2374-8265.11458-s001.zip › I. Resource Assignment Form and Example.docx]

Instructions for Use: Both the Social Determinants of Health (SDH) Resource Assignment Form and Social Determinants of Health (SDH) Resource Assignment Example should be given to students at the start of the curriculum. Students should complete the resource assignment by the Chronic Illness Small Group during which they will give a 1-minute presentation. Student orientation to the assignment can be found in Appendix H.

**Social Determinants of Health (SDH) Resource Assignment Form**

**DUE** at Chronic Illness Small Group

Give ~ 1 minute oral presentation and turn in completed form below.

Select a resource from the list below OR research a resource not on the list of interest to you.

[List of local resources, i.e. for New Orleans:]

Medicaid Cab

Boys and Girls Club

GoodRx

CHIP

Boys Town Louisiana

Edible School Yard New Orleans

Children’s Bureau of New Orleans

Crescent City Community Land Trust

Good to Go NOLA

Top Box Foods New Orleans

Grow Dat
Providence Community Housing

Center for Resilience

New Orleans Family Justice Center

Broadmoor Improvement Association

Choose Safe Places for Early Care and Education

Children’s Special Health Services (CSHS)

Text4baby

Name of resource:

_________________________________________________________________

Mark (X) which category/categories of SDH your resource addresses.

____Availability of resources to meet daily needs

____Access to health care services

____Transportation options

____Public safety

____Social support

____Exposure to crime, violence, and social disorder

____Socioeconomic conditions

____Language/Literacy

____Access to mass media and emerging technologies

1-3 sentence description of resource:

1-3 sentence explanation of how this resource could benefit a pediatric patient.

***Example*: Social Determinants of Health (SDH) Resource Assignment**

Name of resource:

Special Supplementation Nutrition Program for Women, Infants, and Children (aka WIC)

Mark (X) which category/categories of SDH your resource addresses.

_X__ Availability of resources to meet daily needs

_X__ Access to health care services

____ Transportation options

____ Public safety

____ Social support

____ Exposure to crime, violence, and social disorder

_X__ Socioeconomic conditions

____ Language/Literacy

____ Access to mass media and emerging technologies

1-3 sentence description of resource:

Federal grant program which funds nutritional support for low-income pregnant and breastfeeding or post-partum women, infants, and children <5 y/o. There are currently six WIC clinics in New Orleans. At these clinics, mothers and their children can be enrolled and receive the benefits of nutritional education including healthy eating and breastfeeding support, funds to buy healthy foods and infant formula, and referrals to other programs which provide additional support.

1-3 sentence explanation of how this resource could benefit a pediatric patient.

WIC would provide funds to help feed a pediatric patient which could alleviate socioeconomic conditions that might otherwise result in a child being malnourished or undernourished. There are several common approved grocery stores including Wal-Mart, Rouses, Winn Dixie, Save a Lot and Rouses to help with availability of resources (food) to meet daily needs. WIC also facilitates access to other services including healthcare services such as vaccines.
