# Supplementary material for: Social Determinants of Health Curriculum for the Pediatric Clerkship
Source: MedEdPORTAL. 2024 Oct 29;20:11458. doi: 10.15766/mep_2374-8265.11458 (PMC11518917; doi:10.15766/mep_2374-8265.11458)
Supplement: Supplementary file 1 — SDH Cases Faculty Supplements.docxCurriculum Orientation.pptxSDH Cases Student Handouts.docxPrework - Well Child.pptxPrework - Urgent Care.pptxPrework - Clinical Problem-solving.pptxPrework - Chronic Illness.pptxResource Assignment Orientation.pptxResource Assignment Form and Example.docxFacilitator Reminder Email.docxPresurvey and Case Analysis.docxPostsurvey and Case Analysis.docxCase Analysis Scoring Tool.docx [file mep_2374-8265.11458-s001.zip › J. Faciliator Reminder Email.docx]

**Instructions for Use:** The following reminder email should be sent to the faculty members facilitating the SDH curriculum early in the week that their students are scheduled to meet for the Chronic Illness Small Group.

Dear Small Group Facilitator,

It looks like your group is scheduled for their Chronic Illness Small Group this week. I wanted to reach out as there are additional tasks related to this final small group.

For your Chronic Illness Small Group this week, there are three tasks related to the Social Determinants of Health Curriculum:

1. The weekly Social Determinants of Health Case
2. The resource presentation: The students should have very brief (~1 minute) presentations about a resource they identified to help address a social determinant of health for a pediatric patient. They should also turn in a document containing this information to you.
3. Post-Survey: The students were sent an email titled "Pediatric Clerkship Post-Survey: Open during or after Chronic Illness Small Group" today (Dec 6) and instructed to wait until during or after the small group to complete the survey. If you could allow a few minutes at the end of the small group for them to complete this survey that would be very helpful. I estimate the survey should only take them ~5 minutes to complete.

Thank you for all that you are doing for our medical students' education. Please let me know if you have any questions or if you have any feedback on the SDH material.

Thanks!
