# Supplementary material for: Social Determinants of Health Curriculum for the Pediatric Clerkship
Source: MedEdPORTAL. 2024 Oct 29;20:11458. doi: 10.15766/mep_2374-8265.11458 (PMC11518917; doi:10.15766/mep_2374-8265.11458)
Supplement: Supplementary file 1 — SDH Cases Faculty Supplements.docxCurriculum Orientation.pptxSDH Cases Student Handouts.docxPrework - Well Child.pptxPrework - Urgent Care.pptxPrework - Clinical Problem-solving.pptxPrework - Chronic Illness.pptxResource Assignment Orientation.pptxResource Assignment Form and Example.docxFacilitator Reminder Email.docxPresurvey and Case Analysis.docxPostsurvey and Case Analysis.docxCase Analysis Scoring Tool.docx [file mep_2374-8265.11458-s001.zip › L. Postsurvey and Case Analysis.docx]

**Instructions for Use:** The following post-survey and case analysis should be completed by the students after the conclusion of the SDH curriculum. Students should be able to complete the survey and case analysis in 5-10 minutes. The case can be scored using the tool provided in Appendix M.

Social Determinants of Health Post-Survey

Please review the following case and provide your answers to the questions that follow.

Alexa is a 6-year-old girl with a history of wheezing presenting to the ED with cough, difficulty breathing, and wheezing. Symptoms began 2 days ago with cough but today her mother noted that she was using extra muscles to breath and wheezing. She has also had a runny nose and decreased appetite. No fever, change in urine output, rash, N/V, or diarrhea. No known sick contacts. She has been seen twice in the ED during the past year with similar symptoms which responded to albuterol and ipratropium nebulized treatments and oral steroids. During her last visit the family was given an albuterol inhaler at ED discharge, but her mother reports it has run out. She has not seen her pediatrician since the last ED visit. She is on no regular medications. She lives with her mother and four siblings but spends weekends at her father’s house. Her father smokes outside his house. She is up to date on all her immunizations except the influenza vaccine which she has not received in 2 years.

What is your leading diagnosis?

Please name at least 4 factors which you think could be contributing to her health status:

What are at least four additional questions you would like to ask the patient and her family?

Please rate the following statements:

I can accurately define the concept of social determinants of health.

___ Strongly disagree

___ Disagree

___ Somewhat Disagree

___ Neither agree nor disagree

___ Somewhat Agree

___ Agree

___ Strongly Agree

I am confident in identifying social determinants of health that commonly affect pediatric patients.

___ Strongly disagree

___ Disagree

___ Somewhat Disagree

___ Neither agree nor disagree

___ Somewhat Agree

___ Agree

___ Strongly Agree

I am confident in analyzing a pediatric case for social determinants of health which may be affecting the patient.

___ Strongly disagree

___ Disagree

___ Somewhat Disagree

___ Neither agree nor disagree

___ Somewhat Agree

___ Agree

___ Strongly Agree

I feel comfortable forming questions to elicit social determinants of health for a pediatric patient.

___ Strongly disagree

___ Disagree

___ Somewhat Disagree

___ Neither agree nor disagree

___ Somewhat Agree

___ Agree

___ Strongly Agree

I can identify resources to help mitigate effects of social determinants of health for pediatric patients.

___ Strongly disagree

___ Disagree

___ Somewhat Disagree

___ Neither agree nor disagree

___ Somewhat Agree

___ Agree

___ Strongly Agree

Learning about social determinants of health is valuable to my medical education.

___ Strongly disagree

___ Disagree

___ Somewhat Disagree

___ Neither agree nor disagree

___ Somewhat Agree

___ Agree

___ Strongly Agree

Pediatric small groups advanced my understanding of social determinants of health.

___ Strongly disagree

___ Disagree

___ Somewhat Disagree

___ Neither agree nor disagree

___ Somewhat Agree

___ Agree

___ Strongly Agree

How often did you discuss social determinants of health in your small group sessions?

Never Once 2-3 times 4-5 times >6 times

How often did you discuss social determinants of health in the clinical setting?

Never Once 2-3 times 4-5 times >6 times

Do you have any additional comments about the social determinants of health curriculum?
