# Supplementary material for: Social Determinants of Health Curriculum for the Pediatric Clerkship
Source: MedEdPORTAL. 2024 Oct 29;20:11458. doi: 10.15766/mep_2374-8265.11458 (PMC11518917; doi:10.15766/mep_2374-8265.11458)
Supplement: Supplementary file 1 — SDH Cases Faculty Supplements.docxCurriculum Orientation.pptxSDH Cases Student Handouts.docxPrework - Well Child.pptxPrework - Urgent Care.pptxPrework - Clinical Problem-solving.pptxPrework - Chronic Illness.pptxResource Assignment Orientation.pptxResource Assignment Form and Example.docxFacilitator Reminder Email.docxPresurvey and Case Analysis.docxPostsurvey and Case Analysis.docxCase Analysis Scoring Tool.docx [file mep_2374-8265.11458-s001.zip › M. Case Analysis Scoring Tool.docx]

**Instructions for use**: The following document should be used to score student responses for the pre and post-case analysis in relation to SDH. There are many potentially correct answers unrelated to SDH which this tool was not designed to assess. This tool is not designed to achieve a specific grade, rather to evaluate change after receipt of the curriculum.

**Scoring Tool for Case Analysis**

Alexa is a 6-year-old girl with a history of wheezing presenting to the ED with cough, difficulty breathing, and wheezing. Symptoms began 2 days ago with cough but today her mother noted that she was using extra muscles to breath and wheezing. She has also had a runny nose and decreased appetite. No fever, change in urine output, rash, N/V, or diarrhea. No known sick contacts. She has been seen twice in the ED during the past year with similar symptoms which responded to albuterol and ipratropium nebulized treatments and oral steroids. During her last visit the family was given an albuterol inhaler at ED discharge, but her mother reports it has run out. She has not seen her pediatrician since the last ED visit. She is on no regular medications. She lives with her mother and four siblings but spends weekends at her father’s house. Her father smokes outside his house. She is up to date on all her immunizations except the influenza vaccine which she has not received in 2 years.

What is your leading diagnosis?

*Acceptable answers include asthma, asthma exacerbation, mild persistent asthma*

Please name at least 4 factors which you think could be contributing to her health status:

*Students receive a point for each factor related to the following categories based on the Social Determinants of Health listed for Healthy People 2030^1^:*

- *Economic Stability (e.g. employment, food insecurity, housing insecurity, poverty)*
- *Education Access and Quality (e.g. early childhood development and education, enrollment in higher education, high school graduation, language and literacy)*
- *Health care access and quality (e.g. access to health services, access to primary care, health literacy)*
- *Neighborhood and Built Environment (e.g. access to foods that support healthy eating patterns, crime and violence, environmental conditions, quality of housing)*
- *Social and Community Context (e.g. civic participation, discrimination, incarceration, social cohesion)*

What are at least four additional questions you would like to ask the patient and her family?

*Students receive a point for each factor related to the following categories based on the Social Determinants of Health listed for Healthy People 2030^1^:*

- *Economic Stability (e.g. employment, food insecurity, housing insecurity, poverty)*
- *Education Access and Quality (e.g. early childhood development and education, enrollment in higher education, high school graduation, language and literacy)*
- *Health care access and quality (e.g. access to health services, access to primary care, health literacy)*
- *Neighborhood and Built Environment (e.g. access to foods that support healthy eating patterns, crime and violence, environmental conditions, quality of housing)*
- *Social and Community Context (e.g. civic participation, discrimination, incarceration, social cohesion)*
