# Supplementary material for: Social Determinants of Health Curriculum for the Pediatric Clerkship
Source: MedEdPORTAL. 2024 Oct 29;20:11458. doi: 10.15766/mep_2374-8265.11458 (PMC11518917; doi:10.15766/mep_2374-8265.11458)
Supplement: Supplementary file 1 — SDH Cases Faculty Supplements.docxCurriculum Orientation.pptxSDH Cases Student Handouts.docxPrework - Well Child.pptxPrework - Urgent Care.pptxPrework - Clinical Problem-solving.pptxPrework - Chronic Illness.pptxResource Assignment Orientation.pptxResource Assignment Form and Example.docxFacilitator Reminder Email.docxPresurvey and Case Analysis.docxPostsurvey and Case Analysis.docxCase Analysis Scoring Tool.docx [file mep_2374-8265.11458-s001.zip › F. Prework - Clinical Problem-solving.pptx]

## Slide 1
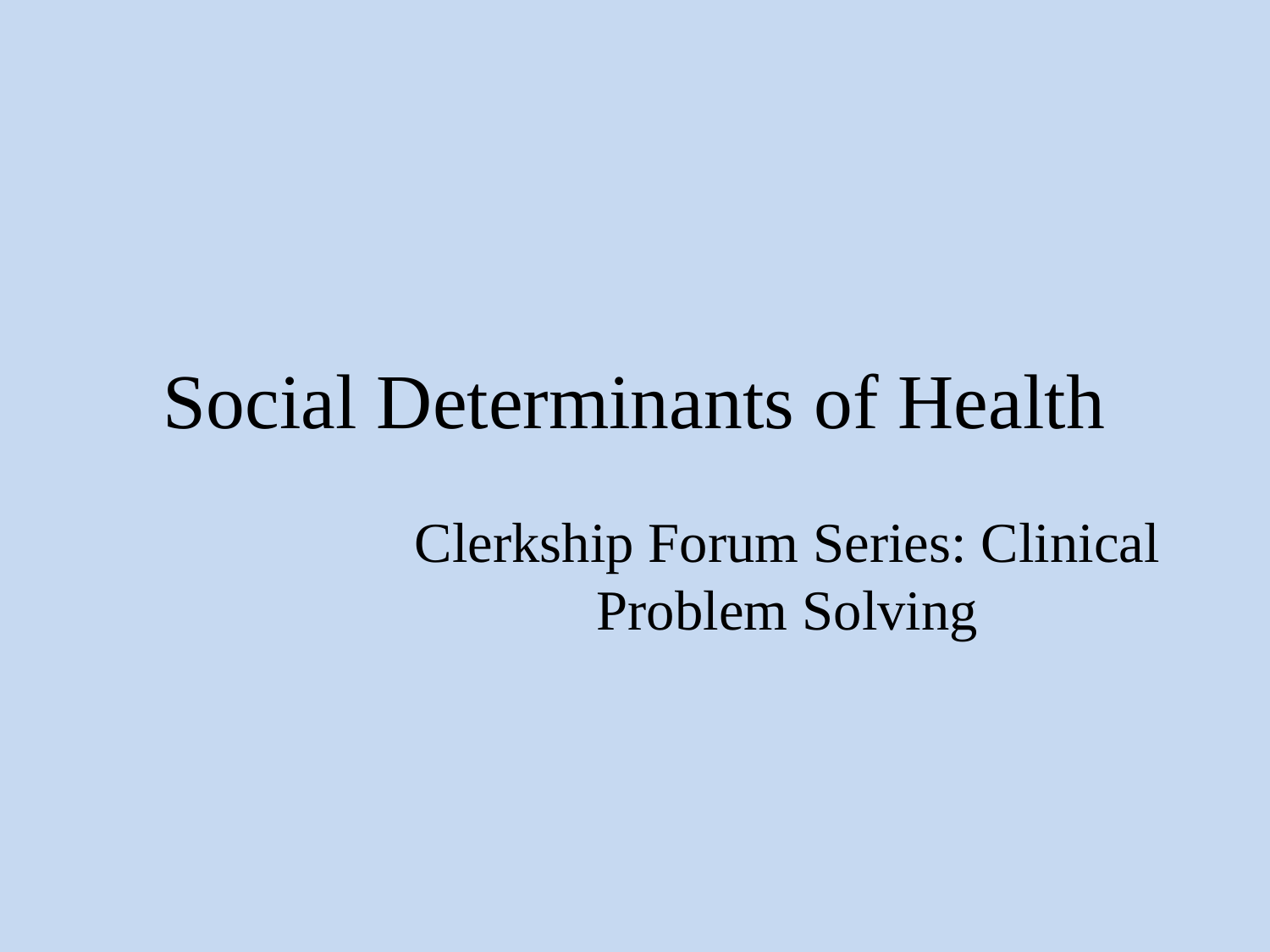

# Social Determinants of Health
Clerkship Forum Series: Clinical Problem Solving

## Slide 2
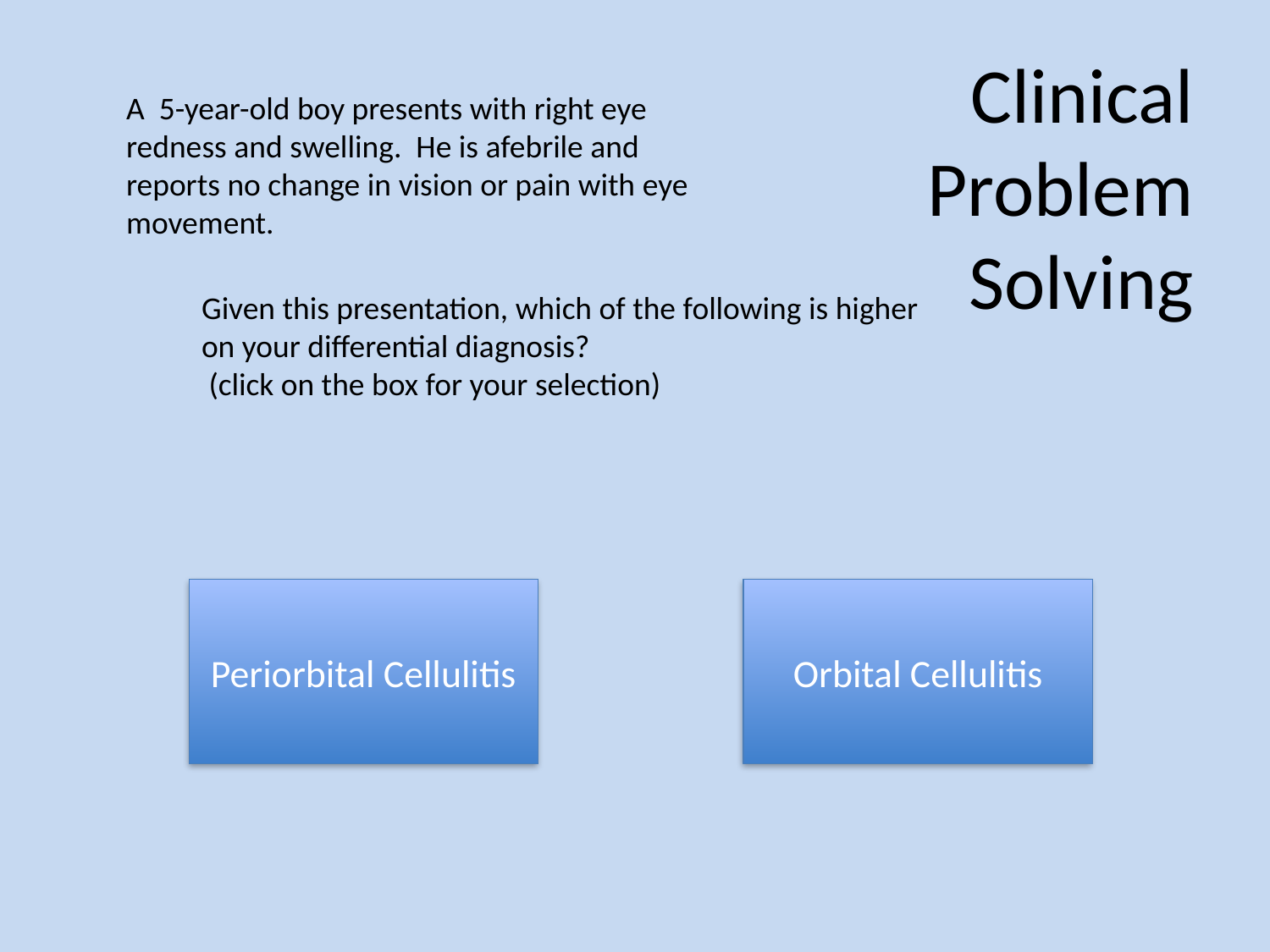

# Clinical Problem Solving
A 5-year-old boy presents with right eye redness and swelling. He is afebrile and reports no change in vision or pain with eye movement.
Given this presentation, which of the following is higher on your differential diagnosis?
 (click on the box for your selection)
Periorbital Cellulitis
Orbital Cellulitis

## Slide 3
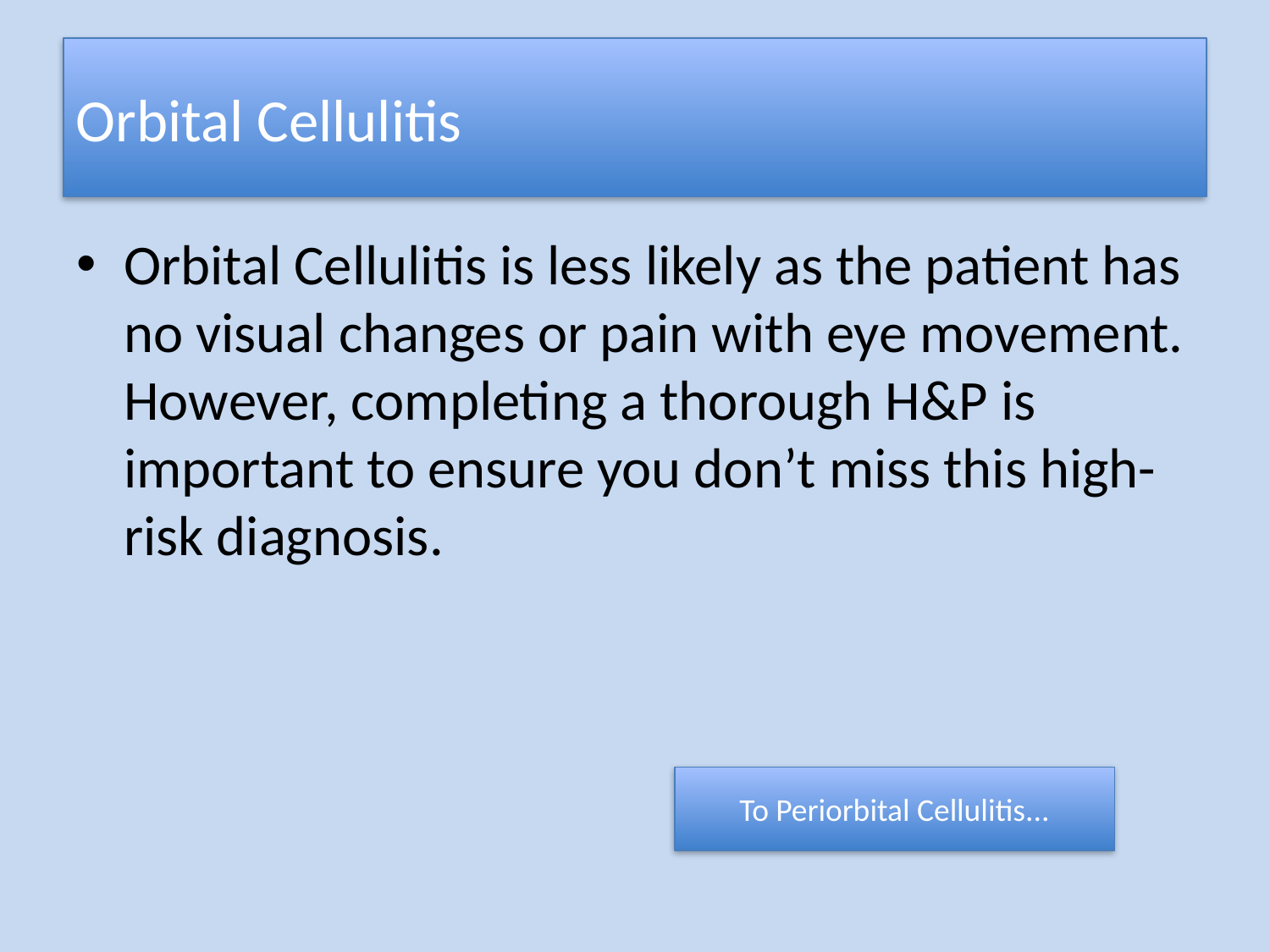

Orbital Cellulitis
Orbital Cellulitis is less likely as the patient has no visual changes or pain with eye movement. However, completing a thorough H&P is important to ensure you don’t miss this high-risk diagnosis.
To Periorbital Cellulitis...

## Slide 4
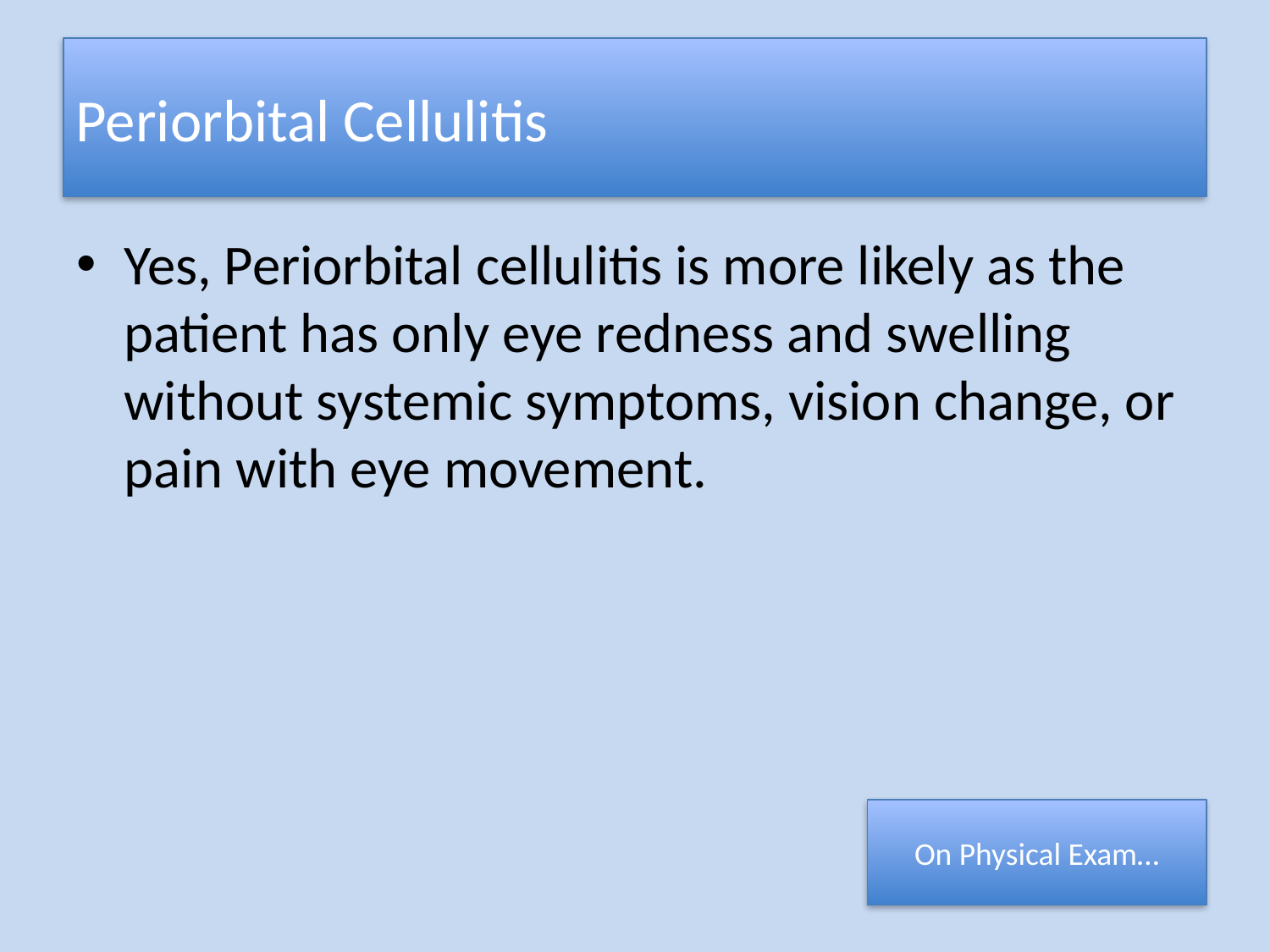

Periorbital Cellulitis
Yes, Periorbital cellulitis is more likely as the patient has only eye redness and swelling without systemic symptoms, vision change, or pain with eye movement.
On Physical Exam…

## Slide 5
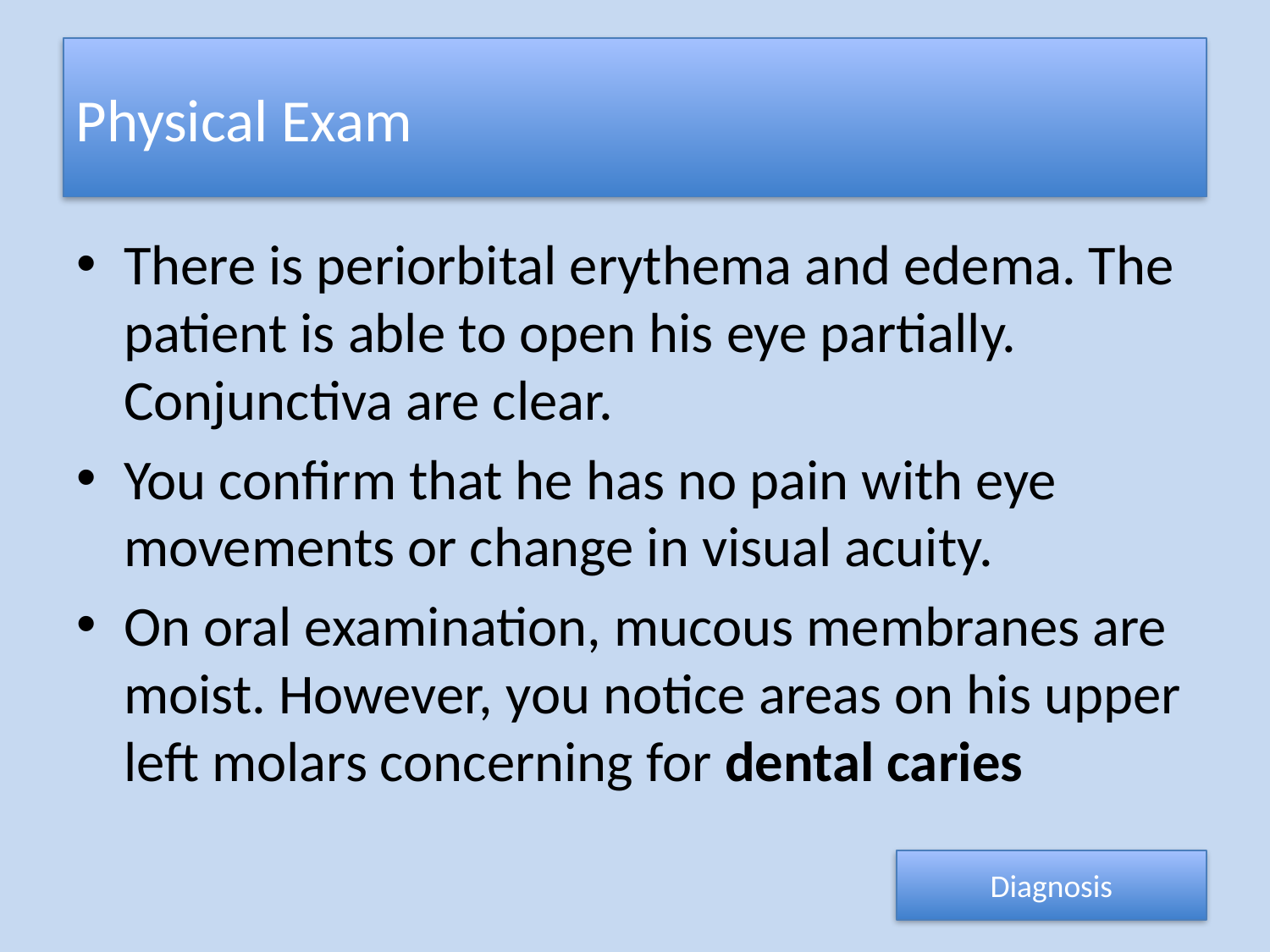

Physical Exam
There is periorbital erythema and edema. The patient is able to open his eye partially. Conjunctiva are clear.
You confirm that he has no pain with eye movements or change in visual acuity.
On oral examination, mucous membranes are moist. However, you notice areas on his upper left molars concerning for dental caries
Diagnosis

## Slide 6
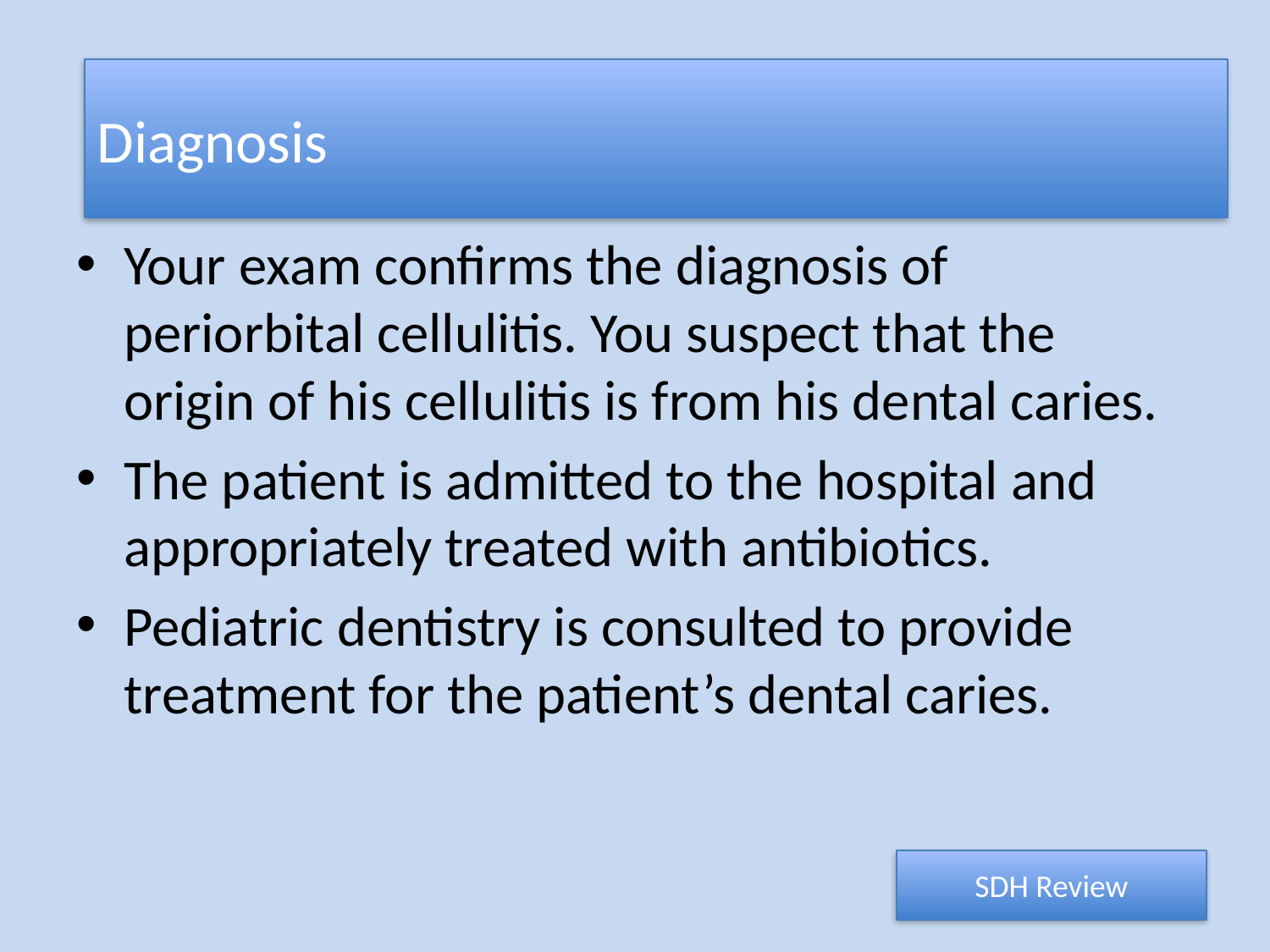

Diagnosis
Your exam confirms the diagnosis of periorbital cellulitis. You suspect that the origin of his cellulitis is from his dental caries.
The patient is admitted to the hospital and appropriately treated with antibiotics.
Pediatric dentistry is consulted to provide treatment for the patient’s dental caries.
SDH Review

## Slide 7
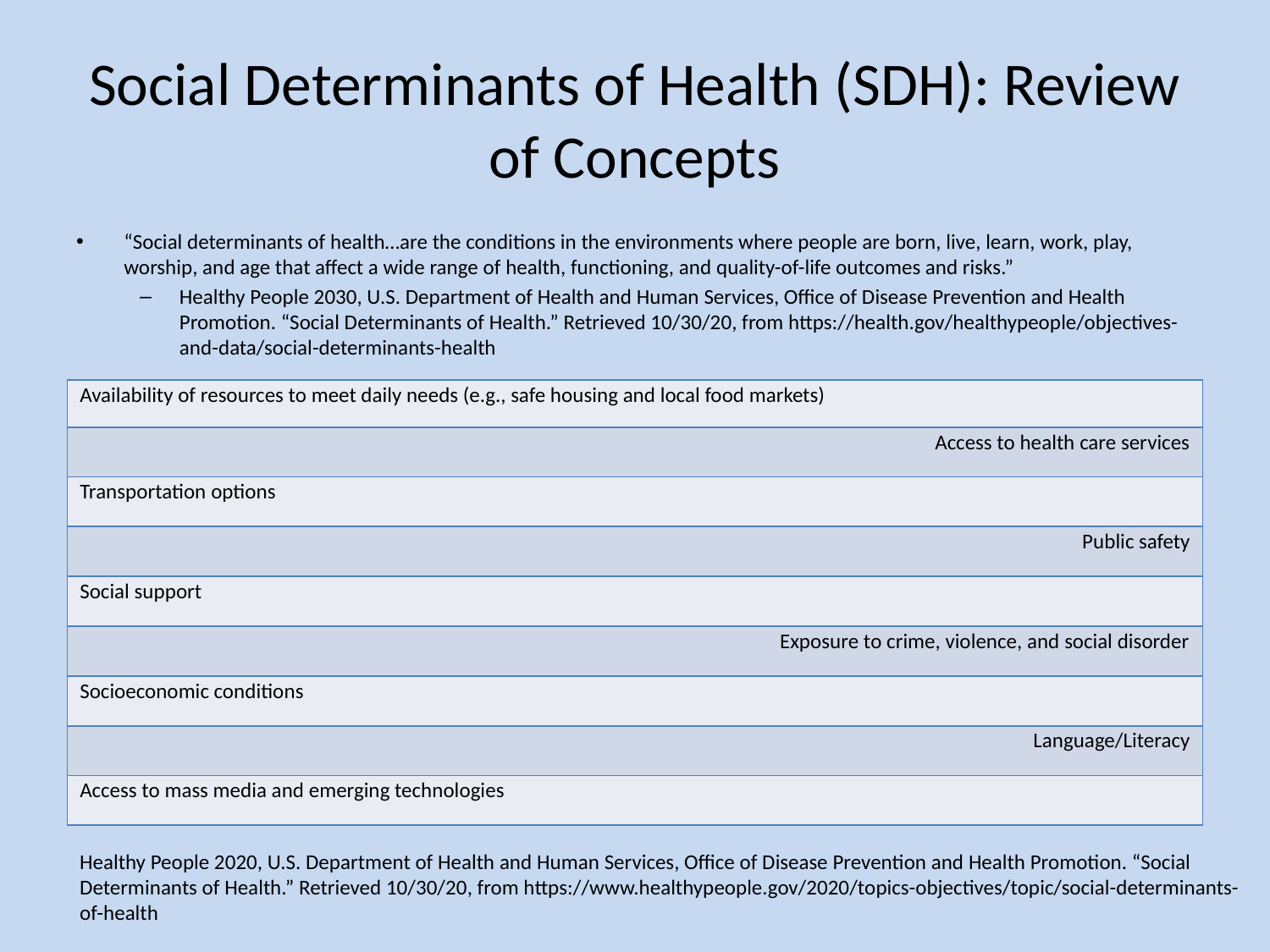

# Social Determinants of Health (SDH): Review of Concepts
“Social determinants of health…are the conditions in the environments where people are born, live, learn, work, play, worship, and age that affect a wide range of health, functioning, and quality-of-life outcomes and risks.”
Healthy People 2030, U.S. Department of Health and Human Services, Office of Disease Prevention and Health Promotion. “Social Determinants of Health.” Retrieved 10/30/20, from https://health.gov/healthypeople/objectives-and-data/social-determinants-health
| Availability of resources to meet daily needs (e.g., safe housing and local food markets) |
| --- |
| Access to health care services |
| Transportation options |
| Public safety |
| Social support |
| Exposure to crime, violence, and social disorder |
| Socioeconomic conditions |
| Language/Literacy |
| Access to mass media and emerging technologies |
Healthy People 2020, U.S. Department of Health and Human Services, Office of Disease Prevention and Health Promotion. “Social Determinants of Health.” Retrieved 10/30/20, from https://www.healthypeople.gov/2020/topics-objectives/topic/social-determinants-of-health

## Slide 8
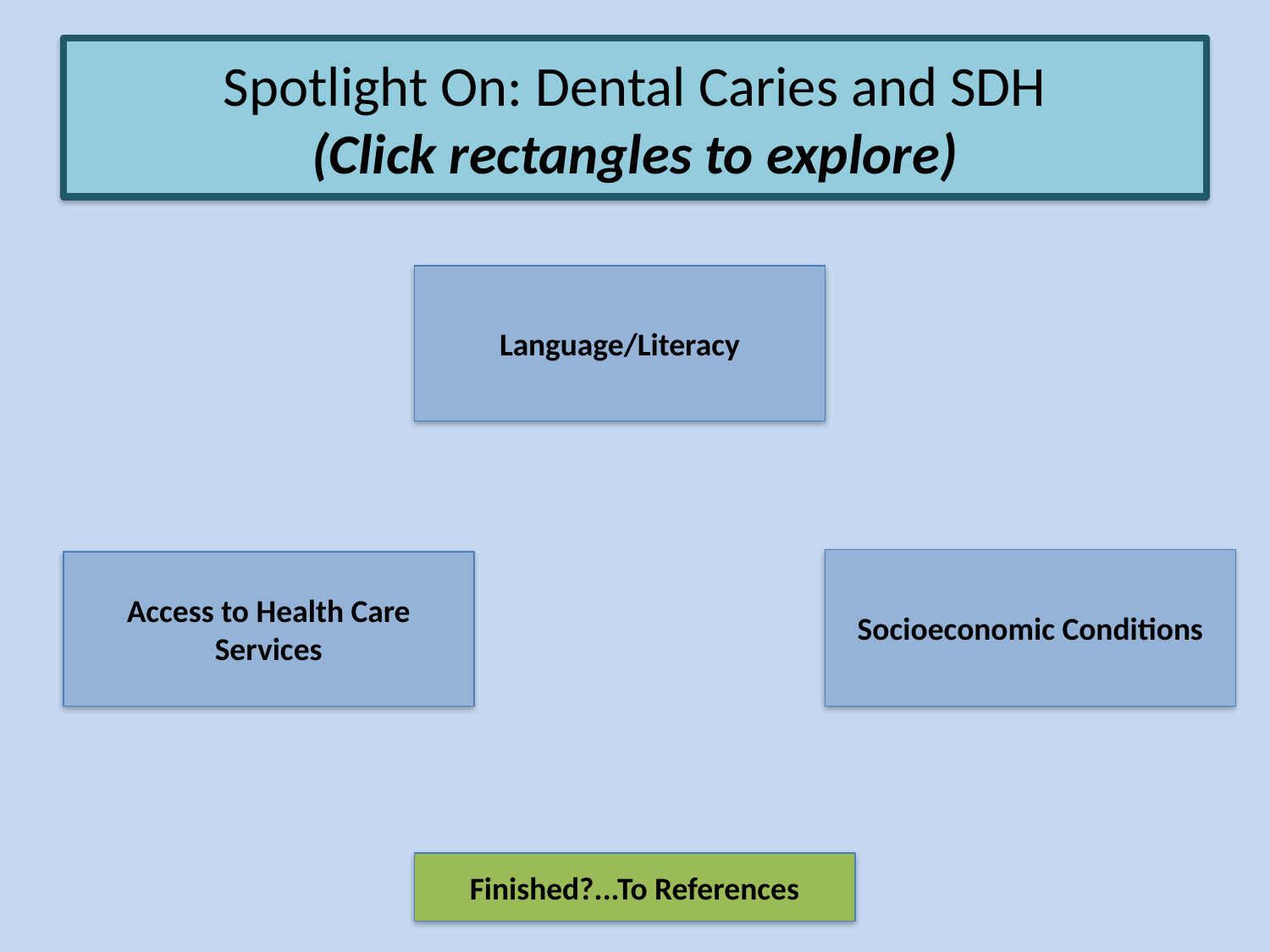

#
Spotlight On: Dental Caries and SDH
(Click rectangles to explore)
Language/Literacy
Socioeconomic Conditions
Access to Health Care Services
Finished?...To References

## Slide 9
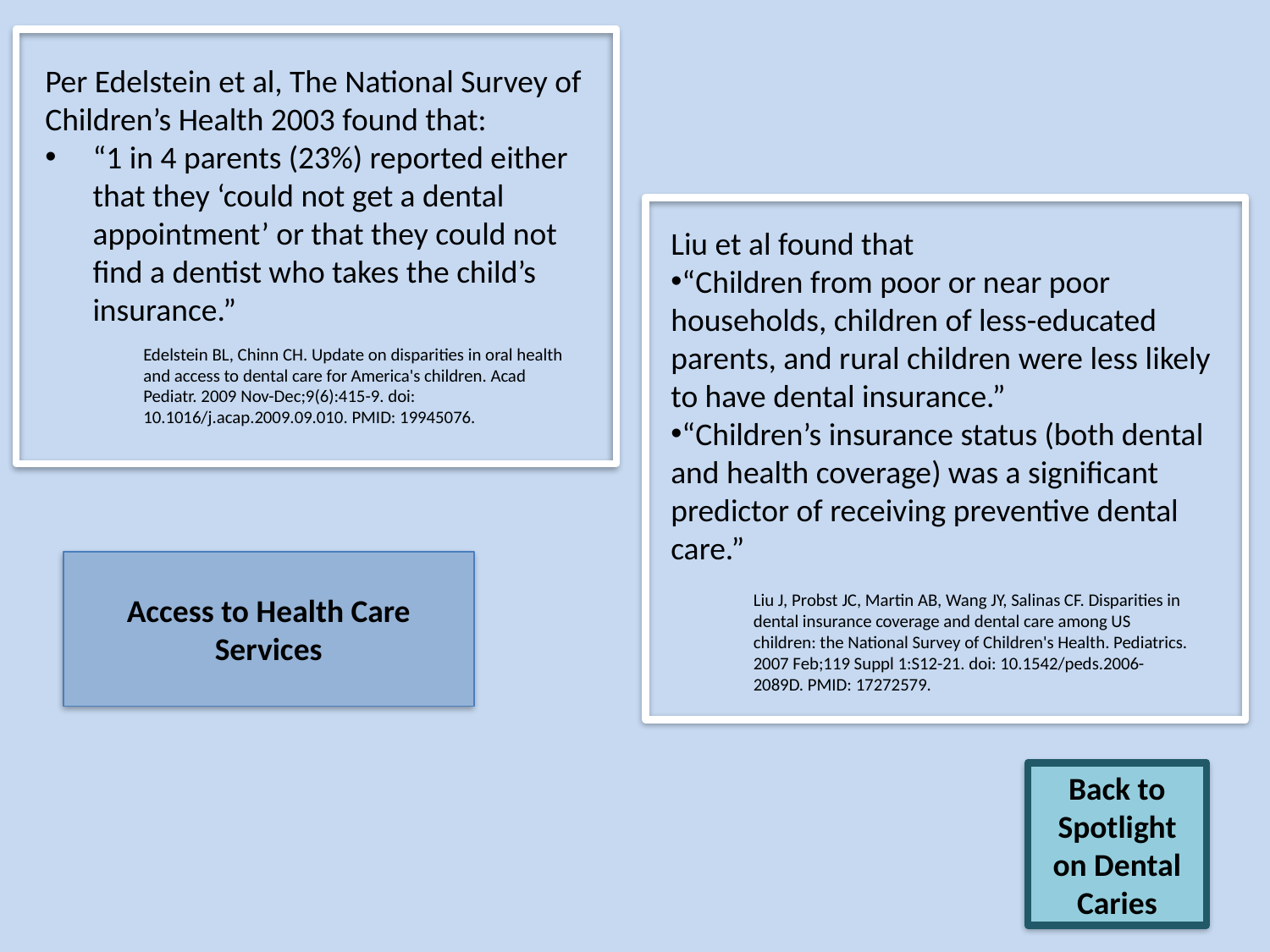

Per Edelstein et al, The National Survey of Children’s Health 2003 found that:
“1 in 4 parents (23%) reported either that they ‘could not get a dental appointment’ or that they could not find a dentist who takes the child’s insurance.”
Liu et al found that
“Children from poor or near poor households, children of less-educated parents, and rural children were less likely to have dental insurance.”
“Children’s insurance status (both dental and health coverage) was a significant predictor of receiving preventive dental care.”
Edelstein BL, Chinn CH. Update on disparities in oral health and access to dental care for America's children. Acad Pediatr. 2009 Nov-Dec;9(6):415-9. doi: 10.1016/j.acap.2009.09.010. PMID: 19945076.
Access to Health Care Services
Liu J, Probst JC, Martin AB, Wang JY, Salinas CF. Disparities in dental insurance coverage and dental care among US children: the National Survey of Children's Health. Pediatrics. 2007 Feb;119 Suppl 1:S12-21. doi: 10.1542/peds.2006-2089D. PMID: 17272579.
Back to Spotlight on Dental Caries

## Slide 10
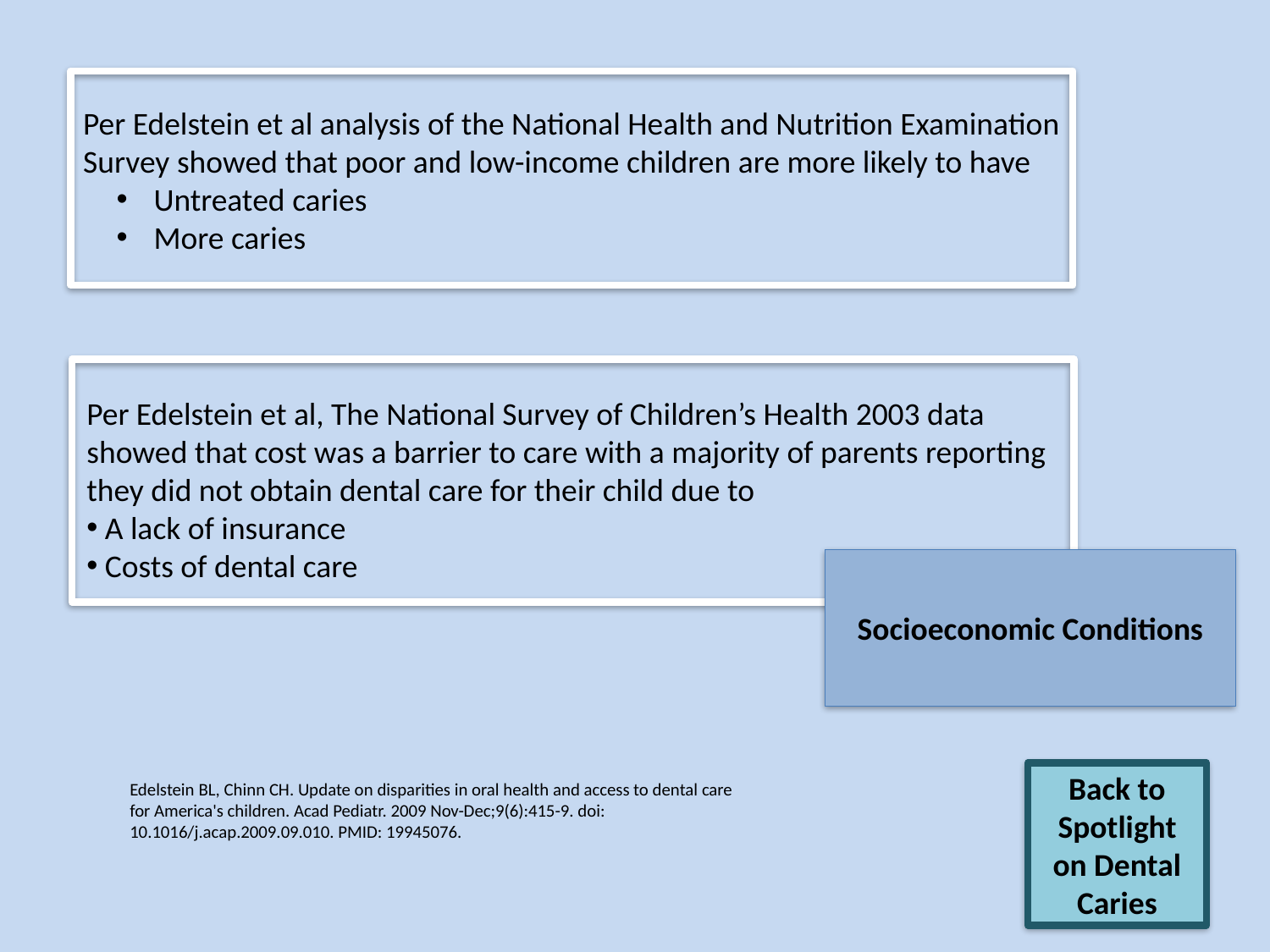

Per Edelstein et al analysis of the National Health and Nutrition Examination Survey showed that poor and low-income children are more likely to have
 Untreated caries
 More caries
Per Edelstein et al, The National Survey of Children’s Health 2003 data showed that cost was a barrier to care with a majority of parents reporting they did not obtain dental care for their child due to
 A lack of insurance
 Costs of dental care
Socioeconomic Conditions
Back to Spotlight on Dental Caries
Edelstein BL, Chinn CH. Update on disparities in oral health and access to dental care for America's children. Acad Pediatr. 2009 Nov-Dec;9(6):415-9. doi: 10.1016/j.acap.2009.09.010. PMID: 19945076.

## Slide 11
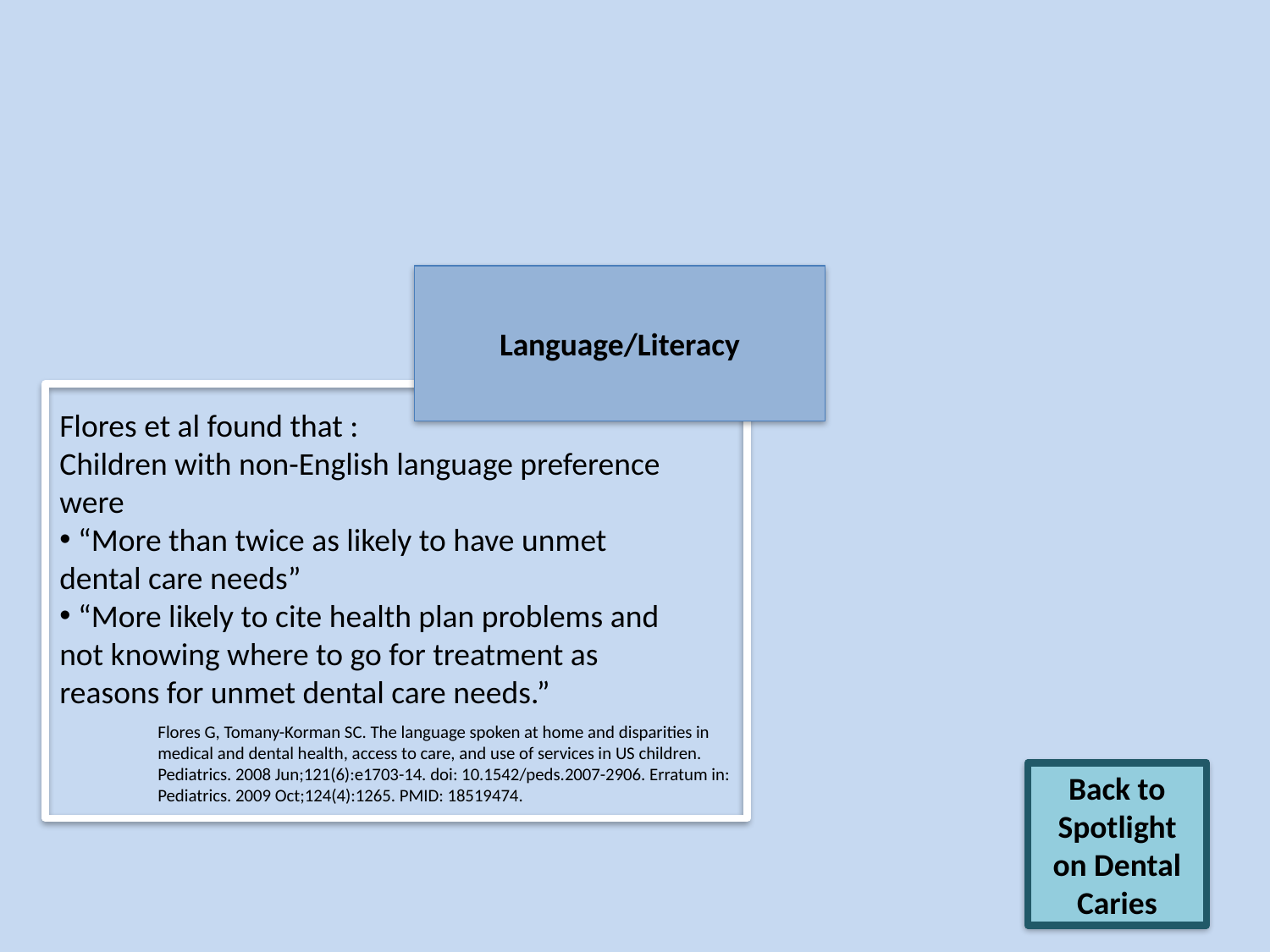

Language/Literacy
Flores et al found that :
Children with non-English language preference were
 “More than twice as likely to have unmet dental care needs”
 “More likely to cite health plan problems and not knowing where to go for treatment as reasons for unmet dental care needs.”
Flores G, Tomany-Korman SC. The language spoken at home and disparities in medical and dental health, access to care, and use of services in US children. Pediatrics. 2008 Jun;121(6):e1703-14. doi: 10.1542/peds.2007-2906. Erratum in: Pediatrics. 2009 Oct;124(4):1265. PMID: 18519474.
Back to Spotlight on Dental Caries

## Slide 12
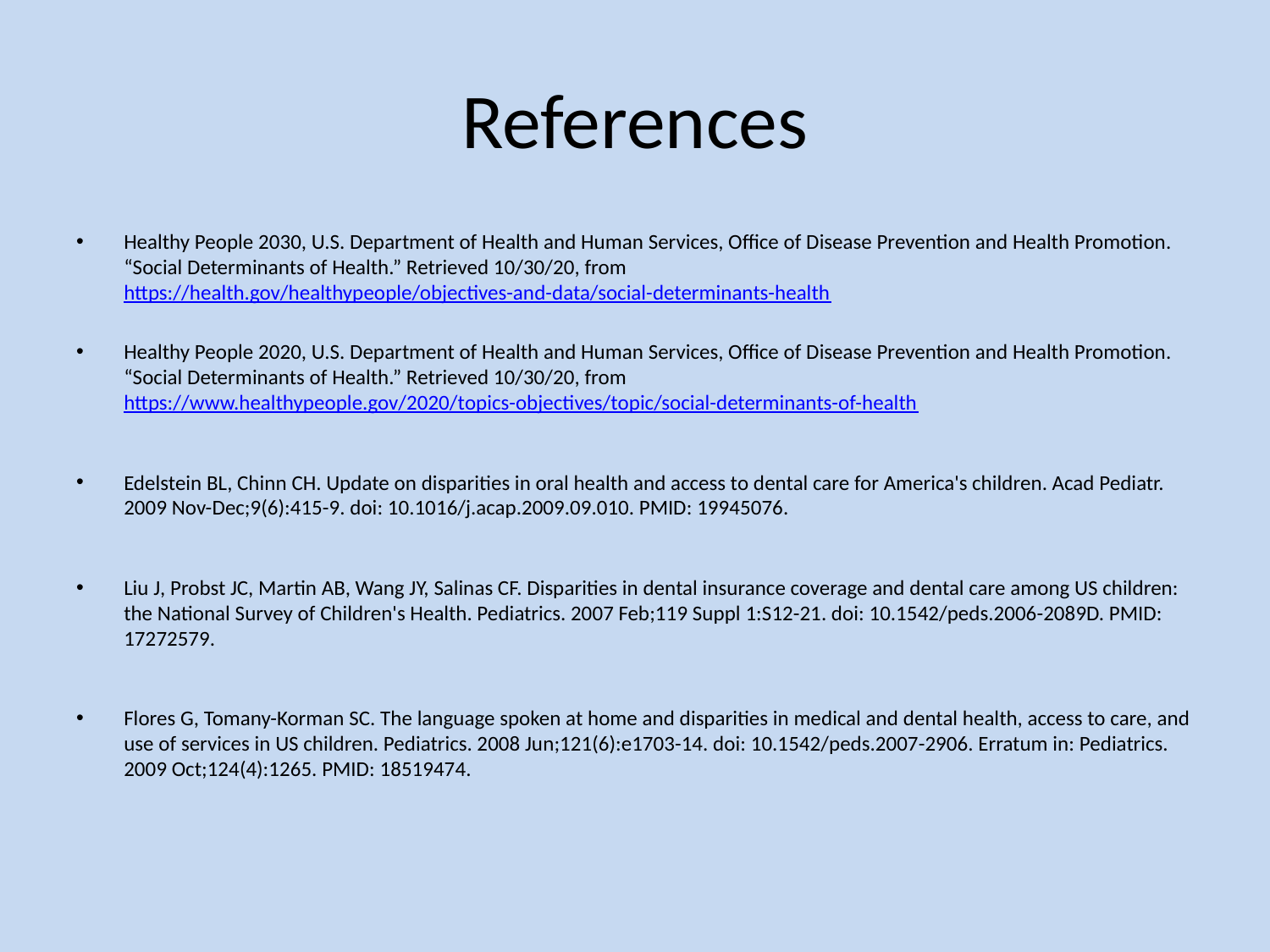

# References
Healthy People 2030, U.S. Department of Health and Human Services, Office of Disease Prevention and Health Promotion. “Social Determinants of Health.” Retrieved 10/30/20, from https://health.gov/healthypeople/objectives-and-data/social-determinants-health
Healthy People 2020, U.S. Department of Health and Human Services, Office of Disease Prevention and Health Promotion. “Social Determinants of Health.” Retrieved 10/30/20, from https://www.healthypeople.gov/2020/topics-objectives/topic/social-determinants-of-health
Edelstein BL, Chinn CH. Update on disparities in oral health and access to dental care for America's children. Acad Pediatr. 2009 Nov-Dec;9(6):415-9. doi: 10.1016/j.acap.2009.09.010. PMID: 19945076.
Liu J, Probst JC, Martin AB, Wang JY, Salinas CF. Disparities in dental insurance coverage and dental care among US children: the National Survey of Children's Health. Pediatrics. 2007 Feb;119 Suppl 1:S12-21. doi: 10.1542/peds.2006-2089D. PMID: 17272579.
Flores G, Tomany-Korman SC. The language spoken at home and disparities in medical and dental health, access to care, and use of services in US children. Pediatrics. 2008 Jun;121(6):e1703-14. doi: 10.1542/peds.2007-2906. Erratum in: Pediatrics. 2009 Oct;124(4):1265. PMID: 18519474.
