# Supplementary material for: Social Determinants of Health Curriculum for the Pediatric Clerkship
Source: MedEdPORTAL. 2024 Oct 29;20:11458. doi: 10.15766/mep_2374-8265.11458 (PMC11518917; doi:10.15766/mep_2374-8265.11458)
Supplement: Supplementary file 1 — SDH Cases Faculty Supplements.docxCurriculum Orientation.pptxSDH Cases Student Handouts.docxPrework - Well Child.pptxPrework - Urgent Care.pptxPrework - Clinical Problem-solving.pptxPrework - Chronic Illness.pptxResource Assignment Orientation.pptxResource Assignment Form and Example.docxFacilitator Reminder Email.docxPresurvey and Case Analysis.docxPostsurvey and Case Analysis.docxCase Analysis Scoring Tool.docx [file mep_2374-8265.11458-s001.zip › _Educational Summary Report_11458.pdf]

MedEdPORTAL® The AAMC Journal of Teaching and Learning Resources

Original Publication Open Access

Social Determinants of Health Curriculum for the Pediatric Clerkship

Caroline Roth, MD*, Amy Prudhomme, DO, Kayla Griese, DO, Robbie Beyl, PhD, Jessica Patrick-Esteve, MD

*Corresponding author: croth1@lsuhsc.edu

Abstract

Introduction: Education on social determinants of health (SDH) aligns with national standards for medical education. However, there are minimal existing resources targeted to medical students specific to the care of the pediatric population. We designed a case-based curriculum on SDH for third-year medical students on their pediatric clerkship to address this deficit. **Methods:** Third-year medical students on their pediatric clerkship received a case-based flipped classroom educational series on SDH in four 10-minute segments. Students completed voluntary and anonymous surveys delivered via an electronic survey tool before and after completion of the curriculum. Surveys were a self-assessment of knowledge and skills related to SDH in pediatrics and analysis of a pediatric case. **Results:** One hundred sixty-seven third-year medical students completed the curriculum during their pediatric clerkship. Pre- and postsurvey response rates were 50% and 39%, respectively, for the self-assessment and 51% and 38%, respectively, for the case analysis components of the survey. Students demonstrated statistically significant improvement in knowledge and skills regarding SDH. After completion of the curriculum, students were more likely to identify SDH as factors contributing to the patient's health status and to propose questions targeted at SDH they would ask the patient or family when presented with a pediatric case. **Discussion:** A case-based curriculum on SDH using a multisession flipped classroom approach advanced student knowledge and skills regarding SDH in the pediatric context. The curriculum has the potential for expansion to other institutions and to serve as a model for other subspecialties.

Keywords
Pediatric Clerkship, Case-Based Learning, Flipped Classroom, Health Equity, Pediatrics, Social Determinants of Health

Educational Objectives

By the end of this activity, learners will be able to:

1. Identify common social determinants of health affecting pediatric patients.
2. Analyze a pediatric case for social determinants of health that may affect patient health.
3. Construct questions to elicit social determinants of health for a pediatric clinical encounter.
4. Recognize resources to help mitigate effects of social determinants of health for pediatric patients.

Introduction

The focus on social determinants of health (SDH) has continued to increase over the last few decades. Addressing SDH is a priority for some of the most impactful national and international health organizations, including the Office of Disease Prevention and Health Promotion (ODPHP) and the World Health Organization (WHO).[1,2] The Commission on Social Determinants of Health, established in 2005 by the WHO, recommended establishing governmental involvement in monitoring and addressing SDH as a means of achieving health equity.[2] In the United States of America, every decade the ODPHP releases a new iteration of the Healthy People initiative, outlining goals and objectives for public health priorities.[3] A focus on SDH was added to this initiative in the 2020 iteration and reinforced for the 2030 objectives.[1,3,4] Professional societies, educational communities, and health care organizations are important stakeholders in addressing the WHO and Healthy People goals regarding SDH. In 2013, the American Academy of Pediatrics released the policy statement "Community Pediatrics: Navigating the Intersection of Medicine, Public Health, and Social Determinants of Children's Health," which recommends incorporation of education on SDH into pediatric medical curricula.[5] The 2019 Council on Medical Student Education in Pediatrics (COMSEP) curriculum revision incorporated education on SDH, including the objective that "by the end of the pediatric

Citation:
Roth C, Prudhomme A, Griese K, Beyl R, Patrick-Esteve J. Social determinants of health curriculum for the pediatric clerkship. *MedEdPORTAL*. 2024;20:11458.
https://doi.org/10.15766/mep_2374-8265.11458

Copyright © 2024 Roth et al. This is an open-access publication distributed under the terms of the Creative Commons Attribution license.

clerkship a student will… recognize the role of culture, values, beliefs, and social determinants of health in influencing health and illness."[6]

In accordance with Kern's six-step approach to curriculum development,[7] a needs assessment of medical students and key faculty involved in pediatric clerkship didactics at our institution was conducted; it identified inconsistencies in education on SDH during the pediatric clerkship. Opportunities were identified to advance student knowledge about SDH for pediatric-specific situations. Faculty comfort in providing education on SDH was found to be variable.

A review of literature focused on SDH education resources suggests that case studies, immersive experiences such as home visits or activities in the community, and longitudinal curricula improve learner knowledge and skills regarding SDH.[8-21] However, many of these resources are designed for the preclinical years.[8-11] Among the published resources on SDH education in the clinical and postclinical years, the majority require home visits or are primarily focused on adult patients, limiting their applicability to students in the pediatric clerkship.[13,14,16-18] Given the importance of understanding SDH to strive for health equity in current and future patient care, it is important that all students have access to this education throughout their training. Education on SDH should mirror that of other clinical curricula, with expansion and application of the knowledge past the preclinical years and integration of pediatric content. For pediatric-specific resources, Marsh and colleagues developed a single-session lecture to introduce medical students to SDH with a focus on the relationship between SDH and advocacy.[21] Klein and colleagues produced a curriculum on screening for SDH aimed at pediatric residents.[15] We aimed to expand on the work done by these authors by creating a multisession curriculum with case-based modules directed at the level of a third-year medical student and evaluated via a novel mixed methods approach.

Methods

Curricular Context

We designed a curriculum for third-year medical students on their pediatric clerkship that was first introduced in January 2021. We selected this time period for feasibility reasons based upon GME scholarly timeline requirements for the primary author. The curriculum required no prerequisite knowledge for either learners or facilitators. All students on their third-year pediatric clerkship in 2021 received this curriculum and had the opportunity to complete assessment tools for its evaluation. There were no exclusionary criteria for this population.

We created the curricular materials to align with the existing didactic format for our institution's pediatric clerkship, which included four longitudinal small-group didactic sessions composed of six to eight students and led by a pediatric faculty member. The students and faculty member were consistent among all four sessions. Faculty came from a variety of subspecialties and led one to two groups per year. Most of the small-group facilitators were clinical educators without additional training or leadership experience in medical education. The four small-group didactic sessions were themed Well Child, Urgent Care, Clinical Problem-solving, and Chronic Illness. Activities within these small groups included cases and quiz games relevant to each theme. We created the material for the SDH curriculum to correspond with the small-group themes while maintaining the ability to function as a stand-alone curriculum.

The project was reviewed by the Louisiana State University Health Sciences Center New Orleans Institutional Review Board (IRB) and approved as exempt research.

Curricular Design

Curriculum design was informed by a team member with a background in education in collaboration with local medical education leaders. We based our curriculum design on Ericsson's theory of deliberate practice,[22] aiming to give students multiple opportunities to practice knowledge and skills related to SDH in the pediatric context with immediate faculty feedback and reinforcement.

Our institution used a flipped classroom model for pediatric didactic sessions, and we designed the curriculum to be incorporated into this model. We chose the knowledge and skills regarding SDH for pediatric patients to mirror those students would utilize in the clinical context. Targeted skills included crafting questions to identify and elicit potential SDH for a pediatric patient. Providing students opportunities to practice skills in a small-group setting using theoretical patient cases allowed for reduced cognitive load and direct faculty observation and feedback. Given the importance of not only identifying but also addressing SDH for pediatric patients, a resource assignment was also incorporated into the curriculum to increase students' basic knowledge of local resources for our patient population.

Supplemental resources for faculty were also developed to support faculty comfort with providing education on SDH (Appendix A).

We piloted the curriculum in 2020 with a small group led by the pediatric clerkship director. Modifications were made to

Copyright © 2024 Roth et al. This is an open-access publication distributed under the terms of the Creative Commons Attribution license.

the curriculum based on feedback from these students and the clerkship director.

Curriculum Details

The curriculum utilized four modules that paired prework material with small-group case discussions. Additionally, a resource assignment was due during the final small group. The clerkship director introduced the curriculum during the pediatric clerkship orientation session (Appendix B).

Prework consisted of one interactive PowerPoint per session, accessible via the online learning platform Moodle, that was completed by students prior to the relevant small group. Each prework PowerPoint began with a pediatric case scenario and then covered evidence from the literature on SDH relevant to a common pediatric diagnosis tied to that scenario. The first prework PowerPoint introduced the definition and examples of SDH per the ODPHP Healthy People website. The remaining prework PowerPoint presentations provided a brief review of these topics. The first prework PowerPoint also concluded by guiding students through analysis and question formation to identify and elicit SDH for a pediatric case in anticipation of the students requiring these skills during their small-group sessions.

Each small-group session included a single pediatric case scenario, supplied via a handout, for which the facilitator led a discussion regarding SDH utilizing provided prompting questions (Appendix C). In the first two small groups, students were asked:

- “What social determinants of health might be a factor in this scenario?”
- “What kinds of questions could you ask the patient’s family to discover what social determinants of health might be affecting him/her?”

In the final two small groups, students were asked:

- “What is your leading diagnosis?”
- “What are some factors you think could be contributing to his/her health status?”
- “What additional questions would you like to ask the patient and his/her family?”

We designed the evolution of the questions throughout the four sessions to help the students’ thought process transition to consideration of SDH for all patients and scenarios rather than only when asked directly about SDH. We recommended that facilitators dedicate 5-10 minutes to the case discussions. The format of case discussion was flexible and left to the individual facilitators to allow for potential time constraints.

Below is an outline of the modules and topics that students completed:

- Module 1: Well Child
  - Prework: interactive PowerPoint (Appendix D) covering the following:
    - Definition of SDH
    - Examples of categories of SDH
    - SDH and obesity
    - Example case: atopic dermatitis
  - Small group: failure to thrive case handout (Appendix C)
- Module 2: Urgent Care
  - Prework: interactive PowerPoint (Appendix E) covering SDH and asthma
  - Small group: intentional ingestion case handout (Appendix C)
- Module 3: Clinical Problem-solving
  - Prework: interactive PowerPoint (Appendix F) covering SDH and dental caries
  - Small group: status epilepticus case handout (Appendix C) with a reminder that the resource assignment was due at the next small group
- Module 4: Chronic Illness
  - Prework: interactive PowerPoint (Appendix G) covering SDH and diabetes mellitus
  - Small group:
    - Splenic sequestration case handout (Appendix C)
    - Resource assignment presentation

Resource Assignment

Students were introduced to the resource assignment during their orientation via a PowerPoint slide (Appendix H) and were also provided with a hard copy of the assignment form and the example (Appendix I), which were additionally posted on their online learning platform.

Students were asked to research a locally accessible resource that could be offered to a pediatric patient to help mitigate SDH. Students were provided with a list of resources but also given the option to select their own resource if desired. For the resource assignment, students completed and submitted a form with identification of the categories of SDH the resource addressed, a one- to three-sentence description of the resource, and a one- to three-sentence explanation of how this resource could benefit a pediatric patient. Students provided this information to their peers during their final small-group sessions via 1-minute presentations.

Copyright © 2024 Roth et al. This is an open-access publication distributed under the terms of the Creative Commons Attribution license.

Facilitator Support
Via hard copy and email, we provided facilitators with a facilitator guide (Appendix A) that outlined the curricular components and contained potential answers to the discussion questions posed for each small-group case. We also sent an email (Appendix J) prior to the final small group with a reminder that the resource assignment presentation should occur during that small group and requesting that the students be allotted time to complete the online postsurvey for the curriculum.

Evaluation Strategy
To evaluate the curriculum, students were invited to complete a voluntary pre- and postsurvey and case analysis (Appendices K and L). Pre- and postsurveys assessed student knowledge and skills for the curricular objectives and the value of learning about SDH for their medical education. The surveys utilized a 7-point Likert-type scale with possible answers ranging from *Strongly Disagree* to *Strongly Agree.* This range was chosen with the hope that it might provide more nuanced responses than a scale with fewer options. Students also reviewed a pediatric case, then had to propose a leading diagnosis, name factors that could be contributing to the health status, and propose additional questions they would like to ask the patient and family. Additionally, the postsurvey assessed students' perception of whether the modules advanced their understanding of SDH, asked them to quantify the frequency with which SDH was discussed in both the small-group sessions and clinical context, and included an option to provide further comments. The mixed methods approach of these evaluation tools was chosen to target levels 1 and 2 of the Kirkpatrick model.[23]

The presurvey was sent to students anonymously via an online survey tool prior to their pediatric clerkship orientation. Students were allotted time during their clerkship orientation to voluntarily complete the survey. The postsurvey was emailed to students prior to their final small group with the instructions to complete the survey during or after the small group. Facilitators were asked to provide 5-10 minutes during the final small group to allow for survey completion. For orientation sessions or small groups that experienced time constraints prohibiting dedicated survey completion time, a reminder email was sent to students about the survey.

Analysis
Data were collected from January 2021 through December 2021. This sampling period included four blocks of students.

Pre- and postsurvey data were analyzed in terms of proportion of student responses on the 7-point Likert-type scale ranging from *Strongly Disagree* to *Strongly Agree*. The categories of *Strongly Disagree, Disagree, Somewhat Disagree*, and *Neither Agree nor Disagree* were combined due to small sample sizes.

Pre- and postsurvey responses for the pediatric case analysis were qualitatively evaluated in terms of total number of items the students posed related to SDH for the prompts "Please name at least four factors which you think could be contributing to her health status" and "What are at least four additional questions you would like to ask the patient and her family?" Responses that fell into the domains established by Healthy People 2030—Economic Stability, Education Access and Quality, Health Care Access and Quality, Neighborhood and Built Environment, and Social and Community[1]—were coded as SDH-related responses (Appendix M). Two team members coded the initial responses (Kayla Griese and Caroline Roth), and a third team member (Amy Prudhomme) coded responses for any discrepancies. For the case analysis, student responses were scored on percentage of responses attributed to SDH for both identified factors and proposed questions.

All data were considered as categorical in nature due to the limited possible outcomes. Data were anonymous and thus unable to be matched; however, statistical tests used all available data for each outcome. All results were based on chi-square tests to determine associations between groups, with results presented as percentages. No covariates were used in modeling due to the anonymous nature of data with no demographic related variables being collected. We considered p values less than .05 statistically significant.

Results

A total of 167 third-year medical students received the curriculum during their required pediatric core clerkship. Student response rates for the pre- and postsurvey were 50% (83) and 39% (65), respectively, and for the precurriculum and postcurriculum case analysis were 51% (86) and 38% (64), respectively.

Students showed statistically significant improvement regarding self-reported abilities of defining SDH, identifying SDH commonly affecting pediatric patients, analyzing a case for SDH that might be affecting a pediatric patient, forming questions to elicit SDH for pediatric patients, and identifying resources to help mitigate effects of SDH for pediatric patients (Table).

The greatest area of improvement was in identification of resources, with only 13% of students agreeing or strongly agreeing that they "can identify resources to help mitigate effects of SDH for pediatric patients" prior to the curriculum and 75%

Copyright © 2024 Roth et al. This is an open-access publication distributed under the terms of the Creative Commons Attribution license.

Table. Percentage of Student Responses Related to Knowledge, Skills, and Attitudes Before and After Completion of the SDH Curriculum

	% Strongly Disagree, Disagree, Somewhat Disagree, or Neither Agree nor Disagree		% Somewhat Agree		% Agree		% Strongly Agree		
**Statement**	**Pre**	**Post**	**Pre**	**Post**	**Pre**	**Post**	**Pre**	**Post**	***p***
I can accurately define the concept of SDH.	13	6	29	5	45	32	13	57	<.001[a]
I am confident in identifying SDH that commonly affect pediatric patients.	28	6	46	6	22	38	5	49	<.001[a]
I am confident in analyzing a pediatric case for SDH that may be affecting the patient.	25	6	40	5	29	35	6	54	<.001[a]
I feel comfortable forming questions to elicit SDH for a pediatric patient.	41	6	35	9	19	43	5	42	<.001[a]
I can identify resources to help mitigate effects of SDH for pediatric patients.	60	8	26	17	11	35	2	40	<.001[a]
Learning about SDH is valuable to my medical education.	4	6	10	3	23	25	64	66	.41
Pediatric forums advanced my understanding of SDH.		8		8		38		46	

Abbreviation: SDH, social determinants of health.
[a]Statistically significant at $p < .05$.

of students agreeing or strongly agreeing with this statement after completion of the curriculum. In addition, 85% of students agreed or strongly agreed that the small groups advanced their understanding of SDH (Table).

There was statistical significance ($p < .001$) in the ability of the students both to identify factors (Figure 1) and to pose more questions (Figure 2) related to SDH when presented with a pediatric case scenario after completion of the curriculum.

The third-year medical students who participated in curricular evaluation represented two separate medical school class years as data collection spanned January-December 2021. However, there were no statistical differences in any of the responses when separated by class year or by time of year.

Discussion

We created and implemented a multisession, flipped classroom, case-based curriculum to address the gap in education on SDH related to pediatrics in the clinical years by targeting medical students on their pediatric core clerkship. Our novel curriculum is in alignment with the national goals of the COMSEP curriculum recommendations. Our intervention resulted in improvement in knowledge and skills related to our educational objectives. Students also demonstrated increased identification of SDH as contributing to a patient's health status in their analysis of a pediatric case. They were able to pose more questions regarding SDH to a theoretical patient and their family.

The curriculum is designed to be generalizable to other institutions as the cases presented are not geographically specific and local resources for other regions could be easily substituted into the resource assignment. Organizations and programs to consider for options for the resource assignment include those that focus on youth development, access to healthy foods, community outreach, support services for survivors of violence and abuse, access to health services, and affordable housing. Because the small-group cases require only 5-10 minutes, they were easily incorporated into our existing didactic structure and lend themselves to similar adoption in other pediatric clerkships where they could be added to any existing didactic sessions. While we did not evaluate the curricular components independently, clerkships with time or other logistical constraints could consider adopting only one or two of the modules, using only the prework or small-group components, combining all four modules, or utilizing only the resource assignment. Future work would be required to ensure students continue to demonstrate improvement in knowledge and skills with these adaptations.

While we evaluated the curriculum exclusively in the core pediatric clerkship for third-year medical students, it could be expanded or adapted for use on any clinical pediatric rotation involving students in health care professions. The multisession flipped classroom format could also be used as a template for other specialties to develop similar curricula.

The third-year medical students participating in the curriculum represented two separate medical student class years and thus may have received variation in preclerkship curricula on related topics. However, the absence of statistically significant differences in the results by student class year suggests that our curriculum could be utilized to advance student knowledge and skills for our objectives regardless of previously provided undergraduate medical education on SDH.

There were minimal barriers to curriculum development as case scenarios were simple to compose in relation to topics and themes previously established for pediatric clerkship education by COMSEP. The information from the Healthy People

Copyright © 2024 Roth et al. This is an open-access publication distributed under the terms of the Creative Commons Attribution license.

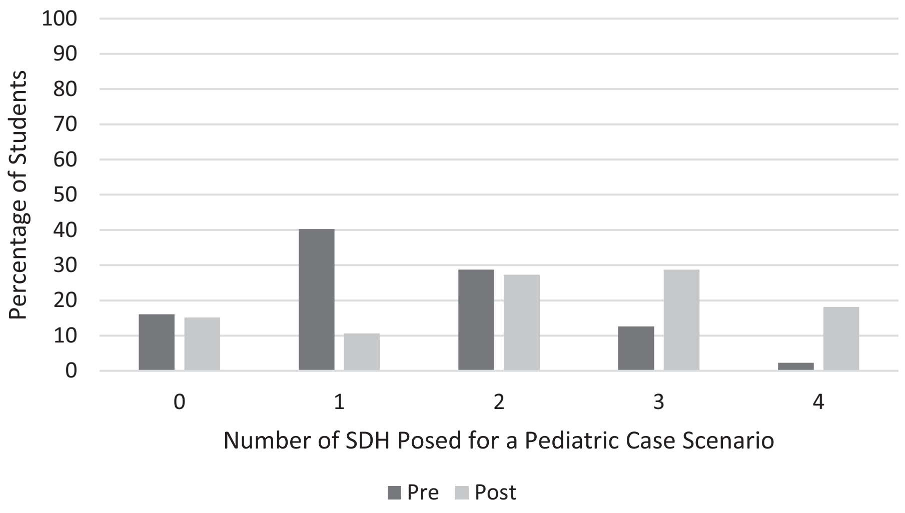

Figure 1. Percentage of students identifying zero to four factors related to social determinants of health (SDH) in their analysis of a pediatric case scenario before and after completion of the SDH curriculum.

initiative also provided a framework on which to base our curriculum.

On the postsurvey, 97% of students reported that they discussed SDH in their small group either four to five times or six or more times. This high percentage suggests that the curriculum was being consistently implemented by our faculty over the course of the four small groups and that students recognized the curricular components focused on SDH.

Important limitations to the curriculum and its evaluation include student response rates, absence of a control group, unmatched data, lack of demographic data, unknown influence of prior education on the topics, and other challenges to validity and generalizability, including implementation and evaluation at a single academic center.

One of the greatest barriers to the project was obtaining student survey responses, which we attempted to address by providing dedicated time in which students could complete the voluntary survey. Mandatory participation in the evaluation of the curriculum could have mitigated response rate bias but was not feasible based on clerkship and IRB policy due to the timing of our curriculum evaluation. A paper survey may have resulted in higher response rates by eliminating the need for electronic access for completion. There may also have been recall bias for

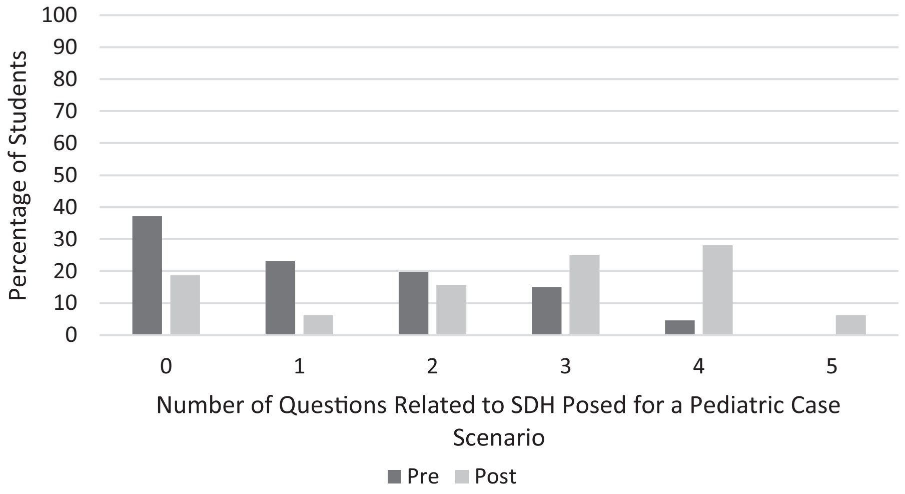

Figure 2. Percentage of students posing zero to five questions related to social determinants of health (SDH) in their analysis of a pediatric case scenario before and after completion of the SDH curriculum.

Copyright © 2024 Roth et al. This is an open-access publication distributed under the terms of the Creative Commons Attribution license.

responses as we did not track which students completed the survey immediately on completion of the curriculum or at a later time, which could also have been addressed by use of a paper survey tool.

The design was quasi-experimental, with a pre- and posttest group receiving the intervention without a control. As a result, some of the knowledge and skills students gained may have resulted from other activities independent of our curriculum.

Another limitation is the lack of matching for pre-post data as survey responses were anonymous. We had hoped to pair data using student-selected anonymous unique participant identifiers. However, the identifiers provided on the postsurvey consistently did not match presurvey identifiers or the suggested format for anonymous identifiers. During this time period, our students were participating in a separate unrelated medical education project that had different unique identifier format recommendations, which likely led to the discrepancy in postsurvey matched identifiers. As a result, we were unable to perform additional statistical analysis such as a paired *t* test that matched data would have allowed. A retrospective pre-post survey for student self-reported abilities could have better allowed for matching, in addition to providing other benefits, including reduced response shift bias. However, this format would not have been appropriate for the case analysis component of evaluation.

We did not collect demographic information on students, small-group assignment details, or facilitator names to help protect student anonymity. Therefore, we were unable to compare survey responders to nonresponders to determine if they differed in characteristics or to perform multivariable analysis by small group or facilitator.

The proportion of students agreeing or strongly agreeing that “learning about social determinants of health is valuable to my medical education” increased from 87% to 91%, but this change was not statistically significant. The students at our institution received education regarding SDH in their first and second years as part of a longitudinal health equity course. Our findings suggest that the students’ prior exposure to SDH through this curriculum and other experiences may have already resulted in high value for the topic with minimal room for improvement. Future scholarly work could qualitatively explore student attitudes regarding the importance of SDH within the clinical curriculum to better understand the high baseline agreement for this measure.

As the survey tools were novel and internally but not externally reviewed, there may be limitations to their validity and generalizability. The curriculum could be strengthened by expansion with review, adaptation, and evaluation at other institutions.

Our findings support the incorporation of SDH education into the pediatric clerkship using a flipped classroom and cased-based approach in order to achieve student gains in reaction and learning based on Kirkpatrick’s model.[23]

Future evaluation of student behavioral change in a clinical context would be useful in analyzing higher levels on Kirkpatrick’s pyramid of evaluation for students. The curriculum is ongoing in our pediatric clerkship, and our next planned step is modification of the existing evaluation form used by facilitators in small groups to incorporate a student performance assessment measure for the SDH cases. Other future scholarly considerations include preceptor evaluation of students regarding utilization of SDH knowledge and skills in patient interactions, which could assess student behavior corresponding to Kirkpatrick’s level 3. Assessment of patient level outcomes around SDH would be ideal to achieve Kirkpatrick’s level 4 but could have significant feasibility challenges given numerous confounding factors.

Appendices

A. SDH Cases Faculty Supplements.docx
B. Curriculum Orientation.pptx
C. SDH Cases Student Handouts.docx
D. Prework - Well Child.pptx
E. Prework - Urgent Care.pptx
F. Prework - Clinical Problem-solving.pptx
G. Prework - Chronic Illness.pptx
H. Resource Assignment Orientation.pptx
I. Resource Assignment Form and Example.docx
J. Facilitator Reminder Email.docx
K. Presurvey and Case Analysis.docx
L. Postsurvey and Case Analysis.docx
M. Case Analysis Scoring Tool.docx

All appendices are peer reviewed as integral parts of the Original Publication.

Caroline Roth, MD: Assistant Professor of Clinical Pediatrics, Division of Pediatric Hospital Medicine, Department of Pediatrics, Louisiana State University Health Sciences Center New Orleans; ORCID: https://orcid.org/0009-0001-6553-2185

Copyright © 2024 Roth et al. This is an open-access publication distributed under the terms of the Creative Commons Attribution license.

Amy Prudhomme, DO: Associate Professor of Clinical Pediatrics, Division of Pediatric Hospital Medicine, Department of Pediatrics, Louisiana State University Health Sciences Center New Orleans

Kayla Griese, DO: Fellow, Pediatric Hospital Medicine, Louisiana State University Health Sciences Center New Orleans

Robbie Beyl, PhD: Statistician, Louisiana Clinical and Translational Science Center

Jessica Patrick-Esteve, MD: Associate Professor of Pediatrics, Division of Neonatology, Department of Pediatrics, Louisiana State University Health Sciences Center New Orleans

Disclosures
None to report.

Funding/Support
None to report.

Prior Presentations

Roth C, Prudhomme A. Social determinants of health curriculum for pediatric clerkship students. Poster presented at: LSU Pediatric Research Day; November 5, 2021; New Orleans, LA.

Roth C, Prudhomme A. Social determinants of health curriculum for pediatric clerkship students. Oral presentation at: Southern Regional Meeting, February 12, 2022; New Orleans, LA.

Roth C, Prudhomme A. Social determinants of health curriculum for pediatric clerkship students. Presented at Professor Rounds at: COMSEP Annual Meeting; March 29-April 1, 2022; Cincinnati, OH.

Roth C, Prudhomme A. Social determinants of health curriculum for pediatric clerkship students. Poster presented at: Pediatric Academic Societies Meeting; April 21-25, 2022; Denver, CO.

Roth C, Griese K, Prudhomme A. Social determinants of health curriculum for pediatric clerkship students. Poster presented at: Pediatric Hospital Medicine Conference; July 28-31, 2022; Orlando, FL.

Ethical Approval

The Louisiana State University Health Sciences Center New Orleans Institutional Review Board deemed further review of this project not necessary.

References

1. History of Healthy People. Health.gov. Updated August 24, 2021. Accessed October 3, 2024. https://health.gov/our-work/national-health-initiatives/healthy-people/about-healthy-people/history-healthy-people
2. Commission on Social Determinants of Health. *Closing the Gap in a Generation: Health Equity Through Action on the Social Determinants of Health. Final Report of the Commission on Social Determinants of Health*. World Health Organization; 2008.
3. About Healthy People. HealthyPeople.gov. Accessed December 28, 2021. https://www.healthypeople.gov/2020/About-Healthy-People
4. Social determinants of health. Health.gov. Accessed October 3, 2024. https://health.gov/healthypeople/objectives-and-data/social-determinants-health
5. Council on Community Pediatrics. Community pediatrics: navigating the intersection of medicine, public health, and social determinants of children's health. *Pediatrics*. 2013;131(3): 623-628. https://doi.org/10.1542/peds.2012-3933
6. Narayan A, Held M, Noelck M, Sutherell J; COMSEP Curriculum Revision Action Team. *2019 COMSEP Curriculum Revision*. COMSEP; 2019.
7. Thomas PA, Kern DE, Hughes MT, Chen BY, eds. *Curriculum Development for Medical Education: A Six-Step Approach*. 3rd ed. Johns Hopkins University Press; 2016.
8. Song AY, Baylor College of Medicine Committee on the Social Determinants of Health, Poythress EL, Bocchini CE, Kass JS. Reorienting orientation: introducing the social determinants of health to first-year medical students. *MedEdPORTAL*. 2018;14: 10752. https://doi.org/10.15766/mep_2374-8265.10752
9. Fredrick NB. Exploring health systems within the context of social determinants of health: a global health case study. *MedEdPORTAL*. 2016;12:10457. https://doi.org/10.15766/mep_2374-8265.10457
10. Fredrick N. Teaching social determinants of health through mini-service learning experiences. *MedEdPORTAL*. 2011;7:9056. https://doi.org/10.15766/mep_2374-8265.9056
11. McDonald M, West J, Israel T. From identification to advocacy: a module for teaching social determinants of health. *MedEdPORTAL*. 2015;11:10266. https://doi.org/10.15766/mep_2374-8265.10266
12. Cooper MB, Chacko M, Christner J. Incorporating LGBT health in an undergraduate medical education curriculum through the construct of social determinants of health. *MedEdPORTAL*. 2018; 14:10781. https://doi.org/10.15766/mep_2374-8265.10781
13. Bernstein R, Ruffalo L, Bower D. A multielement community medicine curriculum for the family medicine clerkship. *MedEdPORTAL*. 2016;12:10417. https://doi.org/10.15766/mep_2374-8265.10417
14. Moffett SE, Shahidi H, Sule H, Lamba S. Social determinants of health curriculum integrated into a core emergency medicine clerkship. *MedEdPORTAL*. 2019;15:10789. https://doi.org/10.15766/mep_2374-8265.10789
15. Klein M, Beck A, Kahn R, et al. Video curriculum on screening for the social determinants of health. *MedEdPORTAL*. 2013;9:9575. https://doi.org/10.15766/mep_2374-8265.9575
16. Balighian E, Burke M, Davis A, et al. A posthospitalization home visit curriculum for pediatric patients. *MedEdPORTAL*. 2020;16: 10939. https://doi.org/10.15766/mep_2374-8265.10939

Copyright © 2024 Roth et al. This is an open-access publication distributed under the terms of the Creative Commons Attribution license.

17. Essel KD, Yalamanchi S, Hysom E, Lichtenstein C. Healthy homes, healthy futures: a home visitation curriculum for pediatric residents. *MedEdPORTAL*. 2016;12:10480. https://doi.org/10.15766/mep_2374-8265.10480

18. Schmidt S, Higgins S, George M, Stone A, Bussey-Jones J, Dillard R. An experiential resident module for understanding social determinants of health at an academic safety-net hospital. *MedEdPORTAL*. 2017;13:10647. https://doi.org/10.15766/mep_2374-8265.10647

19. Fitzgerald SN, Leslie KF, Simpson R, Jones VF, Barnes ET. Culturally effective care for refugee populations: interprofessional, interactive case studies. *MedEdPORTAL*. 2018;14:10668. https://doi.org/10.15766/mep_2374-8265.10668

20. Neff J, Holmes SM, Knight KR, et al. Structural competency: curriculum for medical students, residents, and interprofessional teams on the structural factors that produce health disparities. *MedEdPORTAL*. 2020;16:10888. https://doi.org/10.15766/mep_2374-8265.10888

21. Marsh MC, Supples S, McLaurin-Jiang S, Brown CL, Linton JM. Introducing the concepts of advocacy and social determinants of health within the pediatric clerkship. *MedEdPORTAL*. 2019;15: 10798. https://doi.org/10.15766/mep_2374-8265.10798

22. Ericsson KA. Deliberate practice and acquisition of expert performance: a general overview. *Acad Emerg Med*. 2008; 15(11):988-994. https://doi.org/10.1111/j.1553-2712.2008.00227.x

23. Yardley S, Dornan T. Kirkpatrick's levels and education "evidence." *Med Educ*. 2012;46(1):97-106. https://doi.org/10.1111/j.1365-2923.2011.04076.x

Received: February 6, 2024
Accepted: July 9, 2024
Published: October 29, 2024

Copyright © 2024 Roth et al. This is an open-access publication distributed under the terms of the Creative Commons Attribution license.
